# Neutralizing Monoclonal Antibody Use and COVID-19 Infection Outcomes

**DOI:** 10.1001/jamanetworkopen.2023.9694

**Published:** 2023-04-24

**Authors:** Nalini Ambrose, Alpesh Amin, Brian Anderson, Julio Barrera-Oro, Monica Bertagnolli, Francis Campion, Daniel Chow, Risa Danan, Lauren D’Arinzo, Ashley Drews, Karl Erlandson, Kristin Fitzgerald, Melissa Garcia, Fraser W. Gaspar, Carlene Gong, George Hanna, Stephen Jones, Bert Lopansri, James Musser, John O’Horo, Steven Piantadosi, Bobbi Pritt, Raymund R. Razonable, Seth Roberts, Suzanne Sandmeyer, David Stein, Farhaan Vahidy, Brandon Webb, Jennifer Yttri

**Affiliations:** 1 The MITRE Corporation, Bedford, Massachusetts; 2 Department of Medicine, University of California, Irvine; 3 Hospital Medicine Program, University of California, Irvine; 4 Biomedical Advanced Research and Development Authority (BARDA), Administration for Strategic Preparedness and Response, US Department of Health and Human Services, Washington, District of Columbia; 5 Division of Surgical Oncology, Department of Surgery, Brigham and Women’s Hospital, Harvard Medical School, Boston, Massachusetts; 6 Department of Population Medicine, Harvard Medical School, Boston, Massachusetts; 7 Department of Radiological Sciences, University of California, Irvine; 8 Division of Infectious Diseases, Department of Medicine, Houston Methodist, Houston, Texas; 9 Houston Methodist Academic Institute, Houston, Texas; 10 Weill Cornell Medical College, New York, New York; 11 Booz Allen Hamilton in support of BARDA, Washington, District of Columbia; 12 Tunnell Government Services in support of BARDA, Princeton, New Jersey; 13 Center for Health Data Science and Analytics, Houston Methodist, Houston, Texas; 14 Division of Infectious Diseases and Clinical Epidemiology, Intermountain Health, Murray, Utah; 15 Division of Infectious Diseases, University of Utah School of Medicine, Salt Lake City; 16 Laboratory of Molecular and Translational Human Infectious Disease Research, Center for Infectious Diseases, Department of Pathology and Genomic Medicine, Houston Methodist Research Institute and Houston Methodist Hospital, Houston, Texas; 17 Department of Pathology and Laboratory Medicine, Weill Cornell Medical College, New York, New York; 18 Department of Microbiology and Immunology, Weill Cornell Medical College, New York, New York; 19 Center for Individualized Medicine–Mayo Clinic Research, Rochester, Minnesota; 20 Department of Surgery, Brigham and Women’s Hospital, Harvard Medical School, Boston, Massachusetts; 21 Department of Biological Chemistry, School of Medicine, University of California, Irvine; 22 Department of Microbiology and Molecular Genetics, School of Medicine, University of California, Irvine; 23 Department of Neurosurgery, Houston Methodist, Houston, Texas; 24 Department of Population Health Science, Weill Cornell Medical College, New York, New York

## Abstract

**Importance:**

Evidence on the effectiveness and safety of COVID-19 therapies across a diverse population with varied risk factors is needed to inform clinical practice.

**Objective:**

To assess the safety of neutralizing monoclonal antibodies (nMAbs) for the treatment of COVID-19 and their association with adverse outcomes.

**Design, Setting, and Participants:**

This retrospective cohort study included 167 183 patients from a consortium of 4 health care systems based in California, Minnesota, Texas, and Utah. The study included nonhospitalized patients 12 years and older with a positive COVID-19 laboratory test collected between November 9, 2020, and January 31, 2022, who met at least 1 emergency use authorization criterion for risk of a poor outcome.

**Exposure:**

Four nMAb products (bamlanivimab, bamlanivimab-etesevimab, casirivimab-imdevimab, and sotrovimab) administered in the outpatient setting.

**Main Outcomes and Measures:**

Clinical and SARS-CoV-2 genomic sequence data and propensity-adjusted marginal structural models were used to assess the association between treatment with nMAbs and 4 outcomes: all-cause emergency department (ED) visits, hospitalization, death, and a composite of hospitalization or death within 14 days and 30 days of the index date (defined as the date of the first positive COVID-19 test or the date of referral). Patient index dates were categorized into 4 variant epochs: pre-Delta (November 9, 2020, to June 30, 2021), Delta (July 1 to November 30, 2021), Delta and Omicron BA.1 (December 1 to 31, 2021), and Omicron BA.1 (January 1 to 31, 2022).

**Results:**

Among 167 183 patients, the mean (SD) age was 47.0 (18.5) years; 95 669 patients (57.2%) were female at birth, 139 379 (83.4%) were White, and 138 900 (83.1%) were non-Hispanic. A total of 25 241 patients received treatment with nMAbs. Treatment with nMAbs was associated with lower odds of ED visits within 14 days (odds ratio [OR], 0.76; 95% CI, 0.68-0.85), hospitalization within 14 days (OR, 0.52; 95% CI, 0.45-0.59), and death within 30 days (OR, 0.14; 95% CI, 0.10-0.20). The association between nMAbs and reduced risk of hospitalization was stronger in unvaccinated patients (14-day hospitalization: OR, 0.51; 95% CI, 0.44-0.59), and the associations with hospitalization and death were stronger in immunocompromised patients (hospitalization within 14 days: OR, 0.31 [95% CI, 0.24-0.41]; death within 30 days: OR, 0.13 [95% CI, 0.06-0.27]). The strength of associations of nMAbs increased incrementally among patients with a greater probability of poor outcomes; for example, the ORs for hospitalization within 14 days were 0.58 (95% CI, 0.48-0.72) among those in the third (moderate) risk stratum and 0.41 (95% CI, 0.32-0.53) among those in the fifth (highest) risk stratum. The association of nMAb treatment with reduced risk of hospitalizations within 14 days was strongest during the Delta variant epoch (OR, 0.37; 95% CI, 0.31-0.43) but not during the Omicron BA.1 epoch (OR, 1.29; 95% CI, 0.68-2.47). These findings were corroborated in the subset of patients with viral genomic data. Treatment with nMAbs was associated with a significant mortality benefit in all variant epochs (pre-Delta: OR, 0.16 [95% CI, 0.08-0.33]; Delta: OR, 0.14 [95% CI, 0.09-0.22]; Delta and Omicron BA.1: OR, 0.10 [95% CI, 0.03-0.35]; and Omicron BA.1: OR, 0.13 [95% CI, 0.02-0.93]). Potential adverse drug events were identified in 38 treated patients (0.2%).

**Conclusions and Relevance:**

In this study, nMAb treatment for COVID-19 was safe and associated with reductions in ED visits, hospitalization, and death, although it was not associated with reduced risk of hospitalization during the Omicron BA.1 epoch. These findings suggest that targeted risk stratification strategies may help optimize future nMAb treatment decisions.

## Key Points

**Question:**

Are neutralizing monoclonal antibody (nMAb) therapies associated with reduced risk of adverse outcomes of COVID-19 in subpopulations at high risk of poor outcomes and across multiple SARS-CoV-2 variant epochs?

**Findings:**

In this cohort study that included 167 183 nonhospitalized patients with COVID-19 who were eligible for nMAb treatment, the association between nMAbs and reduced risk of poor outcomes was strongest among immunocompromised and unvaccinated patients. Combining multiple factors into a risk estimation model to stratify patients further highlighted differences in outcomes; by variant, nMAb treatment was associated most strongly with reduced risk of poor outcomes in patients infected with the Delta variant, whereas the associations were weaker in patients infected with the Omicron BA.1 variant.

**Meaning:**

These findings suggest that risk-targeting strategies are important for optimizing outcomes in patients receiving nMAbs for the treatment of COVID-19.

## Teaser

This cohort study assesses the safety of neutralizing monoclonal antibodies and their association with reduced risk of adverse outcomes among nonhospitalized patients with COVID-19 from a consortium of 4 US health care systems across multiple variant epochs.

## Introduction

SARS-CoV-2, the cause of COVID-19, may result in severe disease associated with high rates of hospitalization and death. Therapy with neutralizing monoclonal antibodies (nMAbs) that bind to the spike protein of SARS-CoV-2 has been shown to prevent hospitalization and death in randomized clinical trials of nonhospitalized, nonvaccinated patients at high risk of poor outcomes.^1,2,3,4^ To date, several nMAb therapies have been granted emergency use authorization (EUA) by the US Food and Drug Administration.

Building on evidence from randomized clinical trials, continued monitoring of the effectiveness and safety of nMAb therapies is important to inform policy recommendations and clinical practice.^5^ Previous retrospective cohort studies on nMAbs have used data from electronic health records (EHRs), and the results of these studies have revealed low rates of adverse events and reduced risks of disease progression and severity, hospitalization, emergency department (ED) visits, intensive care unit admissions, and all-cause death.^6,7,8,9,10,11,12,13,14,15^ However, previous studies have been limited by sample size, homogenous populations, short study periods, lack of contemporaneous control groups, or lack of SARS-CoV-2 variant information. This retrospective cohort study assessed the safety of 4 nMAb products (bamlanivimab, bamlanivimab-etesevimab, casirivimab-imdevimab, and sotrovimab) and their association with reduced risk of adverse outcomes, stratified by clinical risk characteristics and SARS-CoV-2 variants, using clinical and viral sequencing data from a consortium of 4 geographically and demographically diverse US health care systems.

## Methods

### Research Consortium Structure

Four US academic health care systems based in California, Minnesota, Texas, and Utah contributed individual patient data to a cloud-based centralized registry using a shared data model (the Observational Medical Outcomes Partnership model^16^) adapted to address study goals and safe harbor deidentification standards (per the Health Insurance Portability and Accountability Act, Security and Privacy; 45 CFR §164.514[b][2]^17^) (eMethods in Supplement 1). Study design and methods were approved by the institutional review boards at the organization conducting this cohort study (The MITRE Corporation) and at the 4 health systems (University of California–Irvine Medical Center, Center for Individualized Medicine–Mayo Clinic Research [Minnesota], Houston Methodist [Texas], and Intermountain Healthcare [Utah]) participating in the study. The need for informed consent was waived because this study used deidentified data. This study followed the Strengthening the Reporting of Observational Studies in Epidemiology (STROBE) reporting guideline for cohort studies.

### Study Population

The cohort comprised 167 183 nonhospitalized patients 12 years and older. Patients were included if they had a laboratory-confirmed positive COVID-19 polymerase chain reaction or antigen test collected in the noninpatient setting between November 9, 2020, and January 31, 2022, or if they were referred to the health care system specifically for nMAb treatment. All patients in the cohort met at least 1 of the following Food and Drug Administration EUA criteria for high risk of a poor outcome: 65 years or older, pregnant, and/or having obesity or overweight, chronic kidney disease, diabetes, immunosuppressive disease or immunosuppressive treatment, cardiovascular disease or hypertension, chronic lung diseases, sickle cell disease, neurodevelopmental disorders (eg, cerebral palsy), and/or medical-related technological dependence.^18^ The patient index date was the date of the first positive test result (99.4% of population) or the date of referral (0.6% of population). Patient index dates were categorized into the following variant epochs: pre-Delta (November 9, 2020, to June 30, 2021), Delta (July 1 to November 30, 2021), Delta and Omicron BA.1 (December 1 to 31, 2021), and Omicron BA.1 (January 1 to 31, 2022).

**Figure 1.**
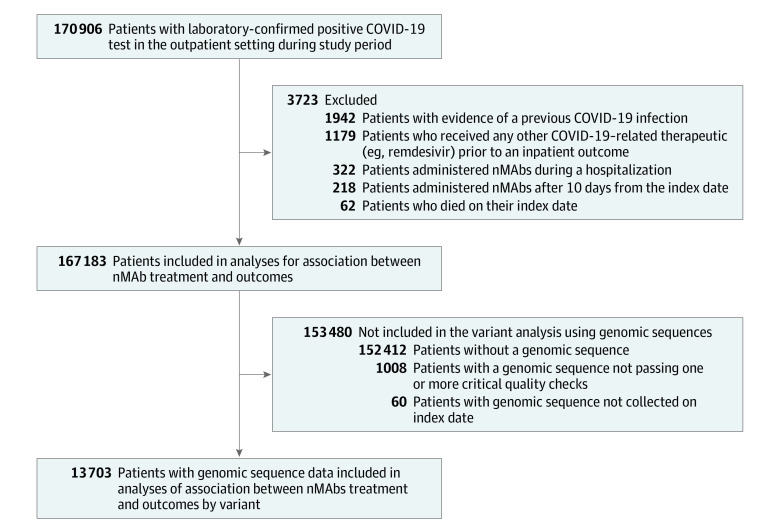
Cohort Flow Diagram for Main Analysis and Variant Analysis Using Genomic Sequences nMAb indicates neutralizing monoclonal antibody.

Patients were excluded from analyses if they died on the index date, had clinical COVID-19 before the index date, received another COVID-19 outpatient therapy within the outcome window of 30 days, or received nMAb treatment more than 10 days after the index date or during a hospitalization (Figure 1). Clinical COVID-19 was defined as having a previously recorded *International Statistical Classification of Diseases, Tenth Revision, Clinical Modification *code U07.1 diagnosis without a positive laboratory test. COVID-19 immunization records were obtained from EHR and state vaccine registries. Patients were considered partially vaccinated if they received 1 mRNA-1273 (Moderna) or BNT162b2 (Pfizer-BioNTech) vaccine dose and fully vaccinated if they received 1 Ad26.COV2.S (Janssen) vaccine dose or 2 mRNA or BNT162b2 vaccine doses. Patients were considered boosted if they had 1 additional vaccine dose after being fully vaccinated. Patients were considered immunocompromised if they received immunosuppressant medications within the previous 90 days or a diagnosis of immune deficiency or solid organ or hematopoietic stem cell transplant.^19^ Immunosuppressive medications were defined using systemic corticosteroid and immunosuppressant value sets,^20,21^ which were updated to include drugs available after the version date of the value sets. To characterize the representativeness of the study population, race (American Indian or Alaska Native, Asian, Black or African American, Native Hawaiian or other Pacific Islander, White, other, and not specified) and ethnicity (Hispanic or Latino, Non-Hispanic or non-Latino, and not specified) categories were abstracted from the EHRs.

### Genomic Sequence Information

SARS-CoV-2 genome sequence information was obtained for a subset of patients from each health care system. FASTQ files^22^ were processed through a common pipeline to produce World Health Organization (WHO) variant calls for all SARS-CoV-2 variants and Nextstrain clade classifications^23^ for Delta variants. The study end date preceded the emergence of the Omicron BA.2 subvariant; therefore, hereafter, Omicron BA.1 signifies both BA.1 and BA.1 sublineages. Genomic sequence information was only analyzed if the sample was collected on the index date and the sequence passed quality checks (eTable 1 and eTable 2 in Supplement 1). Variant calls not within the Alpha, Delta, Epsilon, or Omicron BA.1 variant groups were classified as *other* in the outcome analyses. Details on the samples, processing pipeline, and data quality controls for viral sequence data are described in the eMethods in Supplement 1. Although patients with SARS-CoV-2 genome sequence information were a nonrandom subset, limited by resource constraints, patients with a variant call had demographic and clinical characteristics similar to those of the main study population (eTable 3 in Supplement 1).

### Outcomes

Four effectiveness outcomes were assessed: all-cause ED visits, hospitalization, death, and a composite of hospitalization or death. Outcomes were assessed at 14 days and 30 days from a patient’s index date. Emergency department visits that occurred on the index date or treatment date were not considered as outcomes. The study results focused on ED visits within 14 days, hospitalization within 14 days, death within 30 days, and the composite outcome of hospitalization or death within 30 days due to their clinical relevance. Potential adverse drug events (ADEs) were identified using electronic queries as well as health care system ADE reporting systems. Investigators (D.C., S.J., J.O., and B.W.) confirmed each ADE and assigned a standardized severity.

### Sensitivity Analyses

To assess whether indication bias was minimized across variant epochs, 4 sensitivity analyses were performed: (1) covariate balance in the Omicron BA.1 epoch was compared with that of other epochs, (2) propensity models were fit within each variant epoch to evaluate covariate balance and effect estimates by epoch, (3) association with outcomes was estimated using exact matching, and (4) association with outcomes was estimated using a refined classification of immune compromise severity. In addition, the influence of immortal time bias was investigated by shifting the index date of treated patients to the day of nMAb treatment.^24^ Details are provided in the eAppendix in Supplement 1.

### Statistical Analysis

The primary analysis of outcomes was conducted using causal inference methods to minimize the influence of confounding. To do this, missing data (comprising <3% of all variables) were replaced with imputed values using predictive mean matching to generate 10 data sets (eMethods in Supplement 1).^25^ A propensity model estimating the probability of nMAb treatment vs nontreatment was developed from more than 100 candidate variables (eFigure 1 in Supplement 1). Multiple candidate propensity models were developed (eTables 4, 5, and 6 in Supplement 1) and evaluated for optimal reduction of covariate imbalance between treated and nontreated groups, measured by standardized mean differences and the Kolmogorov-Smirnoff statistic.^26^ The optimal propensity model chosen was an ℓ1 regularized logistic regression model (eTable 7 in Supplement 1) in which all covariates had an absolute standardized mean difference of less than 0.1 (eFigure 2 in Supplement 1).

Marginal structural models (MSMs) were used to estimate treatment effectiveness.^27,28,29,30,31,32^ These models estimate effect sizes using weighted logistic regression in which the variables of the regression model are the treatment, the effect modifiers of interest, and the products of the treatment and the effect modifiers.^27,28,29^ Robins^33^ derived MSM weights for general treatment and observation processes, allowing for multiple treatment types given at multiple time points and fixed time and time-to-event end points. A compound model that separates the decision to treat from treatment selection was used to compute the MSM weights.^34^ Rubin rules^35,36,37^ were used to pool effect estimates and SEs obtained by the sandwich estimator from the 10 imputed data sets.

Subgroup analyses by COVID-19 vaccination status, immunocompromised status, risk of severe COVID-19, variant epoch, and WHO variant classifications were calculated using these subgroups as effect modifiers in the MSM.^27,31^ For the subgroup analysis of patients by estimated risk of severe COVID-19, a disease risk score (DRS) model was developed to classify patients based on risk of hospitalization or death within 30 days. The DRS model was specified using a hyperparameter grid search in a training set of nontreated patients (eTable 8 in Supplement 1) and validated in a holdout set of nontreated patients (eTable 9 in Supplement 1). The optimal model was then used to categorize all patients into 5 risk strata based on risk quintiles, with stratum 1 representing the lowest risk. The nMAb product (eg, bamlanivimab or sotrovimab) was considered a time-varying effect modifier; therefore, effect estimates by nMAb product were assessed within variant epoch and sequence subgroups.

Statistical significance was set at 2-tailed α = .05. The Firth logistic regression model was used when outcomes were rare within subgroups (<0.01% of patients).^38,39^ Multiple testing correction was not performed. Statistical analyses were performed using R software, version 4.2.1 (R Foundation for Statistical Computing), and Python software, version 3.6 (Python Software Foundation).

## Results

### Patient Characteristics

Among 167 183 patients, the mean (SD) age was 47.0 (18.5) years, 95 669 patients (57.2%) were female at birth, 139 379 (83.4%) were White, and 23 615 (14.1%) were Hispanic. By variant epoch, 62 443 patients (37.4%) were diagnosed in the pre-Delta epoch, 44 409 (26.6%) in the Delta epoch, 17 921 (10.7%) in the Delta and Omicron BA.1 epoch, and 42 410 (25.4%) in the Omicron BA.1 epoch (Table 1; eFigure 3 in Supplement 1). A total of 25 241 patients (15.1%) were treated with nMAbs; of those, 16 640 (65.9%) received casirivimab-imdevimab, 4735 (18.8%) received bamlanivimab, 1948 (7.7%) received bamlanivimab-etesevimab, and 1918 (7.6%) received sotrovimab. Treatment with nMAbs was administered with the greatest frequency in September 2021 (3799 patients [15.1%]) (eFigure 4 in Supplement 1).

**Table 1.** Demographic and Clinical Characteristics of Study Population

Characteristic	Patients, No. (%)	
	Nontreated (n = 141 942)	Treated (n = 25 241)
Age, mean (SD), y	45.4 (18.3)	56.0 (16.9)
Sex		
Female	82 279 (58.0)	13 390 (53.0)
Male	59 658 (42.0)	11 851 (47.0)
Not specified	5 (<0.1)	0
Race		
American Indian or Alaska Native	1206 (0.8)	185 (0.7)
Asian	3769 (2.7)	521 (2.1)
Black or African American	9593 (6.8)	1153 (4.6)
Native Hawaiian or other Pacific Islander	1607 (1.1)	193 (0.8)
White	117 286 (82.6)	22 093 (87.5)
Other^a^	5659 (4.0)	743 (2.9)
Not specified	2822 (2.0)	353 (1.4)
Ethnicity		
Hispanic or Latino	21 154 (14.9)	2461 (9.8)
Non-Hispanic or non-Latino	116 714 (82.2)	22 186 (87.9)
Not specified	4074 (2.9)	594 (2.4)
COVID-19 vaccination status		
No record of vaccination	89 983 (63.4)	14 971 (59.3)
Partially vaccinated	3693 (2.6)	710 (2.8)
Fully vaccinated	35 828 (25.2)	8456 (33.5)
Fully vaccinated and boosted	12 418 (8.7)	1095 (4.3)
Variant epoch^b^		
Pre-Delta	55 141 (38.8)	7302 (28.9)
Delta	30 237 (21.3)	14 172 (56.1)
Delta and Omicron BA.1	15 215 (10.7)	2706 (10.7)
Omicron BA.1	41 349 (29.1)	1061 (4.2)
Smoker	9131 (6.4)	1560 (6.2)
Pregnant	4654 (3.3)	489 (1.9)
Immunocompromised	11 101 (7.8)	3920 (15.5)
Comorbidities		
Obesity	58 928 (41.5)	11 980 (47.5)
Hypertension	26 211 (18.5)	8832 (35.0)
Diabetes	13 019 (9.2)	4565 (18.1)
Chronic pulmonary disease	12 337 (8.7)	3208 (12.7)
Thrombotic or hematological disorders	8516 (6.0)	2750 (10.9)
Hypothyroidism	8647 (6.1)	2664 (10.6)
Cancer	5341 (3.8)	2331 (9.2)
Chronic kidney disease	4839 (3.4)	2261 (9.0)
Peripheral vascular disease	5209 (3.7)	2222 (8.8)
Heart failure	3138 (2.2)	1535 (6.1)
Neurological disorders or paralysis	5378 (3.8)	1420 (5.6)
Chronic liver disease	4493 (3.2)	1331 (5.3)
Valvular heart disease	2058 (1.4)	1182 (4.7)

^a^ Patients in the other race category identified as multiracial or as not within the American Indian or Alaska Native, Asian, Native Hawaiian or other Pacific Islander, or White race.

^b^ The pre-Delta epoch was defined as November 9, 2020, to June 30, 2021; the Delta epoch as July 1 to November 30, 2021; the Delta and Omicron BA.1 epoch as December 1 to 31, 2021; and the Omicron BA.1 epoch as January 1 to 31, 2022.

Compared with nontreated patients, treated patients were older and more likely to be male, White, and non-Hispanic. Treated patients were more likely to have comorbidities than nontreated patients, including immunocompromised status (3920 patients [15.5%] vs 11 101 patients [7.8%]). This difference was greatest during the Omicron BA.1 epoch, in which 399 treated patients (37.6%) were immunocompromised compared with 3712 nontreated patients (9.0%) (eTable 10 in Supplement 1).

The date of COVID-19 symptom onset was missing for 124 529 patients (74.5%) and, when available, was reported to be the index date for 30 080 patients (70.5%). The mean (SD) time from the index date to nMAb treatment was 2.4 (1.5) days, and the median time was 2 days (range, 1-3 days). The most common WHO variants among 13 703 patients with genomic sequences were the Alpha variant (1085 patients [7.9%]), the Delta 21AI (925 patients [6.8%]) and Delta 21J (7778 [56.8%]) clades, and the Omicron BA.1 variant (2582 patients [18.8%]) (eTable 11 and eFigure 5 in Supplement 1). Of the 2582 Omicron variant calls, 99.9% were BA.1 subvariants and their sublineages, including 1131 BA.1.1 and 832 BA.1.15.

### Association of nMAb Treatment With Adverse Outcomes

Overall, nMAb treatment was associated with decreased risks of ED visits, hospitalization, and death at both the 14-day and 30-day end points (Table 2). The odds of ED visits within 14 days of the index date were 24% lower in treated vs nontreated patients (odds ratio [OR], 0.76; 95% CI, 0.68-0.85). The odds of hospitalization within 14 days were 48% lower in treated vs nontreated patients (OR, 0.52; 95% CI, 0.45-0.59), with an estimated number needed to treat (NNT) to prevent 1 hospitalization of 43. The largest reduction in odds was observed for death. For example, the odds of death within 30 days were 86% lower in treated vs nontreated patients (OR, 0.14; 95% CI, 0.10-0.20). Results were confirmed in our sensitivity analysis testing the influence of immortal time bias (eAppendix in Supplement 1).

**Table 2.** Overall Association of Neutralizing Monoclonal Antibodies With Adverse Outcomes

Outcome	End point, d	OR (95% CI)	Probability, % (95% CI)		NNT
			Nontreated	Treated	
All-cause ED visit	14	0.76 (0.68-0.85)	4.6 (4.3-5.0)	3.6 (3.3-3.9)	93
	30	0.84 (0.76-0.92)	5.9 (5.6-6.3)	5.0 (4.7-5.4)	109
All-cause hospitalization	14	0.52 (0.45-0.59)	4.9 (4.6-5.2)	2.6 (2.3-2.9)	43
	30	0.64 (0.57-0.72)	5.9 (5.5-6.2)	3.8 (3.5-4.2)	50
All-cause death	14	0.12 (0.07-0.20)	0.5 (0.4-0.6)	0.1 (0-0.1)	245
	30	0.14 (0.10-0.20)	0.9 (0.7-1.1)	0.1 (0.1-0.2)	131
Composite outcome: hospitalization or all-cause death	14	0.49 (0.43-0.55)	5.3 (4.9-5.6)	2.6 (2.4-2.9)	38
	30	0.61 (0.54-0.68)	6.3 (6.0-6.7)	3.9 (3.6-4.3)	42

Abbreviations: ED, emergency department; NNT, number needed to treat; OR, odds ratio.

**Figure 2.**
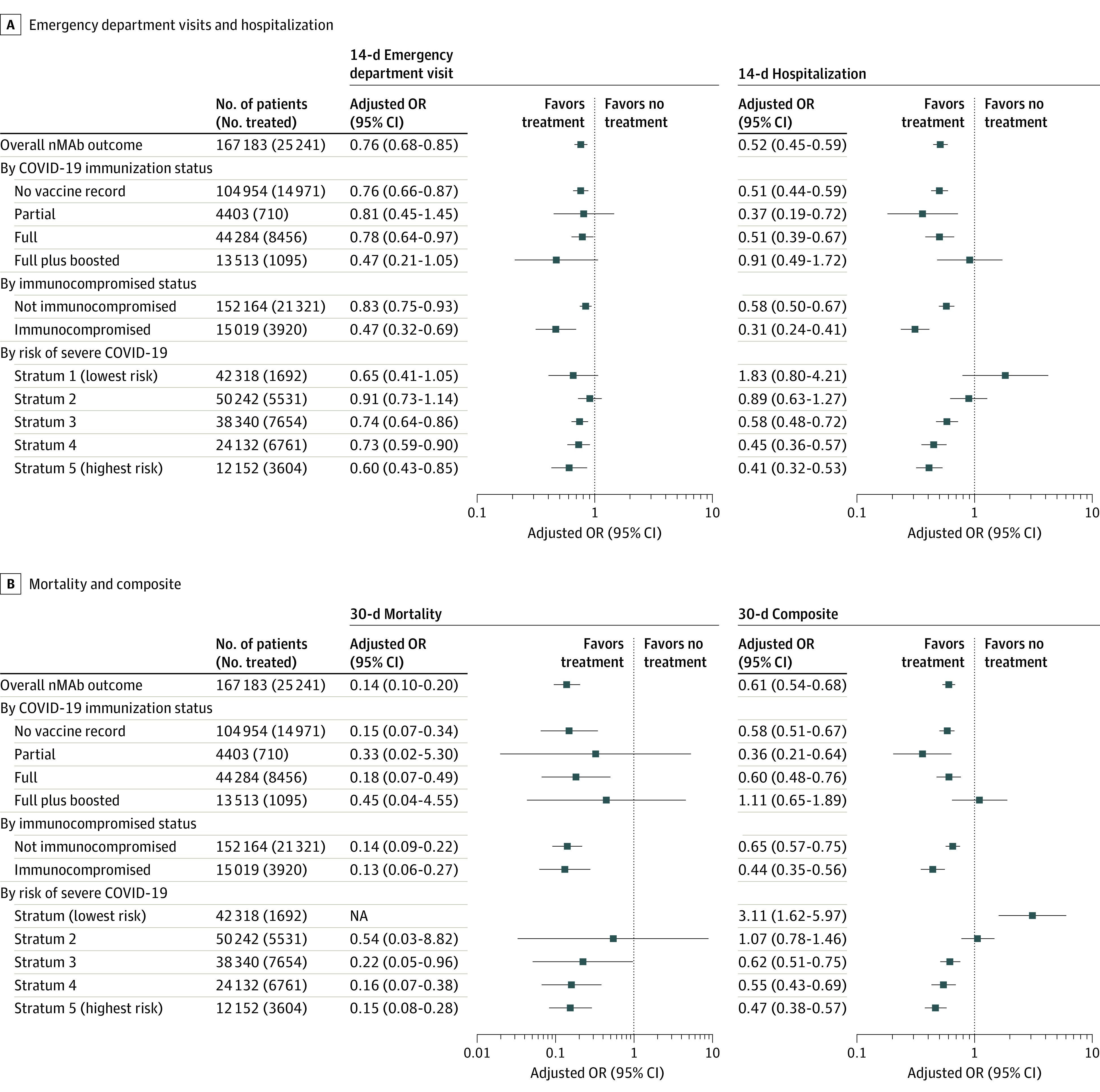
Association of Neutralizing Monoclonal Antibodies (nMAbs) With Adverse Outcomes by Risk Factor An odds ratio (OR) less than 1 indicates reduced risk, and an OR greater than 1 indicates treatment was associated with an adverse outcome. The 30-day composite outcome comprised hospitalization or death. NA indicates not applicable.

By COVID-19 vaccination status, the association between nMAb and reduced risk was stronger among unvaccinated patients vs vaccinated patients. For example, among unvaccinated vs fully vaccinated patients, the NNTs were 35 vs 60 to prevent 1 hospitalization within 14 days, 123 vs 146 to prevent 1 death within 30 days, and 32 vs 51 to prevent the composite outcome of hospitalization or death within 14 days (eTable 12 in Supplement 1). However, the odds of hospitalization within 14 days were 49% lower in both groups (unvaccinated: OR, 0.51 [95% CI, 0.44-0.59]; fully vaccinated: OR, 0.51 [95% CI, 0.39-0.67]) (Figure 2). Treatment with nMAbs was associated with significantly lower odds of death at 14 days and 30 days in both unvaccinated patients (14 days: OR, 0.18 [95% CI, 0.06-0.53]; 30 days: OR, 0.15 [95% CI, 0.07-0.34]) and fully vaccinated patients (14 days: OR, 0.11 [95% CI, 0.02-0.58]; 30 days: OR, 0.18 [95% CI, 0.07-0.49]). Irrespective of treatment, vaccines were associated with fewer ED visits and hospitalizations (eTable 12 in Supplement 1). For example, the risk-adjusted probability of hospitalization within 14 days was 6.0% (95% CI, 5.7%-6.4%) for unvaccinated nontreated patients and 3.4% (95% CI, 2.9%-4.1%) for fully vaccinated nontreated patients. No significant preventive benefit for ED visits, hospitalization, or death was observed among patients who were both fully vaccinated and boosted. Treatment with nMAbs was also associated with reduced risk of adverse outcomes among immuncompromised patients (eTable 13 in Supplement 1), with an OR for hospitalization within 14 days of 0.31 (95% CI, 0.24-0.41; NNT, 17) and an OR for death within 30 days of 0.13 (95% CI, 0.06-0.27; NNT, 73).

The association of nMAb treatment with reduced risk of poor outcomes was greater among patients with an increasingly higher baseline probability of poor outcomes as defined by the DRS (Figure 2; eTable 14 in Supplement 1). For example, nMAb treatment was associated with an incrementally greater benefit in reducing hospitalizations within 14 days among those in the third risk stratum (OR, 0.58; 95% CI, 0.48-0.72), fourth risk stratum (OR, 0.45; 95% CI, 0.36-0.57), and fifth risk stratum (OR, 0.41; 95% CI, 0.32-0.53). As the probability of a poor outcome increased with each sequential DRS risk stratum, the strength of the association between nMAb treatment and reduced risk also increased. For example, the NNT to prevent 1 hospitalization within 14 days was 66 among patients in the third risk stratum, 25 among patients in the fourth risk stratum, and 12 among patients in the fifth risk stratum. A similar result was observed for mortality, for which the NNT to prevent 1 death at 30 days was only 29 in the highest (fifth) risk stratum.

**Figure 3.**
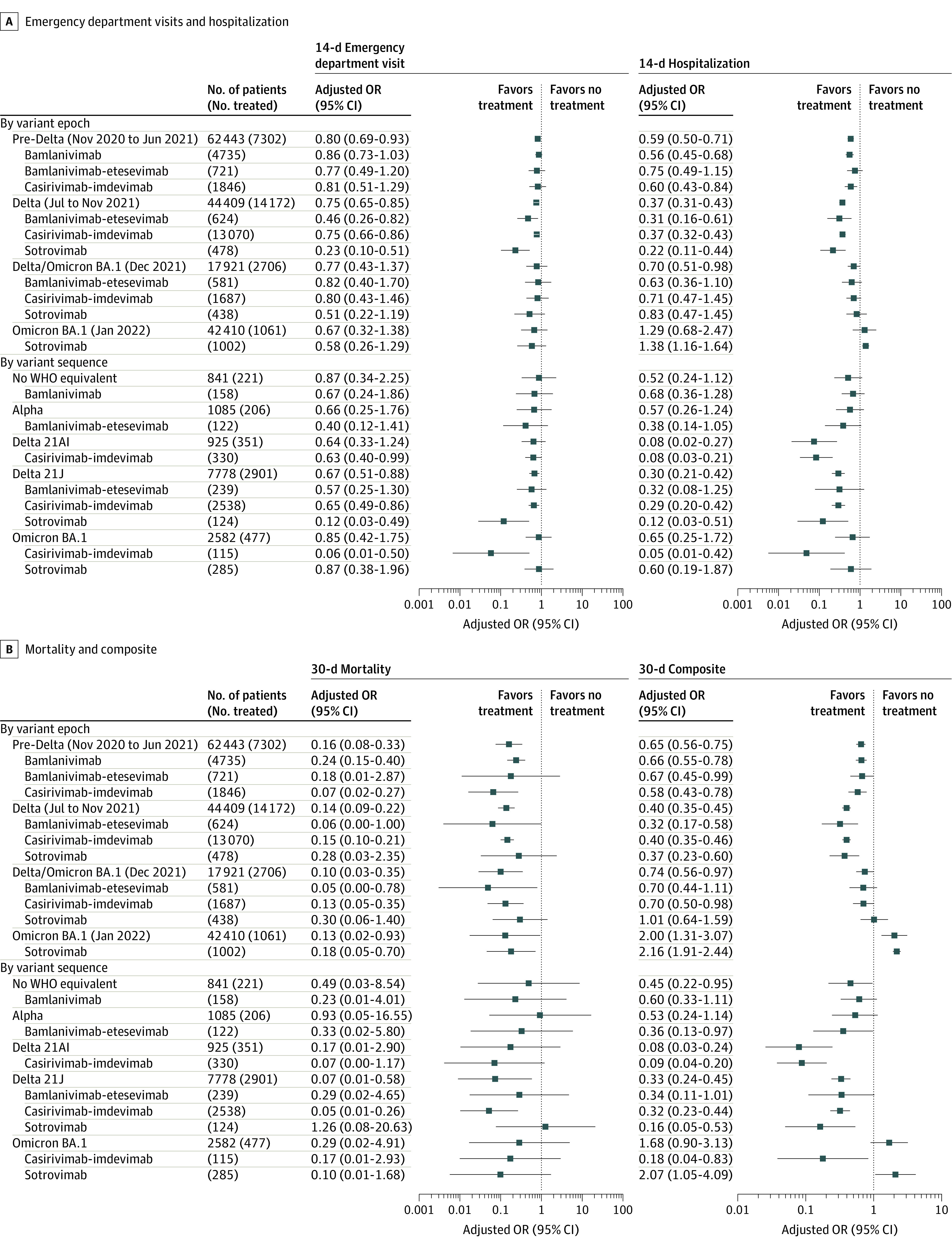
Associationof Neutralizing Monoclonal Antibodies With Adverse Outcomes by Variant Epoch, Variant Sequence, and Neutralizing Monoclonal Antibody Product Odds ratios (ORs) are only shown when at least 100 patients were treated with a neutralizing monoclonal antibody product per variant epoch or sequence. An OR less than 1 indicates reduced risk, and an OR greater than 1 indicates treatment was associated with an adverse outcome. The 30-day composite outcome comprised hospitalization or death. WHO indicates World Health Organization.

Treatment with nMAbs was associated with lower odds of an ED visit within 14 days in the pre-Delta epoch (OR, 0.80; 95% CI, 0.69-0.93) and the Delta epoch (OR, 0.75; 95% CI, 0.65-0.85) but not in the Delta and Omicron BA.1 epoch (OR, 0.77; 95% CI, 0.43-1.37) or the Omicron BA.1 epoch (OR, 0.67; 95% CI, 0.32-1.38) (Figure 3; eTable 15 in Supplement 1). Similarly, the reduction in hospitalizations within 14 days after nMAb treatment was greatest in the Delta epoch (OR, 0.37; 95% CI, 0.31-0.43) followed by the pre-Delta epoch (OR, 0.59; 95% CI, 0.50-0.71) and the Delta and Omicron BA.1 epoch (OR 0.70; 95% CI, 0.51-0.98) but was not significant during the Omicron BA.1 epoch (OR, 1.29; 95% CI, 0.68-2.47). The risk-adjusted probability of hospitalization within 14 days for nontreated patients was 5.2% (95% CI, 4.8%-5.8%) in the pre-Delta epoch, 6.2% (95% CI, 5.7%-6.9%) in the Delta epoch, 3.5% (95% CI, 2.9%-4.3%) in the Delta and Omicron BA.1 epoch, and 1.7% (95% CI, 1.5%-1.9%) in the Omicron epoch. Notably, in the Omicron BA.1 epoch, treated patients had significantly higher odds of hospitalization within 30 days (OR, 2.10; 95% CI, 1.37-3.21) and the composite outcome of hospitalization or death within 30 days (OR, 2.00; 95% CI, 1.31-3.07) than nontreated patients. However, post hoc sensitivity analyses found that the higher odds of hospitalization within 30 days among treated patients was not robust to additional methods of estimating effectiveness, including exact matching (eTables 20-29 in Supplement 1). Treatment with nMAbs was associated with a significant reduction in the odds of death within 30 days in each of the 4 variant epochs (pre-Delta: OR, 0.16 [95% CI, 0.08-0.33]; Delta: OR, 0.14 [95% CI, 0.09-0.22]; Delta and Omicron BA.1: OR, 0.10 [95% CI, 0.03-0.35]; and Omicron BA.1: OR, 0.13 [95% CI, 0.02-0.93]). The greatest association between nMAb treatment and reduced risk of death occurred in the Delta epoch, in which the NNT to prevent 1 death within 30 days was 97. In general, the different nMAb products used within an epoch did not significantly differ with regard to their association with reduced risk of adverse outcomes (Figure 3; eTable 16 in Supplement 1). These findings were corroborated in the subset of patients with viral genomic data (eTable 17 in Supplement 1).

The analysis of individual nMAb products and the variant sequenced from the corresponding patient did not reveal any significant differences between nMAb products (Figure 3; eTable 18 and eTable 19 in Supplement 1). Casirivimab-imdevimab was associated with a significantly reduced risk of hospitalization within 14 days among patients with the Delta 21AI variant (OR, 0.08; 95% CI, 0.03-0.21), as was sotrovimab among patients with the Delta 21J variant (OR, 0.12; 95% CI, 0.03-0.51). Casirivimab-imdevimab was also associated with lower odds of hospitalization within 14 days (OR, 0.05; 95% CI, 0.01-0.42) in a small sample of 115 patients infected with sequence-confirmed Omicron BA.1.

### Safety

Thirty-eight treated patients (0.2% of all treated patients) were identified as having 1 or more ADEs. Of those, 8 patients (21.1%) had at least 1 ADE categorized as severe, 14 (36.8%) had at least 1 ADE categorized as moderate, and 16 (42.1%) had at least 1 ADE categorized as mild. The overall proportion of patients experiencing an ADE was similar across nMAb products (8 patients [0.2%] receiving bamlanivimab, 1 patient [0.1%] receiving bamlanivimab-etesevimab, 26 patients [0.2%] receiving casirivimab-imdevimab, and 3 patients [0.2%] receiving sotrovimab).

## Discussion

In this multicenter retrospective cohort study, we found that nMAbs for the treatment of nonhospitalized patients with COVID-19 were associated with reduced risk of ED visits, hospitalization, and death. This study is noteworthy because of its large sample size, long study period, and inclusion of viral genomic data. We observed important differences in estimatedeffectiveness relative to patient characteristics; these differences have implications for which patients are most likely to benefit from treatment. For example, the association of nMAbs with outcomes varied by vaccination status, with the lowest NNTs observed in unvaccinated patients. Treatment with nMAbs was likely more efficient among unvaccinated patients than fully vaccinated patients due to a higher risk-adjusted baseline probability of hospitalization within 14 days (6.0% vs 3.4%). Associations were also greater among immunocompromised patients. Combining these and other risk factors using the DRS, we found that the association of nMAb treatment with reduced risk of adverse outcomes increased incrementally as the overall baseline risk of a poor outcome increased, suggesting that risk-targeted patient selection strategies beyond the EUA may be an important tool for allocating nMAb products to optimize outcomes, especially when nMAb supply is limited relative to demand.

We observed important differences in outcomes by variant. Relative to other variant epochs, nMAb treatment was not associated with reduced risk of ED visits and hospitalizations during the Omicron BA.1 epoch, with only the mortality benefit remaining statistically significant, adding to recent mixed findings of sotrovimab’s association with reduced risk of hospitalizations during the Omicron surge.^40,41,42,43^ This may be related to the decoupling phenomenon observed during the Omicron epoch, in which the incidence of severe disease and death decreased substantially relative to the number of new cases partially due to increasing net population immunity from vaccination and natural infection.^44^ As the baseline probability of hospitalization or death decreases, so does the potential benefit of therapies, including nMAbs. In this study, the risk-adjusted probability of hospitalization within 14 days among nontreated patients decreased from 5.2% in the pre-Delta epoch and 6.2% in the Delta epoch to 1.7% in the Omicron BA.1 epoch, during which the majority of patients were fully vaccinated or boosted. A similar change in outcomes among vaccinated patients during the Omicron epoch has recently been reported for nirmatrelvir-ritonavir.^45^

Another possible explanation for the decrease in nMAb treatment’s association with reduced risk of poor outcomes may be related to the shift in causes of admissions reported during the Omicron BA.1 epoch. During this time, previous research^46^ found that 20% or more of admissions with SARS-CoV-2 infection were due to non–COVID-related issues, such as exacerbations of chronic conditions. These non–COVID-19 admissions (1) were more common among patients with high risk of poor outcomes who were eligible for nMAb treatment, (2) were more likely to occur later (between day 15 and day 30 after symptom onset) than COVID-19–related admission, and (3) may not be preventable with nMAb treatment. The shift in reasons for admission may explain the directionality of effect estimates for 30-day hospitalization in the Omicron BA.1 epoch, which appear to reflect harm, whereas 14-day hospitalization effect estimates were not significant. In addition, this observation may have been partly due to unmeasured confounders distinct to the Omicron BA.1 epoch, although, in multiple sensitivity analyses, we did not detect significant temporal imbalances in more than 100 measured covariates.

The changes in outcomes associated with nMAb treatment observed over the study period highlight the value of maintaining large consortiums to continually monitor responses to therapy for infectious agents in which effectiveness may change rapidly with disease evolution. This approach is particularly valuable to monitor postrelease effectiveness and safety of therapies granted EUA during a pandemic. Despite the fact that no nMAb products currently have EUA for outpatient COVID-19 treatment, the variation in effect sizes by a priori risk of a poor outcome has important clinical implications for future COVID-19 and other disease treatment practices. This study’s results revealed that when demand for a therapy exceeds supply, risk targeting is an important approach to identify individuals who would benefit most from treatment. Furthermore, even when there is no scarcity of supply, it is important for clinicians to treat patients who will benefit most, especially in scenarios in which a patient may experience an adverse event or a financial burden due to accepting treatment.

### Limitations

This study has several limitations, including a risk of missing outcome data because ED visits and inpatient stays outside of the participating health care systems were unlikely to be captured, and some deaths occurring in the home or in distant facilities might not have been identified. This study did not examine specific causes of death or primary diagnoses of ED or inpatient visits. Some potential confounders could not be abstracted from available EHR data, including income, primary language spoken, cancer stage, and functional status. In addition, measures of acute infection severity, such as fever, oxygen saturation, or white blood cell count, were not used in the models. Furthermore, due to reidentification concerns,^17^ geographic location was limited to 3-digit zip codes. Although we were able to collect adverse event data as required for therapies issued under an EUA, we were unable to capture more granular data required to identify lower-grade adverse events, and overall reporting was low.

## Conclusions

This cohort study found that nMAbs for the treatment of COVID-19 were generally safe and associated with reductions in adverse outcomes including ED visits, hospitalization, and death, with patients at greatest risk of severe COVID-19 complications benefiting most from treatment. These findings suggest that targeted risk stratification strategies may help optimize future nMAb treatment decisions.

## Notes

Supplement 1. **eMethods.** Data Model, Modeling Variable Definitions, Genomic Sequence Methodology, Missing Data Imputation, Propensity Model, and Disease Risk Score **eTable 1.** Thresholds for Genomic Data Quality **eTable 2.** Genomics Pipeline Steps in Order of Execution With Data Quality Check Outputs Identified **eTable 3.** Comparison of Demographic and Clinical Characteristics Between Patients With a Variant Call (n = 13 703) and Entire Study Population (n = 167 183) **eFigure 1.** Variable Groupings Used in Predictive Mean Matching, Propensity Score, and Disease Risk Score Models **eTable 4.** Hyperparameter Options Implemented for the Logistic Regression Grid Search **eTable 5.** Hyperparameter Options Implemented for the GBT Grid Search **eTable 6.** Hyperparameter Options Implemented for the RF Grid Search **eFigure 2.** Covariate Balance Plots Using Stabilized Weights **eTable 7.** Propensity Score Model Statistics **eTable 8.** Hyperparameter Options Implemented for the Logistic Regression Grid Search **eTable 9.** Metrics Used to Evaluate the Logistic Regression Models **eFigure 3.** Number of Patients by Treatment Category and Index Date Year and Month **eFigure 4.** nMAb Products Administered Through Time **eTable 10.** Percent of Nontreated and Treated Patients Considered Immunocompromised by Index Data Year and Month **eTable 11.** WHO Variant Classifications From Subset of Patients With Genome Sequences (n = 13 703) by Treatment Group **eFigure 5.** Distribution of WHO Variant Call From Genomic Sequences by Index Date Year and Month **eTable 12.** Treatment Effectiveness of nMAbs by COVID-19 Vaccination Status on 14- and 30-Day Outcomes **eTable 13.** Treatment Effectiveness of nMAbs by Immunocompromised Status on 14- and 30-Day Outcomes **eTable 14.** Treatment Effectiveness of nMAbs by Risk of Severe COVID-19 From the Disease Risk Score Model on 14- and 30-Day Outcomes **eTable 15.** nMAb Effectiveness by Variant Epoch **eTable 16.** nMAb Treatment Effectiveness by Product and Variant Epoch **eTable 17.** nMAb Effectiveness by Variant Sequence **eTable 18.** nMAb Treatment Effectiveness by Product and Variant Sequence: No WHO Equivalent, Epsilon, Alpha, and Other (eg, Mu and Iota) **eTable 19.** nMAb Treatment Effectiveness by Product and Variant Sequence: Delta 21AI, Delta 21J, and Omicron BA.1 **eAppendix.** Sensitivity Analyses **eTable 20.** Mean and Maximum Absolute SMD and KS Test Distance Comparing the Covariate Balance by Variant Epoch Using Single Propensity Model **eTable 21.** Mean and Maximum Absolute SMD and KS Test Distance Comparing the Covariate Balance by Variant Epoch Using Variant Epoch–Specific Propensity Models **eTable 22.** Effectiveness Results Using Propensity Models Developed Within Each Variant Epoch **eTable 23.** Effectiveness Results Using Exact Matching Procedure With Set 1 Matching Variables **eTable 24.** Effectiveness Results Using Exact Matching Procedure With Set 2 Matching Variables **eTable 25.** Subclassification of Immunocompromised States **eTable 26.** Effectiveness Results Using Propensity Models Developed Within Each Variant Epoch and Immunocompromised Subclasses **eTable 27.** Effectiveness Results Using Exact Matching Procedure With Set 1 Matching Variables, Substituting Immunocompromised Status With Immunocompromised Subclass **eTable 28.** Effectiveness Results Using Exact Matching Procedure With Set 2 Matching Variables, Substituting Immunocompromised Status with Immunocompromised Subclass **eTable 29.** Overall nMAb Treatment Effectiveness Testing the Influence of Immortal Time Bias
eReferences

Supplement 2.
Data Sharing Statement
