# Supplementary material for: Neutralizing Monoclonal Antibody Use and COVID-19 Infection Outcomes
Source: JAMA Netw Open. 2023 Apr 24;6(4):e239694. doi: 10.1001/jamanetworkopen.2023.9694 (PMC10126875; doi:10.1001/jamanetworkopen.2023.9694)
Supplement: Supplement 1. — eMethods. Data Model, Modeling Variable Definitions, Genomic Sequence Methodology, Missing Data Imputation, Propensity Model, and Disease Risk Score eTable 1. Thresholds for Genomic Data Quality eTable 2. Genomics Pipeline Steps in Order of Execution With Data Quality Check Outputs Identified eTable 3. Comparison of Demographic and Clinical Characteristics Between Patients With a Variant Call (n = 13 703) and Entire Study Population (n = 167 183) eFigure 1. Variable Groupings Used in Predictive Mean Matching, Propensity Score, and Disease Risk Score Models eTable 4. Hyperparameter Options Implemented for the Logistic Regression Grid Search eTable 5. Hyperparameter Options Implemented for the GBT Grid Search eTable 6. Hyperparameter Options Implemented for the RF Grid Search eFigure 2. Covariate Balance Plots Using Stabilized Weights eTable 7. Propensity Score Model Statistics eTable 8. Hyperparameter Options Implemented for the Logistic Regression Grid Search eTable 9. Metrics Used to Evaluate the Logistic Regression Models eFigure 3. Number of Patients by Treatment Category and Index Date Year and Month eFigure 4. nMAb Products Administered Through Time eTable 10. Percent of Nontreated and Treated Patients Considered Immunocompromised by Index Data Year and Month eTable 11. WHO Variant Classifications From Subset of Patients With Genome Sequences (n = 13 703) by Treatment Group eFigure 5. Distribution of WHO Variant Call From Genomic Sequences by Index Date Year and Month eTable 12. Treatment Effectiveness of nMAbs by COVID-19 Vaccination Status on 14- and 30-Day Outcomes eTable 13. Treatment Effectiveness of nMAbs by Immunocompromised Status on 14- and 30-Day Outcomes eTable 14. Treatment Effectiveness of nMAbs by Risk of Severe COVID-19 From the Disease Risk Score Model on 14- and 30-Day Outcomes eTable 15. nMAb Effectiveness by Variant Epoch eTable 16. nMAb Treatment Effectiveness by Product and Variant Epoch eTable 17. nMAb Effectiveness by Variant Sequence eTa [file jamanetwopen-e239694-s001.pdf]

Supplementary Online Content

Ambrose N, Amin A, Anderson B, et al. Neutralizing monoclonal antibody use and COVID-19 infection outcomes. *JAMA Netw Open*. 2023;6(4):e239694. doi:10.1001/jamanetworkopen.2023.9694

eMethods. Data Model, Modeling Variable Definitions, Genomic Sequence Methodology, Missing Data Imputation, Propensity Model, and Disease Risk Score

eTable 1. Thresholds for Genomic Data Quality

eTable 2. Genomics Pipeline Steps in Order of Execution With Data Quality Check Outputs Identified

eTable 3. Comparison of Demographic and Clinical Characteristics Between Patients With a Variant Call (n = 13 703) and Entire Study Population (n = 167 183)

eFigure 1. Variable Groupings Used in Predictive Mean Matching, Propensity Score, and Disease Risk Score Models

eTable 4. Hyperparameter Options Implemented for the Logistic Regression Grid Search

eTable 5. Hyperparameter Options Implemented for the GBT Grid Search

eTable 6. Hyperparameter Options Implemented for the RF Grid Search

eFigure 2. Covariate Balance Plots Using Stabilized Weights

eTable 7. Propensity Score Model Statistics

eTable 8. Hyperparameter Options Implemented for the Logistic Regression Grid Search

eTable 9. Metrics Used to Evaluate the Logistic Regression Models

eFigure 3. Number of Patients by Treatment Category and Index Date Year and Month

eFigure 4. nMAb Products Administered Through Time

eTable 10. Percent of Nontreated and Treated Patients Considered Immunocompromised by Index Data Year and Month

eTable 11. WHO Variant Classifications From Subset of Patients With Genome Sequences (n = 13 703) by Treatment Group

eFigure 5. Distribution of WHO Variant Call From Genomic Sequences by Index Date Year and Month

eTable 12. Treatment Effectiveness of nMAbs by COVID-19 Vaccination Status on 14- and 30-Day Outcomes

eTable 13. Treatment Effectiveness of nMAbs by Immunocompromised Status on 14- and 30-Day Outcomes

eTable 14. Treatment Effectiveness of nMAbs by Risk of Severe COVID-19 From the Disease Risk Score Model on 14- and 30-Day Outcomes

eTable 15. nMAb Effectiveness by Variant Epoch

eTable 16. nMAb Treatment Effectiveness by Product and Variant Epoch

eTable 17. nMAb Effectiveness by Variant Sequence

© 2023 Ambrose N et al. *JAMA Network Open*.

eTable 18. nMAb Treatment Effectiveness by Product and Variant Sequence: No WHO Equivalent, Epsilon, Alpha, and Other (eg, Mu and Iota)

eTable 19. nMAb Treatment Effectiveness by Product and Variant Sequence: Delta 21AI, Delta 21J, and Omicron BA.1

eAppendix. Sensitivity Analyses

eTable 20. Mean and Maximum Absolute SMD and KS Test Distance Comparing the Covariate Balance by Variant Epoch Using Single Propensity Model

eTable 21. Mean and Maximum Absolute SMD and KS Test Distance Comparing the Covariate Balance by Variant Epoch Using Variant Epoch–Specific Propensity Models

eTable 22. Effectiveness Results Using Propensity Models Developed Within Each Variant Epoch

eTable 23. Effectiveness Results Using Exact Matching Procedure With Set 1 Matching Variables

eTable 24. Effectiveness Results Using Exact Matching Procedure With Set 2 Matching Variables

eTable 25. Subclassification of Immunocompromised States

eTable 26. Effectiveness Results Using Propensity Models Developed Within Each Variant Epoch and Immunocompromised Subclasses

eTable 27. Effectiveness Results Using Exact Matching Procedure With Set 1 Matching Variables, Substituting Immunocompromised Status With Immunocompromised Subclass

eTable 28. Effectiveness Results Using Exact Matching Procedure With Set 2 Matching Variables, Substituting Immunocompromised Status with Immunocompromised Subclass

eTable 29. Overall nMAb Treatment Effectiveness Testing the Influence of Immortal Time Bias

eReferences

This supplemental material has been provided by the authors to give readers additional information about their work.

© 2023 Ambrose N et al. *JAMA Network Open*.

eMethods. Data Model, Modeling Variable Definitions, Genomic Sequence Methodology, Missing Data Imputation, Propensity Model, and Disease Risk Score

Data Model

Health systems transformed their data into a shared data model which was based on the Observational Medical Outcomes Partnership (OMOP) common data model, but adapted to address study goals and Safe-Harbor deidentification standards. To remove identifiable dates, the date of first positive COVID-19 test or referral date was used as the index date and all health record dates were calculated as relative dates from the index date. Certain subpopulations at greater risk of reidentification due to rare diseases or geography had their index date randomly shifted +/- 0 to 14 days. For each patient, the year and month of the index date was provided to allow for analysis through the pandemic time periods.

Modeling Variable Definitions

Patient demographics were collected at the index date and included age,birth sex, race, ethnicity, health insurance type, and marital status. Patients were categorized as a non-smoker, former smoker, or current smoker using the most recent smoking status or, if unavailable, the International Classification of Diseases, tenth revision, clinical modification (ICD-10-CM) conditions indicating nicotine smoking dependence.

Comorbid conditions in the two years prior to the index date were grouped according to Elixhauser and internally developed comorbidity groupings to provide indicator variables of comorbidity status.[1] Pregnancy status at time of the index date was determined using health system provided assertions, pregnancy-related ICD-10-CM condition codes, and human chorionic gonadotropin measurements in the 280 days prior to the index date. Medical technology dependence (e.g., respirator dependence, tracheostomy) was ascertained through conditions recorded in the 90 days prior to the index date.

Patients were considered obese if their most recent body mass index (BMI) was ≥ 30 kg/m^2 or they had a condition within the past two years that was in Elixhauser's obesity grouping. Medication records in the 90 days prior to the index date were assessed to determine which patients had a recent history of using immunosuppressants. Patients were considered immunosuppressed if they received immunosuppressant medications within 90 days or had an Elixhauser comorbidity in the acquired immune deficiency syndrome, other immune deficiency, or solid organ or blood stem cell transplantation groupings.

Patient COVID-19 vaccination statuses were categorized into patients with no record of COVID-19 vaccination, partially vaccinated, fully vaccinated, and fully vaccinated plus booster vaccine status categories, using COVID-19 vaccine records up to 14 days prior to their index date. The time from the patient's last vaccination administration to index date was categorized, and patients were grouped together if their last administration was within <14 days, 14 to 89 days, 90 to 179 days, and >=180 days from the index date.

To comply with Safe Harbor guidelines, health systems provided the 3-digit ZIP code of each patient's primary residence, which was mapped to a population density metric and the Area Deprivation Index (ADI).[2–4] Population densities from the 2019 American Community Survey by 5-digit ZIP code were weighted by the geographic areas to produce estimates by 3-digit ZIP code.

Past health care utilization was quantified by counting the number of healthcare visits in the previous two years from the index date. Previous evidence of 'clinical COVID', or evidence of a previous COVID-19 infection without a positive laboratory test, was assessed by reviewing conditions in the two years prior for an ICD-10-CM diagnosis of U07.1.

© 2023 Ambrose N et al. *JAMA Network Open*.

Adverse drug event (ADE) severity was assigned by investigators at each health system. A ‘mild’ ADE indicated vital sign monitoring, slowing the infusion rate, or supportive care but the treatment was completed. A ‘moderate’ ADE indicated monitoring per ‘mild’ ADE above, plus an on-site intervention (e.g., anti-histamines, steroids, anti-nausea medication). Treatment may or may not have been completed if a patient started experiencing a moderate ADE . A ‘severe’ ADE required escalation of care outside of the infusion site (e.g., urgent care, emergency department, hospital) for observation and/or management.

Genomic Sequence Methodology

COVID-19 Samples

SARS-CoV-2 genome sequences (14,771 in total) were obtained from the four health systems, based on samples collected from patients diagnosed with COVID-19 between July 2020 and January 2022. Samples were collected in conjunction with clinical practice related to COVID-19 diagnosis and treatment. The vast majority of samples were collected on day 0 (14,711). Samples selected for inclusion in this report met the following criteria: 1) were associated with a patient with clinical data included in the effectiveness study; 2) were taken on day 0 with each sample representing a distinct patient; and 3) met the established data quality requirements, provided in eTable 1. Therefore, the dataset is a non-random selection of sequences obtained from clinical samples and the distribution of demographics and co-morbidities likely differ from the full set of patient data. In addition, the dataset reflects an oversampling of the Delta variant within the time bounds of the study, reflecting the increased prevalence of the Delta variant within the viral population at that time and the high numbers of patients seeking medical care during the Delta wave.

Genome Sequencing Methods

Health systems performed sample storage, library creation, and sequencing according to their local protocols or those of their associated public health department. All health systems used paired-end sequencing protocols using primers based on the ARTIC tiling amplicon sequencing protocol (versions 3 and 4) for SARS-CoV-2 detection.[5] Additionally, one health system also used single-end sequencing protocols for some samples. Health systems used different sequencing platforms based on the availability within the respective clinical laboratories or public health department laboratories, but all were based on Next Generation Sequencing of single samples. FASTQ files were submitted by the health systems to the centralized registry for standardized data quality checks and processing.

Removal of PHI/PII

Potential protected health information (PHI) and personally identifiable information (PII) were removed from FASTQ files prior to acceptance into the deidentified registry using regular expressions to identify strings that might indicate PHI/PII within description lines and file names. Following the check for PHI/PII within the metadata and file headers, human sequence reads were removed from the FASTQ files, including the known human-specific short sequences used as internal controls in the ARTIC protocol, using the National Center for Biotechnology Information Sequence Read Archive human read removal tool.[6]

Data Quality Checking

Once verified to be free of PHI, PII, and human sequence reads, the cleaned FASTQ files were moved into the registry as raw data. FASTQ files were processed through the Data Quality (DQ) pipeline, which consists of nine checks that ensured all sample sequences met the standards agreed upon for this observational study (eTable 1).

© 2023 Ambrose N et al. *JAMA Network Open*.

A threshold for acceptance was set for each DQ check to account for uncontrolled variation across health systems in library creation and sequencing protocols and to allow comparison across health systems. Thresholds were based on health system capability limits and published standards, and fine-tuned using distribution graphs of data quality check results from submitted sequences.

The first four processes were *critical checks* required for a sample to pass from acceptance to analysis. The critical checks consisted of consensus sequence length, missing nucleotide data, base calling quality (Phred Q30), and coverage depth consistency.

The remaining five *non-critical checks* focused on virus-specific alignment, expected SARS CoV-2 genome GC content, RT-qPCR cycle thresholds, and two variant call quality checks. These checks were used to flag sequences with quality considerations and to measure the quality and confidence of nucleotide variant mutation calls. Analyses of nucleotide variants excluded variant calls that failed either of the two variant calling checks.

eTable 1. Thresholds for Genomic Data Quality

		#	Rule Type	Pipeline Phase	DQ Check Conditions
Sample QC	Critical Checks	1	Sequence Length	Consensus generation	Ensure that consensus sequence length is >= 27k bp
		2	Missing data	Consensus generation	Ensure that <= 5000 nucleotides are “no”
		3	Q30 rate	Sequence QC	Ensure that rate of base calls with quality >= Phred Q30 is >=80%
		4	Genome coverage	Alignment QC	Ensure that percentage of genome covered >10x is >=90% OR >=80% if checks 1, 2, and 3 are all PASS
		5	Proportion of reads aligned	Alignment QC	Ensure that >=75% of reads are aligned to the viral genome
		6	Average GC content	Sequence QC	Ensure that average GC content is 33 – 43%
		7	Clinical sample screening	Preanalytical	Ensure that RT-PCR cycle threshold is <=28
Variant QC		8	Variant call coverage	Variant calling	Ensure that variant calls are supported by 10x coverage
		9	Low-frequency variant calls	Variant calling	Ensure that number of low frequency variants (variants supported by <=70% of reads) is <=3

Genomics Data Processing Pipeline

Genome sequences were processed through the pipeline listed in eTable 2. Each pipeline step was distributed across several executor nodes, with executors being orchestrated by a driver node; outputs from each executor were organized into a central output dataset. Each step in the genomics pipeline is described below in terms of inputs, outputs, processing tools used, and, where relevant, parameter settings.

© 2023 Ambrose N et al. *JAMA Network Open*.

eTable 2. Genomics Pipeline Steps in Order of Execution With Data Quality Check Outputs Identified

Step	Tool	Parameters	Inputs	Outputs
**Pre-processing**	fastp	• Phred quality ≥ 30<br>• Percent of bases allowed to be unqualified ≤ 20<br>• Defaults used for all other parameters	• Forward and reverse read FASTQ files. Single FASTQ files in the case of single end reads	• Paired forward/reverse preprocessed FASTQ files<br>• Intermediate JSON QC file<br>• Used in QC #3 and #6
**Alignment**	minimap2	• Aligned to the reference SARS-CoV-2 genome by 20M query bases for each query sequence<br>• Used presets for short read alignment	• Paired forward and reverse FASTQ files. Single FASTQ files in the case of single end reads	• Aligned SAM files<br>• Intermediate SAMTOOLS stats file<br>• Used in QC #5
**BAM file creation**	SAMTOOLS view and SAMTOOLS sort	• No parameters required	• Aligned SAM files	• Sorted BAM files
**Primer trimming**	iVar trim	• Retain minimum length of read of 30 after trimming<br>• Minimum quality threshold of 30 for the sliding window to pass<br>• Include reads with no primers	• Sorted BAM files<br>• BED file with ARTIC PCR primer sequences	• Primer-trimmed BAM files
**Quality metrics**	qualimap bamqc	• Default parameter values	• Primer-trimmed BAM files	• Quality metrics on genome coverage and alignment<br>• Used in QC #4
**Assembly**	SAMTOOLS mpileup	• Retain anomalous base pairs<br>• Maximum file depth of 8000 to accommodate executor memory limits<br>• Minimum base quality of 0 (zero) as it is handled in a following step<br>• Base quality alignment disabled	• Primer-trimmed BAM files<br>• Reference SARS-CoV-2 reference genome	• Alignment pileup files
**Variant mutation calling**	iVar variants	• Minimum allele frequency of 0.75<br>• Minimum quality threshold of 30<br>• Minimum depth of coverage of 10	• Alignment pileup files	• VCFs<br>• Used in QC #8 and #9
**Annotation**	SnpEff	• No parameters required	• VCFs<br>• SnpEff Prediction Database<br>• Reference SARS-CoV-2 genome NC_045512.2	• Tables of specific genomic features for each sample (e.g., presence or absence of specific escape mutations for specific nMAbs)
**Consensus sequence**	iVar consensus	• Minimum allele frequency of 0.75<br>• Minimum quality threshold of 30<br>• Minimum depth of coverage of 10	• Alignment pileup files from assembly step	• Consensus FASTA files

© 2023 Ambrose N et al. *JAMA Network Open*.

Step	Tool	Parameters	Inputs	Outputs
**Lineage calling**	pangolin	• pangoLEARN model from 3/22/2022	• Consensus FASTA files	• Lineage calls
**Quality metrics from consensus sequences**	QUAST	• No parameters required	• Consensus FASTA files	• Quality metrics on consensus sequences<br>• Used in QC #1 and #2
**Results aggregation**	qc_aggregator	• No parameters required	• Checked results from quality metrics step using picard tool<br>• Checked results from quality metrics step using QUAST tool<br>• Checked results from pre-processing step<br>• Checked results from alignment step<br>• Checked results from variant mutation calling step	• Aggregated table with schema<br>• Aggregated table on variants with schema

Pre-processing: Paired forward and reverse read FASTQ files were input to *fastp* to produce paired forward/reverse preprocessed FASTQ files (in the case of single end reads, single FASTQ files were input to produce single preprocessed FASTQ files).[7] Default parameters for the *fastp* tool were used with the following exceptions: phred quality was required to be greater than or equal to 30, and percent of bases allowed to be unqualified less than or equal to 20.

Alignment: The pre-processed paired forward and reverse FASTQ files, or individual FASTQ files in the case of single end reads, were aligned to the reference SARS-CoV-2 genome[8] using minimap2[9] sequence aligner, specifying 20M query bases for each query sequence, to produce a SAM file output. Presets for short read alignment were used.

BAM file creation: The alignment output SAM file was transformed using *SAMTOOLS*[10] to generate a sorted BAM file.

Primer trimming: Sorted BAM files and a BED file containing ARTIC PCR primer sequences[11] were used as input to the *iVar trim* tool[12] to soft clip primer reads from the aligned sequence. Parameters were set to specify a minimum length of read of 30 to retain after trimming, a minimum quality threshold of 30 for the sliding window to pass, and to include reads with no primers.

Quality metrics on genome coverage: The primer-trimmed BAM files were input to the *QualiMap bamqc* tool[13] to collect quality metrics for genome coverage. In this step, default parameters were used (e.g., number of reads analyzed in a chunk was left at the default value of 1000).

© 2023 Ambrose N et al. *JAMA Network Open*.

Assembly: The primer-trimmed BAM file, along with the reference SARS-CoV-2 genome, were used to produce alignment pileup files and variant call files (VCF). The *SAMTOOLS* program was used to create the alignment pileup file, with specified parameters to retain anomalous base pairs, a maximum file depth of 8000 to accommodate executor memory limits, and with base quality alignment disabled as this is handled in a subsequent step.[14]

Variant mutation calling: The alignment pileup file was processed using the *iVar variants* tool to generate VCFs, with specification of a minimum allele frequency of 0.75, a minimum quality threshold of 30, and a minimum depth of 10 to call consensus. The native tab-separated-value output of *iVar trim* was converted to the standard VCF format.[15]

Annotation: VCFs were annotated using the *SnpEff annotation* and *functional effect prediction* tool,[16] along with the SNP prediction database for SARS-CoV-2.

Consensus sequence: The alignment pileup files were used to create consensus FASTA files using the *iVar consensus* command. Specified parameters included a minimum allele frequency of 0.75, 30 as the minimum quality threshold to count a base, and a minimum depth of coverage of 10 to call consensus.

Lineage calling: Lineage-calling based on the consensus FASTA files was accomplished using the *pangolin* tool,[17] specifying the *pangoLEARN* model from March 22, 2022.

Quality metrics on consensus FASTAs: Using the QUAST tool with default parameters, quality metrics were derived based on the FASTA consensus sequences.

Quality results aggregation: Quality statistics on each sample, output from fastp, QualiMap, iVar, and QUAST, were aggregated for further analysis. Quality statistics on SNPs and other sequence variants were also aggregated for further analysis.

Ambiguous base calls, deletions, gaps: Once data was passed through the pipeline, further analysis was conducted to identify the genomic position of all ambiguous base calls, deletions, and sequence gaps in each sample. Sample consensus FASTA files and the reference SARS-CoV-2 genome FASTA were processed using the mummer4 system. The nucmer script was run to align the consensus sample FASTA and the reference genome, followed by the show-aligns script to create a simple text-based representation of the alignment. Each of these commands was run with default parameter values. The text-based alignment file was parsed to determine the genomic position (relative to the reference genome) of each sample ambiguous base call (including any of the following IUPAC[18] codes: R, Y, S, W, K, M, B, D, H, V, or N), deletion (represented as '.' in the alignment), and gap (defined as any genomic position in the reference genome occurring between two separate aligned segments from the sample consensus FASTA sequence produced by mummer4).

Lineage Assignments and Nucleotide Variant Analyses

Samples were classified using multiple types of lineage designations that vary in granularity: PANGO lineage, a widely used taxonomic nomenclature for SARS-CoV-2;[17] WHO Variants of Concern or Variants Being Monitored;[19,20] and Nextstrain clades within Delta. PANGO lineages were assigned using the *pangolin* tool. WHO variants were assigned based on the PANGO lineage calls by mapping PANGO lineage prefixes to WHO variants.[19] Assigned WHO variants within the study included Alpha, Beta, Gamma, Epsilon, Eta, Kappa, Iota, Mu, Zeta, Lambda, Theta, unnamed B.1.617.3, Delta, and Omicron, as well as a class for lineages not within these, called "no_WHO_variant_equivalent". "no_WHO_variant_equivalent" includes early Wuhan-like lineages prior to Alpha designation. Delta samples were assigned to two Nextstrain clade groups, 21J and 21AI, a designation which combines NextStrain clade 21A within its derivative clade 21I. Delta samples were assigned to these based on the detection of nucleotide variants that are characteristic of 21J but not 21A or 21I. The list of nucleotide

© 2023 Ambrose N et al. *JAMA Network Open*.

variants characteristic of 21J was obtained from Covariants.org[21] and included G4181T, C10029T, A11201G, A11332G, G9053T, C27874T, C19220T, C8986T, and G28916T. Delta samples with at least five of these nine 21J nucleotide variants were assigned to 21J and Delta strains with fewer than five of these nucleotide variants were assigned to 21AI.

The decision to classify Delta strains into the 21J and 21AI groups was based on an independent analysis of clusters within the Delta samples. For this, nucleotide variant data was analyzed with k-means clustering using Jaccard distances, which were calculated with the R package *locstra*.[22] To avoid spurious clusters due to missing data, the analysis included only nucleotide variants at genomic positions at which no more than 2% of the Delta samples were missing data due to sequencing gaps or ambiguous base calls. To characterize and illustrate clustering across samples from all WHO variants, a principal component analysis (PCA) was performed with Jaccard distances with nucleotide variants at positions with 2% or less missing data.

Annotations from *SnpEff* were used to identify putative effects of single nucleotide polymorphisms (SNPs) and insertions and deletions (indels) on gene function. The first *SnpEff* annotation for each nucleotide variant was used in these analyses. All calculations of frequencies of nucleotide variants excluded samples with ambiguous calls or gaps at the corresponding genomic positions.

Comparison of Patients with Variant Call to Entire Study Population

eTable 3. Comparison of Demographic and Clinical Characteristics Between Patients With a Variant Call (n = 13 703) and Entire Study Population (n = 167 183)

Characteristic	Patients with a Variant Call, No (%)	Entire Study Population, No (%)
**Treated with nMAbs**	4,263 (31.1)	25,241 (15.1)
**Age in years – mean (standard deviation)**	47.8 (18.1)	47.0 (18.5)
**Female**	7,773 (56.7)	95,669 (57.2)
Not specified	0 (0.0)	5 (<0.1)
**Race**		
American Indian or Alaska Native	115 (0.8)	1,391 (0.8)
Asian	328 (2.4)	4,290 (2.6)
Black or African American	846 (6.2)	10,746 (6.4)
Native Hawaiian or Other Pacific Islander	121 (0.9)	1,800 (1.1)
White	11,405 (83.2)	139,379 (83.4)
Other	655 (4.8)	6,402 (3.8)
Not specified	233 (1.7)	3,175 (1.9)
**Hispanic or Latino**	1,973 (14.4)	23,615 (14.1)
Not specified	366 (2.7)	4,668 (2.8)
**COVID-19 vaccination status**		
No record of COVID-19 vaccination	7,976 (58.2)	104,983 (62.8)
Partially vaccinated	337 (2.5)	4,403 (2.6)
Fully vaccinated	4,509 (32.9)	44,284 (26.5)
Fully vaccinated and boosted	881 (6.4)	13,513 (8.1)
**Variant epoch**		
pre-Delta (2020-11 to 2021-06)	2,754 (20.1)	62,443 (37.4)
Delta (2021-07 to 2021-11)	7,804 (57.0)	44,409 (26.6)

© 2023 Ambrose N et al. *JAMA Network Open*.

Characteristic	Patients with a Variant Call, No (%)	Entire Study Population, No (%)
Delta/Omicron BA.1 (2021-12)	1,141 (8.3)	17,921 (10.7)
Omicron BA. 1 (2022-01)	2,004 (14.6)	42,410 (25.4)
**Smoker**	901 (6.6)	10691 (6.4)
**Pregnant**	418 (3.1)	5143 (3.1)
**Immunocompromised**	1342 (9.8)	15021 (9.0)
**Comorbidities/coexisting conditions**		
Obesity	6190 (45.2)	70908 (42.4)
Hypertension	2947 (21.5)	35043 (21.0)
Diabetes mellitus	1490 (10.9)	17584 (10.5)
Chronic pulmonary disease	1271 (9.3)	15545 (9.3)
Thrombotic or hematological disorders	996 (7.3)	11266 (6.7)
Hypothyroidism	974 (7.1)	11311 (6.8)
Malignancy	659 (4.8)	7672 (4.6)
Chronic kidney disease	611 (4.5)	7100 (4.2)
Peripheral vascular disease	654 (4.8)	7431 (4.4)
Heart failure	393 (2.9)	4673 (2.8)
Neurological disorders or paralysis	542 (4.0)	6798 (4.1)
Chronic liver disease	468 (3.4)	5824 (3.5)
Valvular heart disease	245 (1.8)	3240 (1.9)

Missing Data Imputation

For this study, imputation methods implemented were based on codes in the R *mice* package.[23] Predictive mean matching (PMM) was used as the primary imputation model in the Multiple Imputation by Chained Equations (MICE) algorithm. The PMM approach ensures that imputed values retain the same characteristics as the complete portion of the dataset. The MICE algorithm uses Monte Carlo Markov Chain (MCMC) methods to generate candidate values to replace missing covariates. This MCMC method begins from a random initial value and then generates a dependent parameter sequence by sampling the full conditional probability distributions for each parameter of a model for the missing data. Parameter samples drawn from the chains before the sampler converges are discarded as burn-in. Post-burnin parameter samples are used to generate a predicted value for the missing variable. Donor records (d=5) nearest to the predicted value are randomly sampled. The missing covariate is replaced with the donor value. Once all missing values for the covariate are imputed with this posterior parameter sample, the MICE algorithm moves to the next covariate and starts again. The MICE algorithm terminates after M=20 iterations through each covariate with one or more missing observations.

The influence of imputation-modeling methods was evaluated by comparing imputed results from the PMM method to results using Bayesian stochastic regression. No substantive differences were identified using the Bayesian stochastic regression approach compared to PMM. This indicates that statistical inferences derived from the imputed data were not substantively impacted by the choice of imputation model.

Variables used in PMM matched variables used in the subsequent propensity score and disease risk score models (eFigure 1), with the exception that the imputation models used the outcome variables as predictors in the imputation step.[24] Multiple imputation was carried out for each health system independently. Variables with more than 30% of their values missing were not imputed and were

© 2023 Ambrose N et al. *JAMA Network Open*.

eliminated from consideration in downstream analysis. This missing values threshold excluded the following variables from further consideration:

- Pneumococcal polysaccharide vaccine (Pneumovax 23) status
- Pneumococcal conjugate vaccine (Prevnar 13) status
- Influenza vaccine status and date
- Index date vital signs (e.g., oxygen saturation percentage, respiratory rate, blood pressure, heart rate, temperature).
- COVID-19 symptoms and symptom onset date

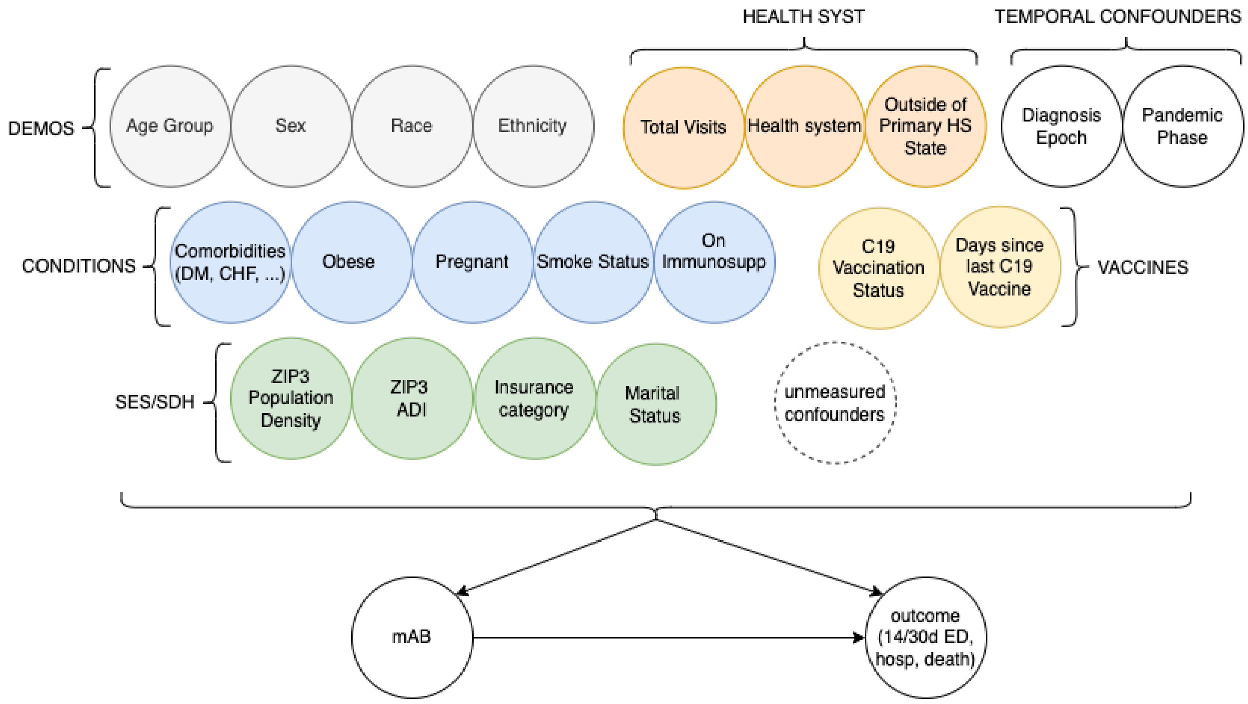

eFigure 1. Variable Groupings Used in Predictive Mean Matching, Propensity Score, and Disease Risk Score Models

Propensity Model

The propensity score (PS) in this study is defined as the probability of receiving nMAbs conditional on a set of observed covariates. The PS was used to compute weights in the marginal structural models (MSM). The variables included in the PS model are shown in eFigure 1**Error! Reference source not found.**. These variables were identified as measurable confounders by clinical experts, including feedback from participating health systems.

Propensity scores were estimated using logistic regression, gradient boosted trees (GBT), and random forest (RF). The estimation of multiple PS models was *a priori* specified in the statistical analysis plan. The PS models were implemented with Python's scikit-learn API.[25] A hyperparameter grid search was then performed on each model for model selection using hyperparameter options described in eTable 4 for logistic regression, eTable 5 for GBT, and eTable 6 for RF. The parameter "C" denotes the inverse of regularization strength. Parameters not specified in this description or in the tables used the default parameter values. All possible combinations of the hyperparameters in each row were implemented.

© 2023 Ambrose N et al. *JAMA Network Open*.

All logistic regression models had a maximum of 500 iterations, with a stopping tolerance of 1×10^{-4}. Regularization was applied with both the ℓ1 ("Lasso") and ℓ2 ("Ridge") penalties. Every GBT model had a maximum of 500 estimators, with an early stopping tolerance of 1×10^{-3}, patience of 25, and validation fraction of 0.2. The RF models used out-of-bag samples to estimate the generalization score during training.

eTable 4. Hyperparameter Options Implemented for the Logistic Regression Grid Search

Grid	Penalty	C	Solver	Class Weight
LR Grid #1	ℓ1, ℓ2	1, 10	saga, liblinear	None, balanced
LR Grid #2	ℓ2	1	lbfgs, newton-cg	None, balanced

eTable 5. Hyperparameter Options Implemented for the GBT Grid Search

Grid	Learning Rate	Subsample	Max Features	Max Tree Depth
GBT Grid	0.1, 0.01	50%, 100%	50%, 100%	5, 10, 20

eTable 6. Hyperparameter Options Implemented for the RF Grid Search

Grid	Max Tree Depth	Minimum Samples per Leaf	Class Weight	Max Subsamples	Number of Trees
RF Grid	5, 10, 20	1, 10	None, balanced subsample	25%, 50%	50, 100, 250, 500

Data was preprocessed as necessary (e.g., one-hot encoding and normalization). Logistic regression, GBT, and RF models were implemented and evaluated within each imputation group. Although the loss function used for PS model training optimizes the classification accuracy, model selection was determined by the optimal covariate balance.

The standardized mean difference (SMD) and Kolmogorov Smirnov 2-sample (KS) tests were used to assess covariate balance. Belitser et al. (2011) [26] recommend a threshold of 0.1 for assessing sufficient balance of absolute SMDs. Computation of SMDs and KS test statistics was performed using the R package *cobalt*.[27] Mean and max statistics were aggregated across covariates to evaluate the comparative balance of each model. The model that minimized these aggregate statistics was selected for use in the effectiveness analysis. The resulting model was a logistic regression with ℓ1 regularization and an inverse regularization strength of 0.1, balanced class weights, using the SAGA solver. With this model, all covariates had absolute SMDs of less than 0.1 between treated and non-treated patients, as shown in eFigure 2.

© 2023 Ambrose N et al. *JAMA Network Open*.

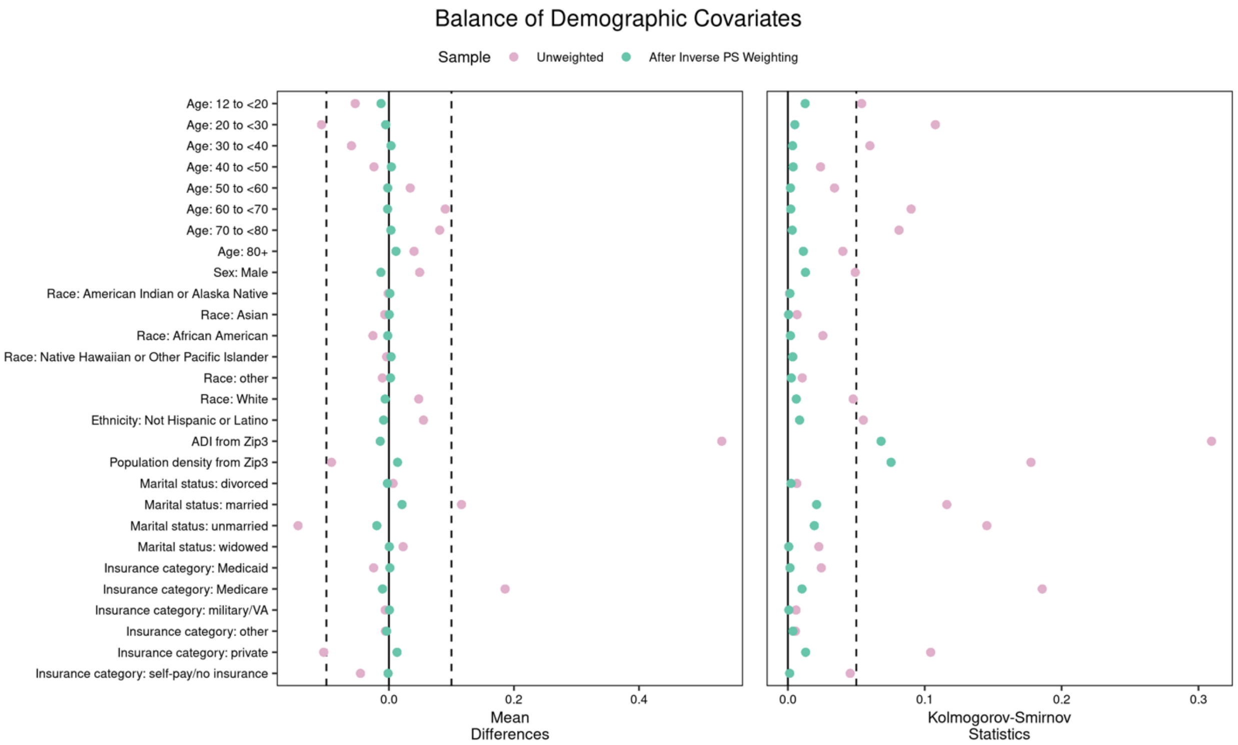

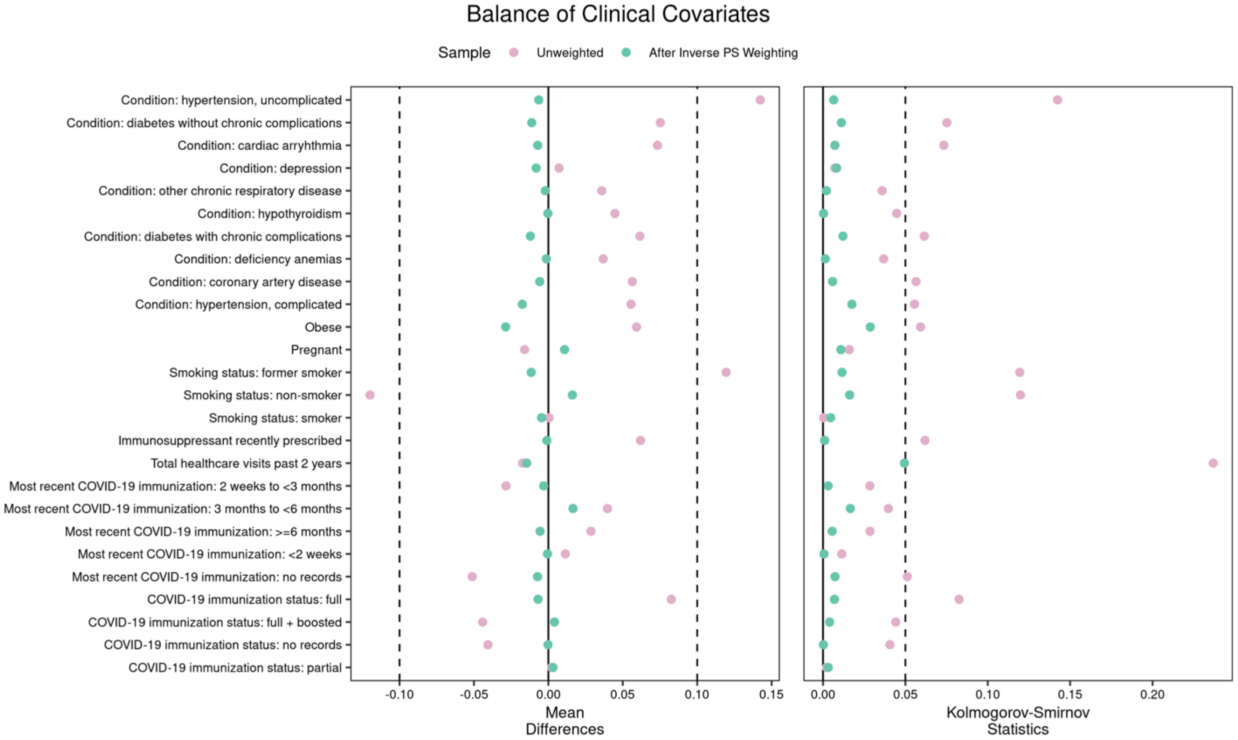

© 2023 Ambrose N et al. *JAMA Network Open*.

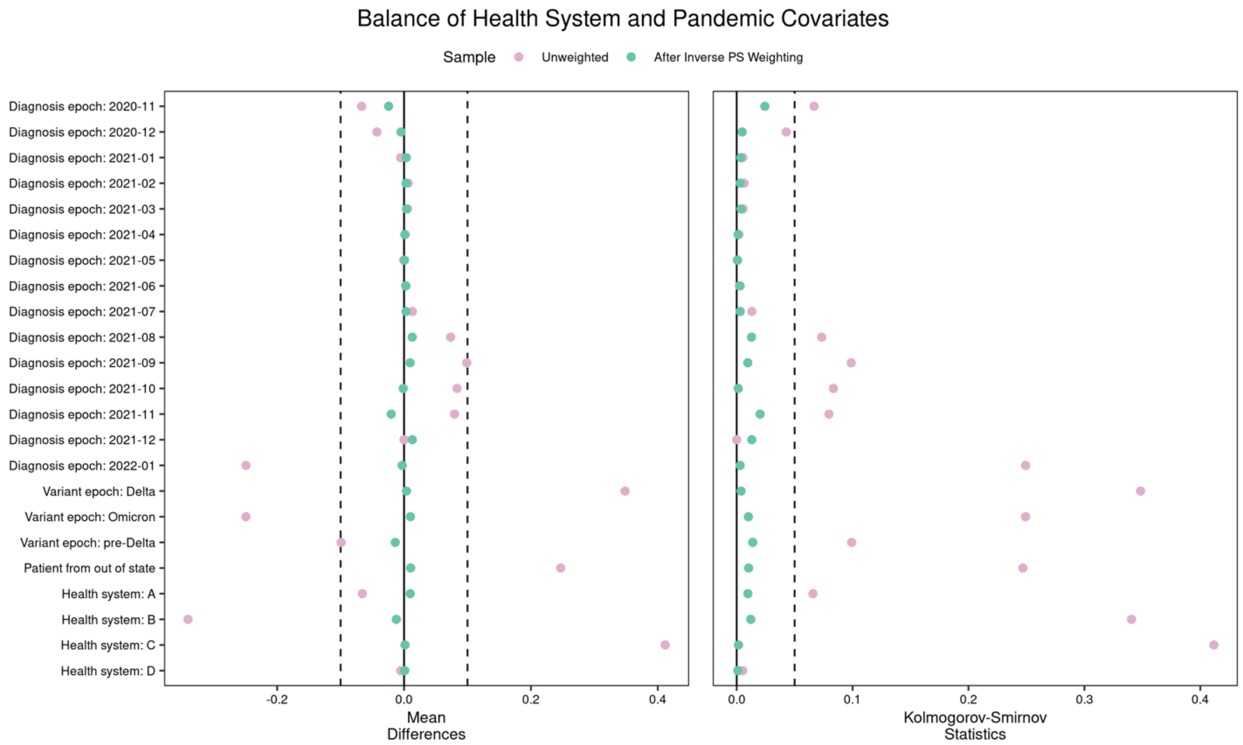

eFigure 2. Covariate Balance Plots Using Stabilized Weights.

The relative importance of the variables in the PS model were determined using the absolute value of coefficients (eTable 7). The coefficients reported are the average across the ten imputation groups.

eTable 7. Propensity Score Model Statistics. Importance Rank Determined by the Absolute Value of the Coefficients

Variable	Coefficient	Standard Error	p-value	95% Confidence Interval	Importance Rank
**Intercept**	-0.3934	0.0490	<0.0001	(-0.4894, -0.2975)	16
**Age in years, baseline = 80+**					
10 to <20	-1.4445	0.0548	<0.0001	(-1.5520, -1.3370)	3
20 to <30	-1.0222	0.0454	<0.0001	(-1.1113, -0.9331)	5
30 to <40	-0.6114	0.0427	<0.0001	(-0.6951, -0.5278)	11
40 to <50	-0.3618	0.0417	<0.0001	(-0.4436, -0.2800)	18
50 to <60	-0.1632	0.0413	0.0001	(-0.2441, -0.0824)	35
60 to <70	0	0.0386	1.0000	(-0.0756, 0.0756)	65
70 to <80	0.0597	0.0380	0.1166	(-0.0149, 0.1343)	52
**Female birth sex, baseline = male**	-0.1040	0.0137	<0.0001	(-0.1309, -0.0771)	44
**Comorbidities**					
Acquired heart disease	0	0.1181	1.0000	(-0.2315, 0.2315)	65
Acquired immune deficiency syndrome	0	0.1700	1.0000	(-0.3332, 0.3332)	65
Alcohol abuse	0	0.0608	1.0000	(-0.1192, 0.1192)	65

© 2023 Ambrose N et al. *JAMA Network Open*.

Variable	Coefficient	Standard Error	p-value	95% Confidence Interval	Importance Rank
Arthropathies	0.5373	0.0386	<0.0001	(0.4618, 0.6129)	13
Asthma	0	0.0963	1.0000	(-0.1888, 0.1888)	65
Coronary artery disease	0.2552	0.0354	<0.0001	(0.1858, 0.3247)	21
Cardiac arrhythmia	0.0769	0.0305	0.0118	(0.0170, 0.1367)	47
Cardiomyopathy	0	0.0768	1.0000	(-0.1506, 0.1506)	65
Cerebrovascular disease	0	0.0461	1.0000	(-0.0904, 0.0904)	65
Chronic blood loss anemia	0	0.0925	1.0000	(-0.1814, 0.1814)	65
Coagulopathy	0	0.0581	1.0000	(-0.1139, 0.1139)	65
Congenital heart disease	0	0.0872	1.0000	(-0.1709, 0.1709)	65
Chronic obstructive pulmonary disease	0	0.0900	1.0000	(-0.1763, 0.1763)	65
Cystic fibrosis	0	0.4604	1.0000	(-0.9023, 0.9023)	65
Deficiency anemias	0	0.0316	1.0000	(-0.0620, 0.0620)	65
Dementia	0	0.0847	1.0000	(-0.1661, 0.1661)	65
Depression	-0.0036	0.0225	0.8720	(-0.0477, 0.0404)	62
Diabetes with chronic complications	0.1568	0.0325	<0.0001	(0.0931, 0.2204)	37
Diabetes without chronic complications	0.3808	0.0266	<0.0001	(0.3287, 0.4328)	17
Down syndrome	0	0.3498	1.0000	(-0.6856, 0.6856)	65
Drug abuse	0	0.0565	1.0000	(-0.1107, 0.1107)	65
Heart failure	0	0.0476	1.0000	(-0.0933, 0.0933)	65
Hypercoagulable state	0	0.0622	1.0000	(-0.1220, 0.1220)	65
Hypertension complicated	0	0.0472	1.0000	(-0.0924, 0.0924)	65
Hypertension uncomplicated	0.2137	0.0181	<0.0001	(0.1783, 0.2491)	25
Hypothyroidism	0.0299	0.0260	0.2508	(-0.0211, 0.0809)	59
Interstitial lung disease	0	0.1082	1.0000	(-0.2120, 0.2120)	65
Leukemia	0	0.1203	1.0000	(-0.2357, 0.2357)	65
Liver disease mild	0.0951	0.0365	0.0091	(0.0237, 0.1666)	45
Liver disease moderate to severe	0	0.1068	1.0000	(-0.2093, 0.2093)	65
Lymphoma	0.1138	0.0978	0.2443	(-0.0778, 0.3055)	42
Metastatic cancer	0	0.0861	1.0000	(-0.1688, 0.1688)	65
Neurodevelopmental disorders	0	0.3006	1.0000	(-0.5892, 0.5892)	65
Neurological disorders affecting movement	0	0.0533	1.0000	(-0.1044, 0.1044)	65
Other chronic respiratory disease	0.2316	0.0983	0.0184	(0.0390, 0.4242)	23
Other immune deficiency	0.8785	0.0840	<0.0001	(0.7138, 1.0433)	7
Other neurological disorders	0.0663	0.0590	0.2611	(-0.0493, 0.1818)	50
Other thyroid disorders	0	0.0459	1.0000	(-0.0900, 0.0900)	65
Pancreatitis	0	0.0937	1.0000	(-0.1836, 0.1836)	65
Paralysis	0	0.1052	1.0000	(-0.2063, 0.2063)	65

© 2023 Ambrose N et al. *JAMA Network Open*.

Variable	Coefficient	Standard Error	p-value	95% Confidence Interval	Importance Rank
Peptic ulcer disease	0	0.0806	1.0000	(-0.1579, 0.1579)	65
Peripheral vascular disease	0.0175	0.0333	0.5995	(-0.0477, 0.0827)	61
Psychoses	0	0.0335	1.0000	(-0.0657, 0.0657)	65
Pulmonary circulation disorders	-0.0297	0.0381	0.4365	(-0.1044, 0.0451)	60
Renal failure moderate	0.1895	0.0373	<0.0001	(0.1165, 0.2626)	29
Renal failure severe	0	0.0717	1.0000	(-0.1406, 0.1406)	65
Seizures and epilepsy	0	0.0573	1.0000	(-0.1123, 0.1123)	65
Sickle cell disease	0	0.3032	1.0000	(-0.5943, 0.5943)	65
Solid organ or blood stem cell transplantation	0.8655	0.0924	<0.0001	(0.6845, 1.0465)	8
Solid tumor without metastasis in situ	0	0.0905	1.0000	(-0.1774, 0.1774)	65
Solid tumor without metastasis malignant	0.1870	0.0367	<0.0001	(0.1151, 0.2589)	30
Thalassemia	0	0.2857	1.0000	(-0.5600, 0.5600)	65
Valvular disease	0.0477	0.0566	0.3989	(-0.0632, 0.1586)	56
Weight loss	0	0.0594	1.0000	(-0.1164, 0.1164)	65
**Index date year and month**					
2020-11	-1.5418	0.0288	<0.0001	(-1.5982, -1.4853)	2
2020-12	-0.6396	0.0206	<0.0001	(-0.6800, -0.5991)	9
2021-01	-0.2523	0.0211	<0.0001	(-0.2938, -0.2109)	22
2021-02	0	0.0308	1.0000	(-0.0605, 0.0605)	65
2021-03	0	0.0370	1.0000	(-0.0726, 0.0726)	65
2021-04	0	0.0410	1.0000	(-0.0803, 0.0803)	65
2021-05	0	0.0484	1.0000	(-0.0949, 0.0949)	65
2021-06	0.3976	0.0506	<0.0001	(0.2984, 0.4968)	15
2021-07	-0.3414	0.0301	<0.0001	(-0.4004, -0.2824)	19
2021-08	-0.0028	0.0215	0.8981	(-0.0449, 0.0394)	63
2021-09	0.1858	0.0211	<0.0001	(0.1445, 0.2271)	31
2021-10	0.1989	0.0233	<0.0001	(0.1532, 0.2446)	27
2021-11	0.1197	0.0231	<0.0001	(0.0743, 0.1650)	41
2021-12	0.1270	0.0148	<0.0001	(0.0980, 0.1559)	40
2022-01	-1.9157	0.0154	<0.0001	(-1.9457, -1.8856)	1
**Hispanic ethnicity, baseline = non-Hispanic**	0.0627	0.0201	0.0031	(0.0233, 0.1021)	51
**Health system**					
Health system A	1.2428	0.0262	<0.0001	(1.1916, 1.2941)	4
Health system B	0	0.0238	1.0000	(-0.0467, 0.0467)	65
Health system C	0	0.0497	1.0000	(-0.0973, 0.0973)	65
Health system D	-0.5713	0.0192	<0.0001	(-0.6090, -0.5337)	12

© 2023 Ambrose N et al. *JAMA Network Open*.

Variable	Coefficient	Standard Error	p-value	95% Confidence Interval	Importance Rank
**COVID-19 immunization status, baseline = no record of COVID-19 vaccination**					
Partially vaccinated	0	0.0771	1.0000	(-0.1510, 0.1510)	65
Fully vaccinated	-0.2181	0.0713	0.0022	(-0.3580, -0.0783)	24
Fully vaccinated + booster	-0.1515	0.0809	0.0611	(-0.3100, 0.0070)	38
**Prescribed immunosuppressant within 90 days prior to index date**	0.0844	0.0067	<0.0001	(0.0713, 0.0975)	46
**Insurance category, baseline = other**					
Medicaid	0	0.0595	1.0000	(-0.1167, 0.1167)	65
Medicare	0.4056	0.0591	<0.0001	(0.2899, 0.5214)	14
Military or Veterans Administration insurance	0	0.0813	1.0000	(-0.1594, 0.1594)	65
Private insurance	0.1822	0.0558	0.0011	(0.0728, 0.2916)	32
Self-pay/no insurance	-0.1588	0.0609	0.0092	(-0.2782, -0.0395)	36
**Marital status, baseline = unmarried**					
Divorced	0	0.0285	1.0000	(-0.0559, 0.0559)	65
Married	0.1672	0.0182	<0.0001	(0.1316, 0.2028)	34
Widowed	0.0493	0.0392	0.2110	(-0.0275, 0.1261)	54
**Time from most recent COVID-19 vaccination to index date**					
No record of COVID-19 vaccination	0	0.0454	1.0000	(-0.0891, 0.0891)	65
< 2 weeks	0	0.0334	1.0000	(-0.0654, 0.0654)	65
2 weeks to < 3 months	-0.1759	0.0390	<0.0001	(-0.2524, -0.0993)	33
3 months to < 6 months	0.0492	0.0347	0.1556	(-0.0187, 0.1172)	55
≥ 6 months	0	0.0334	1.0000	(-0.0655, 0.0655)	65
**Patient obese, baseline = 'no'**	0.1927	0.0135	<0.0001	(0.1661, 0.2192)	28
**Patient's home out of health system's primary state, baseline = 'no'**	0.6260	0.0250	<0.0001	(0.5770, 0.6750)	10
**Variant epoch**					
pre-Delta	0	0.0207	1.0000	(-0.0405, 0.0405)	64
Delta	0.9338	0.0190	<0.0001	(0.8967, 0.9710)	6
Omicron BA.1	0	0.0165	1.0000	(-0.0324, 0.0324)	65
**Patient pregnant, baseline = 'no'**	0.2085	0.0385	<0.0001	(0.1330, 0.2839)	26
**Race, baseline = 'other'**					
American Indian or Alaska Native	0	0.0775	1.0000	(-0.1518, 0.1518)	65
Asian	0	0.0524	1.0000	(-0.1026, 0.1026)	65
Black or African American	-0.1457	0.0442	0.0013	(-0.2324, -0.0590)	39
Native Hawaiian or Other Pacific Islander	0.1106	0.0714	0.1235	(-0.0293, 0.2505)	43
White	-0.0423	0.0345	0.2284	(-0.1099, 0.0253)	57

© 2023 Ambrose N et al. *JAMA Network Open*.

Variable	Coefficient	Standard Error	p-value	95% Confidence Interval	Importance Rank
**Patient's smoking status, baseline = non-smoker**					
Former smoker	-0.0323	0.0201	0.1086	(-0.0717, 0.0072)	58
Current smoker	-0.3069	0.0274	<0.0001	(-0.3606, -0.2531)	20
**Total number of healthcare visits in two years prior to index date**	0.0709	0.0084	<0.0001	(0.0544, 0.0873)	49
**Area deprivation index from three-digit ZIP code**	-0.0496	0.0107	<0.0001	(-0.0705, -0.0287)	53
**Population density from 3-digit ZIP code**	0.0737	0.0087	<0.0001	(0.0566, 0.0908)	48

Disease Risk Score

A patient's disease risk score (DRS) is the risk of the composite study outcome (30-day hospitalization or death) if the COVID-19 patient did not receive nMAb treatments. Development of the DRS model was guided by applicable research, clinical subject matter expertise, health system feedback, and best practices in machine learning. In this study, the DRS was used as an effect modifier for evaluating effectiveness.

A variety of modeling and analytic methods were explored while developing the DRS model, including logistic regression and various versions of decision trees. Logistic regression, RF, and GBT were evaluated for use in the DRS model. The highest ranking logistic regression model was used for this study because it was less likely to overtrain the model than the tree based methods, it was well-calibrated, and the results and coefficients were easily interpreted.

The features used in the DRS model were identical to those used in the PS model. Data was preprocessed as necessary (e.g., one-hot encoding and normalization). The DRS models were implemented with Python's scikit-learn application programming interface (API).[25] A hyperparameter grid search was then performed on the first imputation group for parameter selection using hyperparameter options for logistic regression described in eTable 8. The parameter "C" denotes the inverse of regularization strength. The regularization strength penalizes model complexity. Therefore, the higher the regularization strength (or the lower its inverse, or C), the simpler the model. Parameters not specified in this description or in the tables used the default parameter values. All possible combinations of the hyperparameters in each row were implemented.

All logistic regression models had a maximum of 500 iterations, with a stopping tolerance of 1×10^{-4}. Regularization was applied with both the ℓ1 ("Lasso") and ℓ2 ("Ridge") penalties. Elasticnet combines ℓ1 and ℓ2 penalties based on the ℓ1 Ratio (e.g., 0.25 ℓ1 Ratio means ℓ1 is applied at 25% and ℓ2 at 75%). Best parameters based on logistic regression grid search are highlighted in bold.

eTable 8. Hyperparameter Options Implemented for the Logistic Regression Grid Search

Grid	Penalty	C	Solver	Class Weight	Max iteration
LR Grid #1	ℓ1, **ℓ2**	0.001, 0.1, 1, **10**	**saga**, liblinear	**balanced**	**500**
LR Grid #2	ℓ2	0.001, 0.1, 1, 10	newton-cg	None	500, 1000
LR Grid #3	elasticnet	0.1, 1, 10	saga	balanced	500

The DRS was developed on 141,942 non-treated patients whose profiles were used to fit the model. The model was then applied to all 167,183 patients in the study regardless of nMAb treatment status. In

© 2023 Ambrose N et al. *JAMA Network Open*.

general, one would expect the model to perform better on data used in training (the non-treated patients). In order to quantify the bias of the model on the non-treated population, an experiment was performed where an “experimental” model was trained on a subset of patients. 80% of the non-treated patients were used for training and the remaining 20% of the non-treated patients were used to test the performance of the model. Results of the experiment based on several metrics are presented in eTable 9 including counts of True Negatives (TN), False Positives (FP), False Negatives (FN), and True Positives (TP) as well as scores for Positive Predicted Values (PPV), Recall, and Matthews Correlation Coefficient (MCC). TN is the number of patients with DRS less than 0.5 who did not experience the study outcome (hospitalization and/or death). FP is the number of patients with DRS of 0.5 or above who did not experience the study outcome. FN the number of patients with DRS less than 0.5 who experienced the study outcome. TP is the number patients with DRS of 0.5 or above who were admitted to the hospital or died. The PPV is the fraction of relevant instances (patients who experienced the study outcome) among the retrieved instances (all patients with DRS > 0.5), while Recall is the fraction of relevant instances (all patients experiencing the study outcome) that were retrieved (having DRS of 0.5 or higher). MCC, which is similar to Pearson correlation coefficient in its interpretation, produces high scores if the DRS obtained good results in all four confusion matrix metrics (TN, FP, FN, TP).

eTable 9. Metrics Used to Evaluate the Logistic Regression Models

Population	TN	FP	FN	TP	PPV	Recall	MCC	Total Patients
Non-treated population used in training model	83,410	25,665	831	3,647	0.12	0.81	0.26	113,553
Non-treated population not used in training model	20,898	6,372	270	849	0.11	0.76	0.23	28,389
Treated population	11,366	12,876	208	791	0.06	0.79	0.10	25,241
All	115,674	44,913	1,309	5,287	0.10	0.80	0.22	167,183

The experiment shows that DRS performs similarly on non-treated patients used to train the model and non-treated patients not used to train the model. The FP rate is higher for the treated population compared to the FP rate for the 20% of non-treated patients not used in model training. Since the model does not know if patients were treated, the treated false positives may be indicative of the effects of nMAbs in reducing the risk of bad outcomes. Therefore, treated false positive patients could have been true positive patients if the patients were not treated.

In this study, the DRS was ultimately used as an effect modifier for evaluating the effectiveness of nMAbs. While the false positive rate and recall rate were within acceptable bounds to use the DRS as an effect modifier, additional model tuning and refinement would be required to use the DRS as a predictive model for direct use in clinical applications. Any model used for clinical applications should also be validated in additional cohorts, such as data from other health systems outside the scope of this study that vary geographically and demographically.

© 2023 Ambrose N et al. *JAMA Network Open*.

eFigure 3. Number of Patients by Treatment Category and Index Date Year and Month

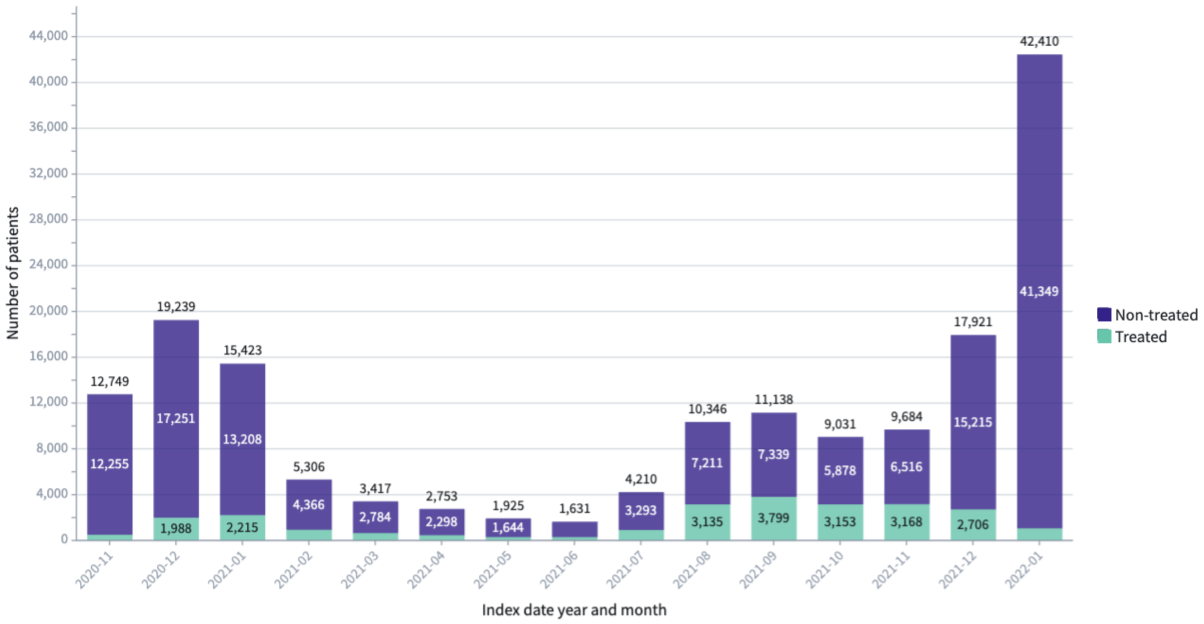

© 2023 Ambrose N et al. *JAMA Network Open*.

eFigure 4. nMAb Products Administered Through Time

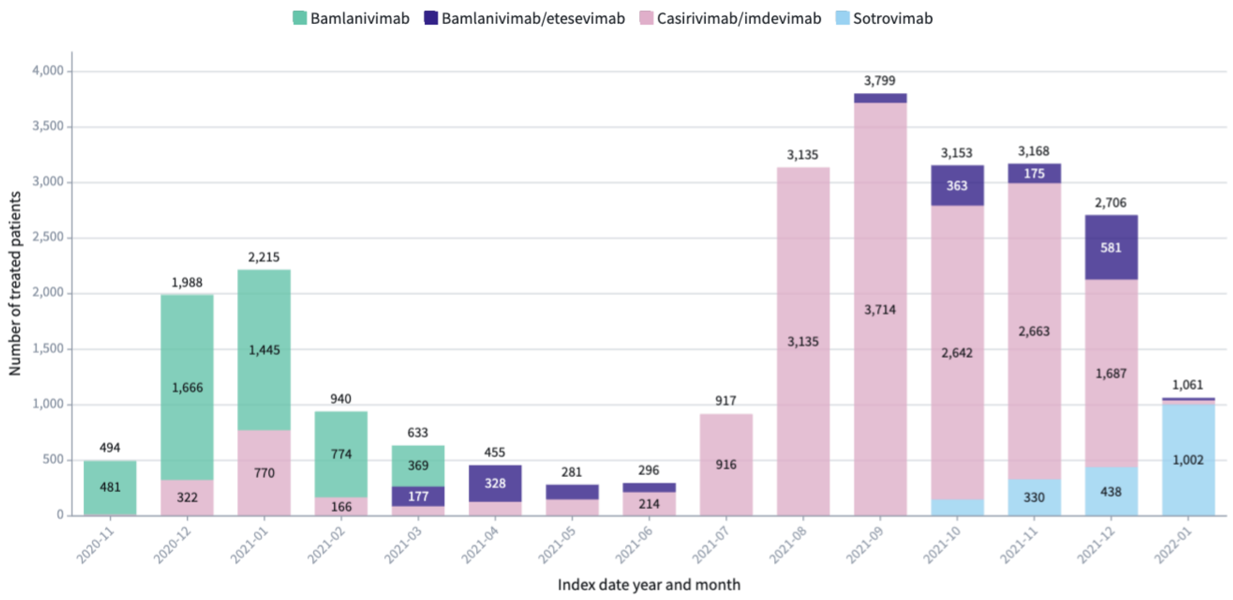

© 2023 Ambrose N et al. *JAMA Network Open*.

eTable 10. Percent of Nontreated and Treated Patients Considered Immunocompromised by Index Data Year and Month

Index date year and month	Non-treated considered immunocompromised, %	Treated considered immunocompromised, %
2020-11	6.8	20.2
2020-12	7.0	17.8
2021-01	7.1	17.1
2021-02	7.2	16.9
2021-03	7.1	14.7
2021-04	7.4	16.9
2021-05	6.9	19.6
2021-06	7.3	12.2
2021-07	8.0	15.2
2021-08	7.4	16.6
2021-09	7.3	12.1
2021-10	7.4	12.8
2021-11	7.8	11.4
2021-12	8.1	14.3
2022-01	9.0	37.6

© 2023 Ambrose N et al. *JAMA Network Open*.

eTable 11. WHO Variant Classifications From Subset of Patients With Genome Sequences (n = 13 703) by Treatment Group

Characteristic	Non-treated, No (%)	Treated, No (%)
No WHO equivalent	620 (0.4)	221 (0.9)
Epsilon	217 (0.2)	85 (0.3)
Alpha	879 (0.6)	206 (0.8)
Zeta	6 (<0.1)	2 (<0.1)
Eta	0 (0.0)	2 (<0.1)
Iota	16 (<0.1)	2 (<0.1)
Gamma	72 (0.1)	8 (<0.1)
Kappa	1 (<0.1)	0 (0.0)
Beta	8 (<0.1)	1 (<0.1)
Lambda	4 (<0.1)	0 (0.0)
Mu	61 (<0.1)	7 (<0.1)
Delta 21AI	574 (0.4)	351 (1.4)
Delta 21J	4,877 (3.4)	2,901 (11.5)
Omicron (99.9% BA.1)	2,105 (1.5)	477 (1.9)
Sequence unavailable	132,502 (93.3)	20,978 (83.1)

© 2023 Ambrose N et al. *JAMA Network Open*.

eFigure 5. Distribution of WHO Variant Call From Genomic Sequences by Index Date Year and Month

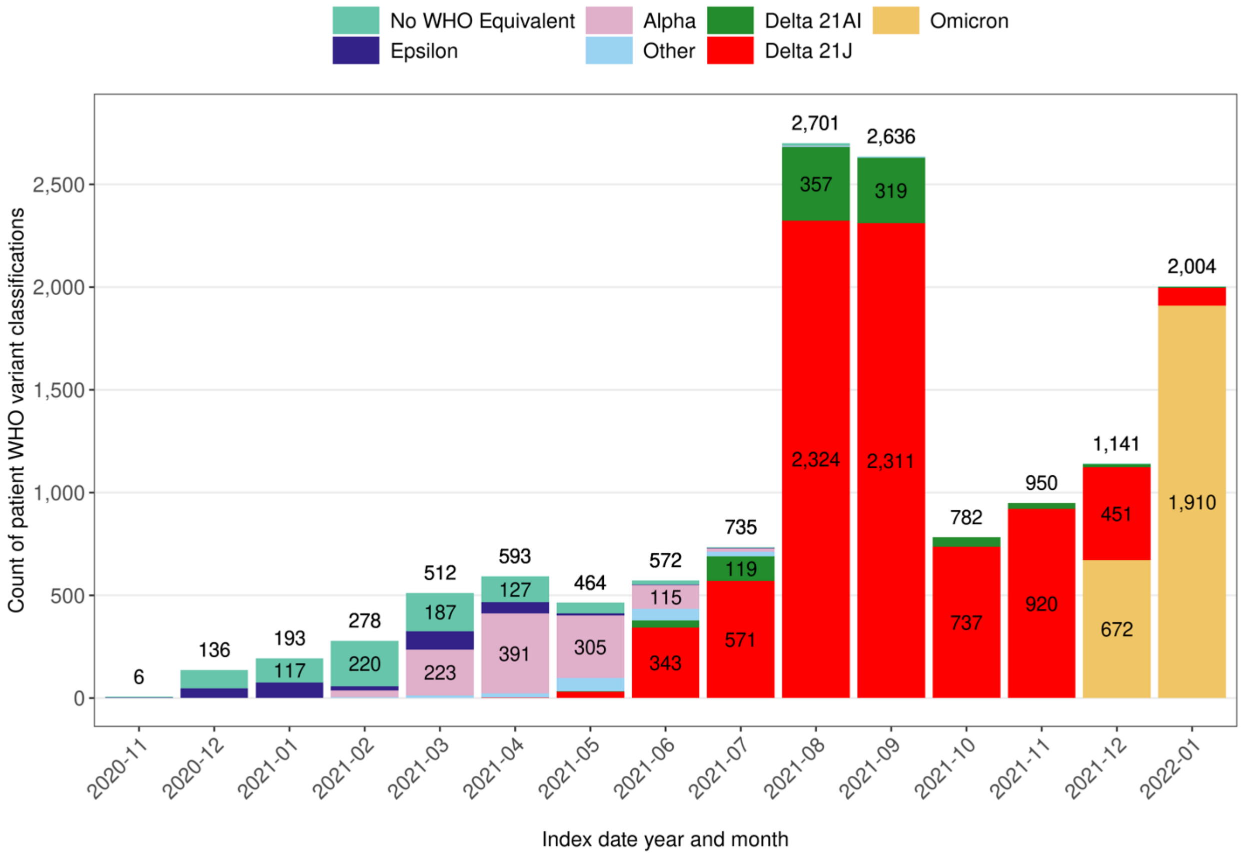

© 2023 Ambrose N et al. *JAMA Network Open*.

eTable 12. Treatment Effectiveness of nMAbs by COVID-19 Vaccination Status on 14- and 30-Day Outcomes

Number needed to treat (NNT) was not calculated (NC) when the probability difference between the treated versus non-treated was not significant. Significance in odds ratios may not match significance from probabilities due to the non-linear transformations from the log odds to the probabilities.

Outcome	Endpoint	Vaccination Status	Odds Ratio (95% CI)	Non-treated Probability, % (95% CI)	Treated Probability, % (95% CI)	NNT
Emergency department visit	14 days	No record of COVID-19 vaccination	0.76 (0.66 – 0.87)	5.9 (5.4 – 6.4)	4.5 (4.1 – 5.0)	73.9
		Partially vaccinated	0.81 (0.45 – 1.45)	4.5 (3.3 – 6.2)	3.7 (2.3 – 5.8)	NC
		Fully vaccinated	0.78 (0.64 – 0.97)	2.8 (2.5 – 3.3)	2.2 (1.9 – 2.6)	NC
		Fully vaccinated + booster	0.47 (0.21 – 1.05)	1.4 (1.1 – 1.8)	0.7 (0.3 – 1.4)	NC
	30 days	No record of COVID-19 vaccination	0.83 (0.74 – 0.94)	7.2 (6.7 – 7.8)	6.1 (5.6 – 6.6)	88.4
		Partially vaccinated	0.82 (0.51 – 1.31)	6.5 (5.0 – 8.3)	5.4 (3.7 – 7.7)	NC
		Fully vaccinated	0.88 (0.74 – 1.04)	4.1 (3.6 – 4.6)	3.6 (3.2 – 4.0)	NC
		Fully vaccinated + booster	0.68 (0.40 – 1.16)	2.6 (2.1 – 3.3)	1.8 (1.1 – 2.9)	NC
Hospitalization	14 days	No record of COVID-19 vaccination	0.51 (0.44 – 0.59)	6.0 (5.7 – 6.4)	3.2 (2.8 – 3.6)	34.7
		Partially vaccinated	0.37 (0.19 – 0.72)	3.6 (2.5 – 5.2)	1.3 (0.8 – 2.3)	44.4
		Fully vaccinated	0.51 (0.39 – 0.67)	3.4 (2.9 – 4.1)	1.8 (1.5 – 2.2)	60
		Fully vaccinated + booster	0.91 (0.49 – 1.72)	1.8 (1.3 – 2.4)	1.6 (1.0 – 2.8)	NC
	30 days	No record of COVID-19 vaccination	0.61 (0.53 – 0.71)	6.9 (6.5 – 7.3)	4.3 (3.8 – 4.9)	39.4
		Partially vaccinated	0.39 (0.22 – 0.69)	4.8 (3.6 – 6.5)	1.9 (1.2 – 3.1)	34.8
		Fully vaccinated	0.66 (0.52 – 0.83)	4.5 (3.9 – 5.2)	3.0 (2.5 – 3.6)	66.5
		Fully vaccinated + booster	1.26 (0.74 – 2.16)	3.1 (2.4 – 3.8)	3.8 (2.4 – 6.1)	NC
Death	14 days	No record of COVID-19 vaccination	0.18 (0.06 – 0.53)	0.5 (0.4 – 0.5)	0.1 (0.0 – 0.2)	264.7
		Partially vaccinated	0.78 (0.05 – 12.89)	0.3 (0.2 – 0.5)	0.2 (0.0 – 3.6)	NC
		Fully vaccinated	0.11 (0.02 – 0.58)	0.5 (0.5 – 0.6)	0.1 (0.0 – 0.3)	225
		Fully vaccinated + booster	0.52 (0.04 – 6.79)	0.4 (0.3 – 0.5)	0.2 (0.0 – 2.7)	NC
	30 days	No record of COVID-19 vaccination	0.15 (0.07 – 0.34)	1.0 (0.9 – 1.0)	0.1 (0.1 – 0.3)	122.9
		Partially vaccinated	0.33 (0.02 – 5.30)	0.7 (0.5 – 0.9)	0.2 (0.0 – 3.6)	NC
		Fully vaccinated	0.18 (0.07 – 0.49)	0.8 (0.8 – 0.9)	0.2 (0.1 – 0.4)	146

© 2023 Ambrose N et al. *JAMA Network Open*.

Outcome	Endpoint	Vaccination Status	Odds Ratio (95% CI)	Non-treated Probability, % (95% CI)	Treated Probability, % (95% CI)	NNT
		Fully vaccinated + booster	0.45 (0.04 – 4.55)	0.6 (0.5 – 0.7)	0.3 (0.0 – 2.6)	NC
Hospitalization or death	14 days	No record of COVID-19 vaccination	0.49 (0.42 – 0.56)	6.4 (6.0 – 6.8)	3.2 (2.8 – 3.6)	31.5
		Partially vaccinated	0.35 (0.18 – 0.68)	3.8 (2.6 – 5.4)	1.3 (0.8 – 2.3)	41.4
		Fully vaccinated	0.47 (0.36 – 0.61)	3.8 (3.2 – 4.5)	1.8 (1.5 – 2.2)	50.7
		Fully vaccinated + booster	0.76 (0.40 – 1.43)	2.2 (1.6 – 3.0)	1.7 (1.0 – 2.8)	NC
	30 days	No record of COVID-19 vaccination	0.58 (0.51 – 0.67)	7.3 (6.9 – 7.7)	4.4 (3.9 – 5.0)	34.5
		Partially vaccinated	0.36 (0.21 – 0.64)	5.2 (3.9 – 6.9)	1.9 (1.2 – 3.1)	30.8
		Fully vaccinated	0.60 (0.48 – 0.76)	5.0 (4.3 – 5.8)	3.1 (2.6 – 3.6)	52.5
		Fully vaccinated + booster	1.11 (0.65 – 1.89)	3.5 (2.8 – 4.4)	3.9 (2.4 – 6.1)	NC

© 2023 Ambrose N et al. *JAMA Network Open*.

eTable 13. Treatment Effectiveness of nMAbs by Immunocompromised Status on 14- and 30-Day Outcomes

Number needed to treat (NNT) was not calculated (NC) when the probability difference between the treated versus non-treated was not significant. Significance in odds ratios may not match significance from probabilities due to the non-linear transformations from the log odds to the probabilities.

Outcome	Endpoint	Immunocompromised Status	Odds Ratio (95% CI)	Non-treated Probability, % (95% CI)	Treated Probability, % (95% CI)	NNT
Emergency department visit	14 days	Not immunocompromised	0.83 (0.75 – 0.93)	4.3 (4.1 – 4.5)	3.6 (3.3 – 3.9)	146.1
		Immunocompromised	0.47 (0.32 – 0.69)	7.0 (5.1 – 9.6)	3.4 (2.8 – 4.1)	27.6
	30 days	Not immunocompromised	0.90 (0.82 – 0.99)	5.4 (5.2 – 5.6)	4.9 (4.6 – 5.3)	NC
		Immunocompromised	0.58 (0.43 – 0.79)	9.3 (7.3 – 11.8)	5.7 (4.9 – 6.6)	27.3
Hospitalization	14 days	Not immunocompromised	0.58 (0.50 – 0.67)	4.3 (4.0 – 4.6)	2.5 (2.2 – 2.9)	56.9
		Immunocompromised	0.31 (0.24 – 0.41)	8.9 (7.6 – 10.5)	3.0 (2.5 – 3.6)	16.8
	30 days	Not immunocompromised	0.70 (0.61 – 0.80)	5.1 (4.8 – 5.4)	3.6 (3.2 – 4.0)	67
		Immunocompromised	0.47 (0.37 – 0.59)	10.9 (9.4 – 12.5)	5.4 (4.6 – 6.4)	18.4
Death	14 days	Not immunocompromised	0.13 (0.07 – 0.24)	0.4 (0.3 – 0.5)	0.1 (0.0 – 0.1)	275
		Immunocompromised	0.07 (0.02 – 0.26)	0.8 (0.4 – 1.4)	0.1 (0.0 – 0.2)	142.3
	30 days	Not immunocompromised	0.14 (0.09 – 0.22)	0.8 (0.6 – 1.0)	0.1 (0.1 – 0.2)	148.3
		Immunocompromised	0.13 (0.06 – 0.27)	1.6 (1.0 – 2.4)	0.2 (0.1 – 0.4)	73.4
Hospitalization or death	14 days	Not immunocompromised	0.55 (0.47 – 0.63)	4.6 (4.3 – 4.9)	2.6 (2.3 – 2.9)	49.1
		Immunocompromised	0.30 (0.23 – 0.39)	9.4 (8.0 – 11.1)	3.0 (2.5 – 3.7)	15.6
	30 days	Not immunocompromised	0.65 (0.57 – 0.75)	5.5 (5.2 – 5.8)	3.7 (3.3 – 4.1)	54.9
		Immunocompromised	0.44 (0.35 – 0.56)	11.7 (10.2 – 13.4)	5.5 (4.7 – 6.5)	16.3

© 2023 Ambrose N et al. *JAMA Network Open*.

eTable 14. Treatment Effectiveness of nMAbs by Risk of Severe COVID-19 From the Disease Risk Score Model on 14- and 30-Day Outcomes

Number needed to treat (NNT) was not calculated (NC) when the probability difference between the treated versus non-treated was not significant. Significance in odds ratios may not match significance from probabilities due to the non-linear transformations from the log odds to the probabilities.

Outcome	Endpoint	Risk of Severe COVID-19	Odds Ratio (95% CI)	Non-treated Probability, % (95% CI)	Treated Probability, % (95% CI)	NNT
Emergency department visit	14 days	Strata 1 (lowest risk)	0.65 (0.41 – 1.05)	1.7 (1.6 – 1.9)	1.1 (0.7 – 1.8)	NC
		Strata 2	0.91 (0.73 – 1.14)	3.2 (3.0 – 3.4)	2.9 (2.4 – 3.6)	NC
		Strata 3	0.74 (0.64 – 0.86)	5.0 (4.7 – 5.3)	3.7 (3.3 – 4.3)	81.4
		Strata 4	0.73 (0.59 – 0.90)	6.2 (5.7 – 6.8)	4.6 (3.8 – 5.5)	62.1
		Strata 5 (highest risk)	0.60 (0.43 – 0.85)	7.8 (5.9 – 10.2)	4.9 (4.2 – 5.6)	34.2
	30 days	Strata 1 (lowest risk)	0.61 (0.41 – 0.90)	2.6 (2.4 – 2.8)	1.6 (1.1 – 2.3)	99.6
		Strata 2	0.96 (0.80 – 1.15)	4.3 (4.1 – 4.5)	4.1 (3.5 – 4.9)	NC
		Strata 3	0.88 (0.77 – 1.01)	6.3 (5.9 – 6.6)	5.6 (5.0 – 6.3)	NC
		Strata 4	0.80 (0.67 – 0.96)	7.5 (6.8 – 8.2)	6.1 (5.3 – 7.0)	NC
		Strata 5 (highest risk)	0.66 (0.50 – 0.88)	10.1 (8.1 – 12.5)	6.9 (6.1 – 7.9)	31.4
Hospitalization	14 days	Strata 1 (lowest risk)	1.83 (0.80 – 4.21)	0.3 (0.3 – 0.4)	0.6 (0.3 – 1.3)	NC
		Strata 2	0.89 (0.63 – 1.27)	1.2 (1.1 – 1.3)	1.1 (0.8 – 1.5)	NC
		Strata 3	0.58 (0.48 – 0.72)	3.7 (3.4 – 4.1)	2.2 (1.8 – 2.6)	66
		Strata 4	0.45 (0.36 – 0.57)	7.4 (6.8 – 8.1)	3.5 (2.8 – 4.3)	25.4
		Strata 5 (highest risk)	0.41 (0.32 – 0.53)	15.5 (13.7 – 17.4)	7.0 (5.8 – 8.5)	11.8
	30 days	Strata 1 (lowest risk)	3.15 (1.64 – 6.05)	0.5 (0.4 – 0.6)	1.6 (0.8 – 2.9)	-93.6
		Strata 2	1.11 (0.81 – 1.51)	1.5 (1.4 – 1.7)	1.7 (1.3 – 2.3)	NC
		Strata 3	0.63 (0.52 – 0.76)	4.5 (4.2 – 4.9)	2.9 (2.5 – 3.4)	62.1
		Strata 4	0.57 (0.45 – 0.72)	8.9 (8.2 – 9.6)	5.3 (4.3 – 6.4)	27.6
		Strata 5 (highest risk)	0.52 (0.42 – 0.64)	17.9 (16.0 – 19.9)	10.2 (8.8 – 11.8)	13
Death	14 days	Strata 1 (lowest risk)	Non convergence	0.0 (0.0 – 0.0)	0.2 (0.0 – 3.6)	NC
		Strata 2	0.70 (0.04 – 11.35)	0.1 (0.0 – 0.1)	0.0 (0.0 – 0.7)	NC

© 2023 Ambrose N et al. *JAMA Network Open*.

Outcome	Endpoint	Risk of Severe COVID-19	Odds Ratio (95% CI)	Non-treated Probability, % (95% CI)	Treated Probability, % (95% CI)	NNT
		Strata 3	0.23 (0.03 – 1.83)	0.1 (0.1 – 0.2)	0.0 (0.0 – 0.3)	NC
		Strata 4	0.11 (0.02 – 0.50)	0.5 (0.5 – 0.6)	0.1 (0.0 – 0.3)	219.3
		Strata 5 (highest risk)	0.17 (0.08 – 0.38)	2.2 (2.1 – 2.4)	0.4 (0.2 – 0.9)	54.2
	30 days	Strata 1 (lowest risk)	Non convergence	0.0 (0.0 – 0.0)	0.2 (0.0 – 3.6)	NC
		Strata 2	0.54 (0.03 – 8.82)	0.1 (0.1 – 0.1)	0.0 (0.0 – 0.7)	NC
		Strata 3	0.22 (0.05 – 0.96)	0.3 (0.3 – 0.4)	0.1 (0.0 – 0.3)	NC
		Strata 4	0.16 (0.07 – 0.38)	1.1 (1.0 – 1.2)	0.2 (0.1 – 0.4)	110.9
		Strata 5 (highest risk)	0.15 (0.08 – 0.28)	4.2 (4.0 – 4.4)	0.7 (0.4 – 1.2)	28.5
Hospitalization or death	14 days	Strata 1 (lowest risk)	1.81 (0.79 – 4.15)	0.3 (0.3 – 0.4)	0.6 (0.3 – 1.3)	NC
		Strata 2	0.86 (0.60 – 1.22)	1.3 (1.1 – 1.4)	1.1 (0.8 – 1.5)	NC
		Strata 3	0.57 (0.47 – 0.70)	3.8 (3.5 – 4.2)	2.2 (1.9 – 2.7)	61.9
		Strata 4	0.44 (0.34 – 0.55)	7.8 (7.1 – 8.4)	3.5 (2.9 – 4.3)	23.6
		Strata 5 (highest risk)	0.38 (0.30 – 0.48)	17.1 (15.3 – 19.1)	7.2 (6.0 – 8.7)	10.1
	30 days	Strata 1 (lowest risk)	3.11 (1.62 – 5.97)	0.5 (0.4 – 0.6)	1.6 (0.8 – 2.9)	-94.2
		Strata 2	1.07 (0.78 – 1.46)	1.6 (1.5 – 1.8)	1.7 (1.3 – 2.3)	NC
		Strata 3	0.62 (0.51 – 0.75)	4.7 (4.3 – 5.1)	2.9 (2.5 – 3.5)	58.2
		Strata 4	0.55 (0.43 – 0.69)	9.3 (8.6 – 10.1)	5.3 (4.3 – 6.5)	24.9
		Strata 5 (highest risk)	0.47 (0.38 – 0.57)	20.1 (18.1 – 22.2)	10.5 (9.1 – 12.1)	10.5

© 2023 Ambrose N et al. *JAMA Network Open*.

eTable 15. nMAb Effectiveness by Variant Epoch

Number needed to treat (NNT) was not calculated (NC) when the probability difference between the treated versus non-treated was not significant. Significance in odds ratios may not match significance from probabilities due to the non-linear transformations from the log odds to the probabilities.

Outcome	Endpoint	Variant Epoch	Odds Ratio (95% CI)	Non-treated Probability, % (95% CI)	Treated Probability, % (95% CI)	NNT
Emergency department visit	14 days	pre-Delta (2020-11 to 2021-06)	0.80 (0.69 – 0.93)	4.9 (4.6 – 5.2)	4.0 (3.5 – 4.5)	108.1
		Delta (2021-07 to 2021-11)	0.75 (0.65 – 0.85)	5.3 (4.9 – 5.8)	4.0 (3.7 – 4.4)	76.7
		Delta/Omicron BA. 1 (2021-12)	0.77 (0.43 – 1.37)	4.4 (2.6 – 7.1)	3.4 (2.7 – 4.3)	NC
		Omicron BA. 1 (2022-01)	0.67 (0.32 – 1.38)	2.5 (2.4 – 2.7)	1.7 (0.8 – 3.4)	NC
	30 days	pre-Delta (2020-11 to 2021-06)	0.87 (0.77 – 0.99)	6.2 (5.9 – 6.5)	5.5 (4.9 – 6.1)	NC
		Delta (2021-07 to 2021-11)	0.81 (0.72 – 0.91)	6.7 (6.2 – 7.3)	5.5 (5.1 – 5.9)	81.8
		Delta/Omicron BA. 1 (2021-12)	0.94 (0.59 – 1.48)	5.5 (3.7 – 8.1)	5.2 (4.3 – 6.3)	NC
		Omicron BA. 1 (2022-01)	0.75 (0.45 – 1.26)	3.7 (3.5 – 3.9)	2.8 (1.7 – 4.6)	NC
Hospitalization	14 days	pre-Delta (2020-11 to 2021-06)	0.59 (0.50 – 0.71)	5.2 (4.8 – 5.8)	3.2 (2.8 – 3.7)	48.5
		Delta (2021-07 to 2021-11)	0.37 (0.31 – 0.43)	6.2 (5.7 – 6.9)	2.4 (2.1 – 2.6)	25.9
		Delta/Omicron BA. 1 (2021-12)	0.70 (0.51 – 0.98)	3.5 (2.9 – 4.3)	2.5 (1.9 – 3.2)	NC
		Omicron BA. 1 (2022-01)	1.29 (0.68 – 2.47)	1.7 (1.5 – 1.9)	2.2 (1.2 – 4.1)	NC
	30 days	pre-Delta (2020-11 to 2021-06)	0.67 (0.58 – 0.78)	6.2 (5.7 – 6.7)	4.2 (3.8 – 4.8)	51.1
		Delta (2021-07 to 2021-11)	0.42 (0.37 – 0.49)	7.2 (6.6 – 7.9)	3.2 (2.9 – 3.5)	24.9
		Delta/Omicron BA. 1 (2021-12)	0.81 (0.61 – 1.07)	4.5 (3.8 – 5.3)	3.7 (3.0 – 4.5)	NC
		Omicron BA. 1 (2022-01)	2.10 (1.37 – 3.21)	2.5 (2.3 – 2.7)	5.1 (3.4 – 7.5)	-38.7
Death	14 days	pre-Delta (2020-11 to 2021-06)	0.26 (0.05 – 1.24)	0.4 (0.3 – 0.4)	0.1 (0.0 – 0.5)	NC
		Delta (2021-07 to 2021-11)	0.13 (0.05 – 0.30)	0.6 (0.6 – 0.7)	0.1 (0.0 – 0.2)	177.9
		Delta/Omicron BA. 1 (2021-12)	0.19 (0.02 – 1.72)	0.5 (0.4 – 0.6)	0.1 (0.0 – 0.8)	NC
		Omicron BA. 1 (2022-01)	2.30 (0.14 – 37.33)	0.1 (0.1 – 0.2)	0.3 (0.0 – 4.4)	NC
	30 days	pre-Delta (2020-11 to 2021-06)	0.16 (0.08 – 0.33)	0.9 (0.6 – 1.3)	0.1 (0.1 – 0.3)	136.9
		Delta (2021-07 to 2021-11)	0.14 (0.09 – 0.22)	1.2 (0.9 – 1.5)	0.2 (0.1 – 0.2)	97
		Delta/Omicron BA. 1 (2021-12)	0.10 (0.03 – 0.35)	0.8 (0.5 – 1.2)	0.1 (0.0 – 0.2)	143

© 2023 Ambrose N et al. *JAMA Network Open*.

Outcome	Endpoint	Variant Epoch	Odds Ratio (95% CI)	Non-treated Probability, % (95% CI)	Treated Probability, % (95% CI)	NNT
		Omicron BA. 1 (2022-01)	0.13 (0.02 – 0.93)	0.2 (0.1 – 0.2)	0.0 (0.0 – 0.2)	NC
Hospitalization or death	14 days	pre-Delta (2020-11 to 2021-06)	0.58 (0.49 – 0.68)	5.5 (5.0 – 6.0)	3.2 (2.8 – 3.7)	44.4
		Delta (2021-07 to 2021-11)	0.34 (0.30 – 0.40)	6.7 (6.1 – 7.4)	2.4 (2.2 – 2.7)	23.2
		Delta/Omicron BA. 1 (2021-12)	0.65 (0.47 – 0.90)	3.9 (3.2 – 4.7)	2.5 (2.0 – 3.3)	NC
		Omicron BA. 1 (2022-01)	1.22 (0.64 – 2.33)	1.8 (1.6 – 2.0)	2.2 (1.2 – 4.1)	NC
	30 days	pre-Delta (2020-11 to 2021-06)	0.65 (0.56 – 0.75)	6.6 (6.1 – 7.1)	4.4 (3.9 – 4.9)	45.2
		Delta (2021-07 to 2021-11)	0.40 (0.35 – 0.45)	7.8 (7.2 – 8.5)	3.3 (3.0 – 3.6)	21.9
		Delta/Omicron BA. 1 (2021-12)	0.74 (0.56 – 0.97)	5.0 (4.2 – 5.8)	3.7 (3.0 – 4.6)	NC
		Omicron BA. 1 (2022-01)	2.00 (1.31 – 3.07)	2.6 (2.4 – 2.8)	5.1 (3.4 – 7.5)	-40.5

© 2023 Ambrose N et al. *JAMA Network Open*.

eTable 16. nMAb Treatment Effectiveness by Product and Variant Epoch

Number needed to treat (NNT) was not calculated (NC) when the probability difference between the treated versus non-treated was not significant. Odds ratios and NNTs for nMAb products not used as therapeutics during a variant epoch are represented with '-'.

			Pre-Delta (2020-11 to 2021-06)		Delta (2021-07 to 2021-11)		Delta/Omicron BA.1 (2021-12)		Omicron BA.1 (2022-01)	
**Outcome**	**Endpoint**	**nMAb Product**	**Odds Ratio (95% CI)**	**NNT**	**Odds Ratio (95% CI)**	**NNT**	**Odds Ratio (95% CI)**	**NNT**	**Odds Ratio (95% CI)**	**NNT**
Emergency department visit	14 days	Bamlanivimab	0.86 (0.73 – 1.03)	NC	-	-	-	-	-	-
		Casirivimab-imdevimab	0.81 (0.51 – 1.29)	NC	0.75 (0.66 – 0.86)	79.3	0.80 (0.43 – 1.46)	NC	3.32 (0.50 – 21.93)	NC
		Bamlanivimab-etesevimab	0.77 (0.49 – 1.20)	NC	0.46 (0.26 – 0.82)	35.9	0.82 (0.40 – 1.70)	NC	0.19 (0.02 – 1.50)	NC
		Sotrovimab	-	-	0.23 (0.10 – 0.51)	24.6	0.51 (0.22 – 1.19)	NC	0.58 (0.26 – 1.29)	NC
	30 days	Bamlanivimab	0.91 (0.79 – 1.06)	NC	-	-	-	-	-	-
		Casirivimab-imdevimab	0.88 (0.61 – 1.27)	NC	0.81 (0.72 – 0.92)	85.5	0.92 (0.57 – 1.50)	NC	3.03 (0.65 – 14.07)	NC
		Bamlanivimab-etesevimab	0.87 (0.59 – 1.30)	NC	0.54 (0.32 – 0.91)	33.7	1.05 (0.60 – 1.86)	NC	0.13 (0.02 – 1.00)	NC
		Sotrovimab	-	-	0.38 (0.21 – 0.68)	24.7	0.81 (0.42 – 1.55)	NC	0.69 (0.40 – 1.19)	NC
Hospitalization	14 days	Bamlanivimab	0.56 (0.45 – 0.68)	44.3	-	-	-	-	-	-
		Casirivimab-imdevimab	0.60 (0.43 – 0.84)	49.1	0.37 (0.32 – 0.43)	26	0.71 (0.47 – 1.05)	NC	0.11 (0.01 – 1.71)	NC
		Bamlanivimab-etesevimab	0.75 (0.49 – 1.15)	NC	0.31 (0.16 – 0.61)	23.8	0.63 (0.36 – 1.10)	NC	0.65 (0.20 – 2.10)	NC
		Sotrovimab	-	-	0.22 (0.11 – 0.44)	20.8	0.83 (0.47 – 1.45)	NC	1.38 (1.16 – 1.64)	-158.3
	30 days	Bamlanivimab	0.67 (0.56 – 0.80)	50.8	-	-	-	-	-	-
		Casirivimab-imdevimab	0.61 (0.45 – 0.82)	43	0.42 (0.37 – 0.49)	24.9	0.76 (0.54 – 1.08)	NC	0.07 (0.00 – 1.17)	NC
		Bamlanivimab-etesevimab	0.71 (0.48 – 1.05)	NC	0.35 (0.19 – 0.64)	21.9	0.77 (0.49 – 1.23)	NC	0.44 (0.14 – 1.43)	NC
		Sotrovimab	-	-	0.39 (0.23 – 0.64)	23.3	1.11 (0.70 – 1.76)	NC	2.27 (2.00 – 2.56)	-33.8
Death	14 days	Bamlanivimab	0.28 (0.14 – 0.57)	358.3	-	-	-	-	-	-
		Casirivimab-imdevimab	0.07 (0.01 – 0.53)	278.1	0.13 (0.07 – 0.21)	177.7	0.13 (0.04 – 0.47)	242.7	1.49 (0.09 – 24.19)	NC

© 2023 Ambrose N et al. *JAMA Network Open*.

			Pre-Delta (2020-11 to 2021-06)		Delta (2021-07 to 2021-11)		Delta/Omicron BA.1 (2021-12)		Omicron BA.1 (2022-01)	
**Outcome**	**Endpoint**	**nMAb Product**	**Odds Ratio (95% CI)**	**NNT**	**Odds Ratio (95% CI)**	**NNT**	**Odds Ratio (95% CI)**	**NNT**	**Odds Ratio (95% CI)**	**NNT**
		Bamlanivimab-etesevimab	0.40 (0.03 – 6.48)	NC	0.12 (0.01 – 1.88)	NC	0.08 (0.00 – 1.29)	NC	1.59 (0.10 – 25.80)	NC
		Sotrovimab	-	-	0.31 (0.02 – 4.91)	NC	0.15 (0.01 – 2.43)	NC	0.06 (0.00 – 1.01)	NC
	30 days	Bamlanivimab	0.24 (0.15 – 0.40)	151.8	-	-	-	-	-	-
		Casirivimab-imdevimab	0.07 (0.02 – 0.27)	123.2	0.15 (0.10 – 0.21)	97.9	0.13 (0.05 – 0.35)	147.9	1.01 (0.06 – 16.25)	NC
		Bamlanivimab-etesevimab	0.18 (0.01 – 2.87)	NC	0.06 (0.00 – 1.00)	NC	0.05 (0.00 – 0.78)	134.9	1.07 (0.07 – 17.32)	NC
		Sotrovimab	-	-	0.28 (0.03 – 2.35)	NC	0.30 (0.06 – 1.40)	NC	0.18 (0.05 – 0.70)	665.7
Hospitalization or death	14 days	Bamlanivimab	0.55 (0.45 – 0.67)	41.4	-	-	-	-	-	-
		Casirivimab-imdevimab	0.57 (0.41 – 0.80)	44	0.35 (0.30 – 0.40)	23.3	0.65 (0.44 – 0.97)	NC	0.10 (0.01 – 1.61)	NC
		Bamlanivimab-etesevimab	0.71 (0.47 – 1.09)	NC	0.29 (0.15 – 0.56)	21.3	0.58 (0.33 – 1.00)	NC	0.61 (0.19 – 1.98)	NC
		Sotrovimab	-	-	0.20 (0.10 – 0.40)	18.9	0.75 (0.43 – 1.32)	NC	1.31 (1.10 – 1.55)	186.4
	30 days	Bamlanivimab	0.66 (0.55 – 0.78)	46.4	-	-	-	-	-	-
		Casirivimab-imdevimab	0.58 (0.43 – 0.78)	37.4	0.40 (0.35 – 0.46)	21.9	0.70 (0.50 – 0.98)	NC	0.07 (0.00 – 1.11)	NC
		Bamlanivimab-etesevimab	0.67 (0.45 – 0.99)	NC	0.32 (0.17 – 0.58)	19.2	0.70 (0.44 – 1.11)	NC	0.42 (0.13 – 1.37)	NC
		Sotrovimab	-	-	0.37 (0.23 – 0.60)	20.9	1.01 (0.64 – 1.59)	NC	2.16 (1.91 – 2.44)	-35.1

© 2023 Ambrose N et al. *JAMA Network Open*.

eTable 17. nMAb Effectiveness by Variant Sequence

Number needed to treat (NNT) was not calculated (NC) when the probability difference between the treated versus non-treated was not significant. Significance in odds ratios may not match significance from probabilities due to the non-linear transformations from the log odds to the probabilities.

Outcome	Endpoint	Variant Sequence	Odds Ratio (95% CI)	Non-treated Probability, % (95% CI)	Treated Probability, % (95% CI)	NNT
Emergency department visit	14 days	No WHO equivalent	0.87 (0.34 – 2.25)	4.7 (3.5 – 6.3)	4.1 (1.7 – 9.5)	NC
		Epsilon	0.95 (0.21 – 4.29)	5.2 (3.2 – 8.4)	4.9 (1.2 – 17.7)	NC
		Alpha	0.66 (0.25 – 1.76)	7.2 (5.9 – 8.7)	4.8 (1.9 – 11.7)	NC
		Other (e.g., Mu, Iota)	0.85 (0.05 – 15.64)	8.7 (5.8 – 12.8)	7.5 (0.5 – 59.2)	NC
		Delta 21AI	0.64 (0.33 – 1.24)	7.9 (6.5 – 9.6)	5.2 (2.9 – 9.3)	NC
		Delta 21J	0.67 (0.51 – 0.88)	5.5 (5.1 – 6.0)	3.7 (2.9 – 4.8)	57.4
		Omicron BA.1	0.85 (0.42 – 1.75)	2.6 (2.0 – 3.3)	2.2 (1.2 – 4.3)	NC
	30 days	No WHO equivalent	1.05 (0.48 – 2.31)	6.0 (4.6 – 7.7)	6.3 (3.1 – 12.3)	NC
		Epsilon	1.67 (0.55 – 5.04)	6.5 (4.2 – 10.0)	10.5 (4.1 – 24.2)	NC
		Alpha	0.99 (0.45 – 2.17)	8.1 (6.7 – 9.7)	8.0 (3.9 – 15.6)	NC
		Other (e.g., Mu, Iota)	0.76 (0.04 – 14.03)	9.6 (6.6 – 13.9)	7.5 (0.5 – 59.2)	NC
		Delta 21AI	0.65 (0.36 – 1.20)	9.2 (7.7 – 11.0)	6.2 (3.6 – 10.5)	NC
		Delta 21J	0.74 (0.58 – 0.93)	6.6 (6.1 – 7.1)	4.9 (4.0 – 6.1)	NC
		Omicron BA.1	0.79 (0.44 – 1.43)	4.1 (3.4 – 5.0)	3.3 (1.9 – 5.6)	NC
Hospitalization	14 days	No WHO equivalent	0.52 (0.24 – 1.12)	6.8 (4.8 – 9.4)	3.6 (1.8 – 6.9)	NC
		Epsilon	0.18 (0.02 – 1.52)	5.2 (2.5 – 10.4)	1.0 (0.1 – 6.8)	NC
		Alpha	0.57 (0.26 – 1.24)	7.0 (5.3 – 9.2)	4.1 (2.1 – 8.1)	NC
		Other (e.g., Mu, Iota)	0.22 (0.03 – 1.79)	13.9 (8.0 – 23.2)	3.4 (0.5 – 20.8)	NC
		Delta 21AI	0.08 (0.02 – 0.27)	10.4 (6.6 – 16.0)	0.9 (0.3 – 2.7)	10.5
		Delta 21J	0.30 (0.21 – 0.42)	5.9 (4.9 – 7.2)	1.8 (1.4 – 2.4)	24.3
		Omicron BA.1	0.65 (0.25 – 1.72)	2.3 (1.7 – 3.2)	1.5 (0.6 – 3.7)	NC
	30 days	No WHO equivalent	0.48 (0.23 – 1.01)	7.6 (5.6 – 10.3)	3.8 (2.0 – 7.1)	NC
		Epsilon	0.24 (0.05 – 1.12)	7.5 (4.0 – 13.5)	1.9 (0.5 – 7.2)	NC

© 2023 Ambrose N et al. *JAMA Network Open*.

Outcome	Endpoint	Variant Sequence	Odds Ratio (95% CI)	Non-treated Probability, % (95% CI)	Treated Probability, % (95% CI)	NNT
		Alpha	0.54 (0.25 – 1.16)	7.4 (5.7 – 9.7)	4.1 (2.1 – 8.1)	NC
		Other (e.g., Mu, Iota)	0.22 (0.03 – 1.79)	13.9 (8.0 – 23.2)	3.4 (0.5 – 20.8)	NC
		Delta 21AI	0.08 (0.03 – 0.25)	11.7 (7.7 – 17.4)	1.1 (0.4 – 2.9)	9.4
		Delta 21J	0.35 (0.25 – 0.48)	6.8 (5.6 – 8.3)	2.5 (2.0 – 3.1)	23
		Omicron BA.1	1.70 (0.91 – 3.17)	2.9 (2.2 – 3.9)	4.8 (2.8 – 8.1)	NC
Death	14 days	No WHO equivalent	1.87 (0.08 – 41.41)	0.2 (0.1 – 0.9)	0.4 (0.0 – 6.3)	NC
		Epsilon	1.32 (0.06 – 27.28)	0.9 (0.3 – 3.0)	1.2 (0.1 – 16.8)	NC
		Alpha	13.53 (0.27 – 685.84)	0.0 (0.0 – 0.6)	0.5 (0.0 – 8.1)	NC
		Other (e.g., Mu, Iota)	41.74 (0.76 – 2280.78)	0.2 (0.0 – 3.0)	7.5 (0.5 – 59.2)	NC
		Delta 21AI	0.60 (0.03 – 11.16)	0.4 (0.2 – 1.0)	0.2 (0.0 – 3.8)	NC
		Delta 21J	0.14 (0.02 – 1.15)	0.4 (0.3 – 0.5)	0.1 (0.0 – 0.4)	NC
		Omicron BA.1	0.31 (0.02 – 5.40)	0.4 (0.2 – 0.8)	0.1 (0.0 – 2.0)	NC
	30 days	No WHO equivalent	0.49 (0.03 – 8.54)	0.9 (0.4 – 1.7)	0.4 (0.0 – 6.3)	NC
		Epsilon	0.64 (0.07 – 5.72)	3.4 (1.8 – 6.2)	2.2 (0.3 – 15.4)	NC
		Alpha	0.93 (0.05 – 16.55)	0.6 (0.3 – 1.2)	0.5 (0.0 – 8.1)	NC
		Other (e.g., Mu, Iota)	10.21 (0.42 – 249.40)	0.8 (0.2 – 3.1)	7.5 (0.5 – 59.2)	NC
		Delta 21AI	0.17 (0.01 – 2.90)	1.4 (0.9 – 2.3)	0.2 (0.0 – 3.8)	NC
		Delta 21J	0.07 (0.01 – 0.58)	0.8 (0.6 – 0.9)	0.1 (0.0 – 0.4)	142.4
		Omicron BA.1	0.29 (0.02 – 4.91)	0.4 (0.2 – 0.8)	0.1 (0.0 – 2.0)	NC
Hospitalization or death	14 days	No WHO equivalent	0.50 (0.23 – 1.09)	6.9 (5.0 – 9.6)	3.6 (1.8 – 6.9)	NC
		Epsilon	0.16 (0.02 – 1.29)	6.0 (3.0 – 11.4)	1.0 (0.1 – 6.8)	NC
		Alpha	0.57 (0.26 – 1.24)	7.0 (5.3 – 9.2)	4.1 (2.1 – 8.1)	NC
		Other (e.g., Mu, Iota)	0.22 (0.03 – 1.79)	13.9 (8.0 – 23.2)	3.4 (0.5 – 20.8)	NC
		Delta 21AI	0.07 (0.02 – 0.26)	10.6 (6.8 – 16.2)	0.9 (0.3 – 2.7)	10.2
		Delta 21J	0.29 (0.20 – 0.40)	6.2 (5.1 – 7.5)	1.9 (1.4 – 2.4)	23.1
		Omicron BA.1	0.64 (0.24 – 1.69)	2.3 (1.7 – 3.2)	1.5 (0.6 – 3.7)	NC
	30 days	No WHO equivalent	0.45 (0.22 – 0.95)	8.1 (6.0 – 10.8)	3.8 (2.0 – 7.1)	NC
		Epsilon	0.28 (0.08 – 1.04)	9.4 (5.5 – 15.6)	2.9 (0.9 – 8.6)	NC

© 2023 Ambrose N et al. *JAMA Network Open*.

Outcome	Endpoint	Variant Sequence	Odds Ratio (95% CI)	Non-treated Probability, % (95% CI)	Treated Probability, % (95% CI)	NNT
		Alpha	0.53 (0.24 – 1.14)	7.5 (5.8 – 9.8)	4.1 (2.1 – 8.1)	NC
		Other (e.g., Mu, Iota)	0.22 (0.03 – 1.79)	13.9 (8.0 – 23.2)	3.4 (0.5 – 20.8)	NC
		Delta 21AI	0.08 (0.03 – 0.24)	11.9 (7.9 – 17.6)	1.1 (0.4 – 2.9)	9.2
		Delta 21J	0.33 (0.24 – 0.45)	7.2 (5.9 – 8.7)	2.5 (2.0 – 3.1)	21.3
		Omicron BA.1	1.68 (0.90 – 3.13)	3.0 (2.2 – 3.9)	4.8 (2.8 – 8.1)	NC

© 2023 Ambrose N et al. *JAMA Network Open*.

eTable 18. nMAb Treatment Effectiveness by Product and Variant Sequence: No WHO Equivalent, Epsilon, Alpha, and Other (eg, Mu and Iota)

Number needed to treat (NNT) was not calculated (NC) when the probability difference between the treated versus non-treated was not significant.

			No WHO equivalent		Epsilon		Alpha		Other (e.g., Mu, Iota)	
Outcome	Endpoint	nMAb Product	Odds Ratio (95% CI)	NNT	Odds Ratio (95% CI)	NNT	Odds Ratio (95% CI)	NNT	Odds Ratio (95% CI)	NNT
Emergency department visit	14 days	Bamlanivimab	0.67 (0.24 – 1.86)	NC	0.97 (0.33 – 2.84)	NC	0.32 (0.04 – 2.39)	NC	4.59 (0.16 – 134.35)	NC
		Casirivimab-imdevimab	0.36 (0.05 – 2.78)	NC	2.52 (0.12 – 51.27)	NC	1.63 (0.50 – 5.38)	NC	0.15 (0.01 – 2.63)	NC
		Bamlanivimab-etesevimab	1.42 (0.18 – 11.46)	NC	1.03 (0.06 – 18.92)	NC	0.40 (0.12 – 1.41)	NC	0.75 (0.04 – 13.89)	NC
	30 days	Bamlanivimab	0.60 (0.23 – 1.51)	NC	1.45 (0.62 – 3.39)	NC	0.66 (0.19 – 2.30)	NC	4.13 (0.14 – 120.54)	NC
		Casirivimab-imdevimab	0.72 (0.20 – 2.58)	NC	1.97 (0.10 – 39.75)	NC	1.62 (0.53 – 4.89)	NC	0.14 (0.01 – 2.36)	NC
		Bamlanivimab-etesevimab	1.10 (0.14 – 8.79)	NC	2.32 (0.34 – 15.75)	NC	0.71 (0.24 – 2.07)	NC	0.67 (0.04 – 12.46)	NC
Hospitalization	14 days	Bamlanivimab	0.68 (0.36 – 1.28)	NC	0.31 (0.06 – 1.64)	NC	0.94 (0.21 – 4.14)	NC	2.68 (0.09 – 77.58)	NC
		Casirivimab-imdevimab	1.08 (0.42 – 2.78)	NC	2.44 (0.12 – 49.55)	NC	0.75 (0.11 – 4.90)	NC	0.09 (0.01 – 1.51)	NC
		Bamlanivimab-etesevimab	0.30 (0.02 – 4.99)	NC	1.00 (0.05 – 18.29)	NC	0.38 (0.14 – 1.05)	NC	1.70 (0.29 – 10.10)	NC
	30 days	Bamlanivimab	0.64 (0.35 – 1.18)	NC	0.21 (0.04 – 1.10)	NC	0.88 (0.20 – 3.89)	NC	2.68 (0.09 – 77.58)	NC
		Casirivimab-imdevimab	0.95 (0.37 – 2.44)	NC	1.67 (0.08 – 33.65)	NC	0.70 (0.11 – 4.61)	NC	0.09 (0.01 – 1.51)	NC
		Bamlanivimab-etesevimab	0.26 (0.02 – 4.38)	NC	1.18 (0.12 – 11.87)	NC	0.36 (0.13 – 0.98)	NC	1.70 (0.29 – 10.10)	NC
Death	14 days	Bamlanivimab	0.88 (0.04 – 19.48)	NC	0.58 (0.03 – 12.13)	NC	40.51 (0.79 – 2084.74)	NC	226.05 (2.89 – 17683.20)	NC
		Casirivimab-imdevimab	3.27 (0.15 – 73.06)	NC	14.60 (0.60 – 358.27)	NC	11.66 (0.23 – 593.27)	NC	7.52 (0.14 – 395.37)	NC

© 2023 Ambrose N et al. *JAMA Network Open*.

			No WHO equivalent		Epsilon		Alpha		Other (e.g., Mu, Iota)	
Outcome	Endpoint	nMAb Product	Odds Ratio (95% CI)	NNT	Odds Ratio (95% CI)	NNT	Odds Ratio (95% CI)	NNT	Odds Ratio (95% CI)	NNT
		Bamlanivimab-etesevimab	9.72 (0.43 – 221.11)	NC	5.99 (0.27 – 133.07)	NC	4.75 (0.09 – 240.83)	NC	36.83 (0.66 – 2047.43)	NC
	30 days	Bamlanivimab	0.23 (0.01 – 4.01)	NC	0.50 (0.09 – 2.75)	NC	2.80 (0.16 – 50.44)	NC	55.29 (1.47 – 2073.47)	NC
		Casirivimab-imdevimab	0.85 (0.05 – 15.06)	NC	3.94 (0.19 – 82.03)	NC	0.81 (0.05 – 14.29)	NC	1.84 (0.08 – 42.29)	NC
		Bamlanivimab-etesevimab	2.53 (0.14 – 45.65)	NC	1.62 (0.09 – 30.30)	NC	0.33 (0.02 – 5.80)	NC	9.01 (0.37 – 222.18)	NC
Hospitalization or death	14 days	Bamlanivimab	0.66 (0.35 – 1.25)	NC	0.27 (0.05 – 1.41)	NC	0.94 (0.21 – 4.14)	NC	2.68 (0.09 – 77.58)	NC
		Casirivimab-imdevimab	1.05 (0.41 – 2.71)	NC	2.11 (0.10 – 42.79)	NC	0.75 (0.11 – 4.90)	NC	0.09 (0.01 – 1.51)	NC
		Bamlanivimab-etesevimab	0.29 (0.02 – 4.86)	NC	0.87 (0.05 – 15.79)	NC	0.38 (0.14 – 1.05)	NC	1.70 (0.29 – 10.10)	NC
	30 days	Bamlanivimab	0.60 (0.33 – 1.11)	NC	0.28 (0.08 – 1.03)	NC	0.87 (0.20 – 3.84)	NC	2.68 (0.09 – 77.58)	NC
		Casirivimab-imdevimab	0.89 (0.35 – 2.29)	NC	1.31 (0.07 – 26.21)	NC	0.69 (0.11 – 4.55)	NC	0.09 (0.01 – 1.51)	NC
		Bamlanivimab-etesevimab	0.25 (0.01 – 4.11)	NC	0.92 (0.09 – 9.23)	NC	0.36 (0.13 – 0.97)	NC	1.70 (0.29 – 10.10)	NC

© 2023 Ambrose N et al. *JAMA Network Open*.

eTable 19. nMAb Treatment Effectiveness by Product and Variant Sequence: Delta 21AI, Delta 21J, and Omicron BA.1

Number needed to treat (NNT) was not calculated (NC) when the probability difference between the treated versus non-treated was not significant.

			Delta 21AI		Delta 21J		Omicron BA.1	
**Outcome**	**Endpoint**	**mAb Product**	**Odds Ratio (95% CI)**	**NNT**	**Odds Ratio (95% CI)**	**NNT**	**Odds Ratio (95% CI)**	**NNT**
Emergency department visit	14 days	Casirivimab-imdevimab	0.63 (0.40 – 0.99)	NC	0.65 (0.49 – 0.86)	53.7	0.06 (0.01 – 0.50)	41
		Bamlanivimab-etesevimab	0.50 (0.03 – 8.65)	NC	0.57 (0.25 – 1.30)	NC	1.24 (0.33 – 4.66)	NC
		Sotrovimab	9.45 (0.22 – 397.35)	NC	0.12 (0.03 – 0.49)	20.8	0.87 (0.38 – 1.96)	NC
	30 days	Casirivimab-imdevimab	0.65 (0.43 – 0.98)	NC	0.71 (0.55 – 0.91)	NC	0.14 (0.03 – 0.62)	28.4
		Bamlanivimab-etesevimab	0.42 (0.02 – 7.28)	NC	0.79 (0.39 – 1.57)	NC	1.72 (0.66 – 4.47)	NC
		Sotrovimab	7.96 (0.19 – 334.29)	NC	0.16 (0.05 – 0.50)	18.2	0.65 (0.31 – 1.34)	NC
Hospitalization	14 days	Casirivimab-imdevimab	0.08 (0.03 – 0.21)	10.6	0.29 (0.20 – 0.42)	24.3	0.05 (0.01 – 0.42)	45.8
		Bamlanivimab-etesevimab	0.37 (0.02 – 6.34)	NC	0.32 (0.08 – 1.25)	NC	0.89 (0.20 – 4.01)	NC
		Sotrovimab	6.94 (0.17 – 291.34)	NC	0.12 (0.03 – 0.51)	19.4	0.60 (0.19 – 1.87)	NC
	30 days	Casirivimab-imdevimab	0.09 (0.04 – 0.21)	9.5	0.33 (0.24 – 0.47)	22.5	0.18 (0.04 – 0.84)	NC
		Bamlanivimab-etesevimab	0.32 (0.02 – 5.56)	NC	0.36 (0.12 – 1.07)	NC	1.32 (0.38 – 4.63)	NC
		Sotrovimab	6.09 (0.15 – 255.49)	NC	0.17 (0.05 – 0.56)	17.9	2.10 (1.06 – 4.15)	NC
Death	14 days	Casirivimab-imdevimab	0.24 (0.01 – 4.52)	NC	0.10 (0.02 – 0.52)	287.3	0.19 (0.01 – 3.22)	NC
		Bamlanivimab-etesevimab	10.55 (0.54 – 206.10)	NC	0.56 (0.03 – 9.21)	NC	1.17 (0.07 – 20.23)	NC
		Sotrovimab	Non-convergence	NC	2.48 (0.15 – 40.86)	NC	0.11 (0.01 – 1.85)	NC
	30 days	Casirivimab-imdevimab	0.07 (0.00 – 1.17)	NC	0.05 (0.01 – 0.26)	139.2	0.17 (0.01 – 2.93)	NC
		Bamlanivimab-etesevimab	3.03 (0.17 – 53.66)	NC	0.29 (0.02 – 4.65)	NC	1.07 (0.06 – 18.38)	NC
		Sotrovimab	Non-convergence	NC	1.26 (0.08 – 20.63)	NC	0.10 (0.01 – 1.68)	NC
Hospitalization or death	14 days	Casirivimab-imdevimab	0.08 (0.03 – 0.21)	10.3	0.28 (0.20 – 0.41)	23.1	0.05 (0.01 – 0.41)	45.1
		Bamlanivimab-etesevimab	0.36 (0.02 – 6.19)	NC	0.30 (0.08 – 1.20)	NC	0.88 (0.20 – 3.94)	NC
		Sotrovimab	6.77 (0.16 – 284.33)	NC	0.12 (0.03 – 0.49)	18.5	0.59 (0.19 – 1.83)	NC
	30 days	Casirivimab-imdevimab	0.09 (0.04 – 0.20)	9.3	0.32 (0.23 – 0.44)	20.9	0.18 (0.04 – 0.83)	NC

© 2023 Ambrose N et al. *JAMA Network Open*.

			Delta 21AI		Delta 21J		Omicron BA.1	
**Outcome**	**Endpoint**	**mAb Product**	**Odds Ratio (95% CI)**	**NNT**	**Odds Ratio (95% CI)**	**NNT**	**Odds Ratio (95% CI)**	**NNT**
		Bamlanivimab-etesevimab	0.32 (0.02 – 5.44)	NC	0.34 (0.11 – 1.01)	NC	1.31 (0.37 – 4.57)	NC
		Sotrovimab	5.96 (0.14 – 249.92)	NC	0.16 (0.05 – 0.53)	16.8	2.07 (1.05 – 4.09)	NC

© 2023 Ambrose N et al. *JAMA Network Open*.

eAppendix. Sensitivity Analyses

The findings of increased 30-day hospitalization rates in treated patients in the Omicron BA.1 variant epoch (January 2022) and with an Omicron BA.1 variant sequence were unexpected and initiated post-hoc sensitivity analyses to confirm the results. For simplicity, all sensitivity analyses in this section were performed in the first imputation group.

Covariate Balance Within Variant Epochs

Health systems reported that the nMAb supply shortages in January 2022 required them to only treat patients most at risk for COVID-19 disease progression. Given the treatment decision in January 2022 was influenced by supply chain issues not observed in other study periods, the covariate balance by variant epoch using the stabilized weights was assessed to see if the weighting procedure did not adequately control for confounding in the Omicron BA.1 epoch (January 2022). To assess covariate balance, the mean and maximum absolute standardized mean difference (SMD) and Kolmogorov-Smirnoff (KS) test statistic were calculated to compare the covariate distributions after weighting by treatment status (eTable 20). The covariate balance in the Omicron BA.1 epoch was similar to other time periods in the study, suggesting that covariates were adequately controlled across epochs using a single propensity model.

eTable 20. Mean and Maximum Absolute SMD and KS Test Distance Comparing the Covariate Balance by Variant Epoch Using Single Propensity Model

Variant Epoch	Mean SMD	Max SMD	Mean KS distance	Max KS distance
Pre-Delta (2020-11 to 2021-06)	0.0270	0.2962	0.0264	0.2294
Delta (2021-07 to 2021-11)	0.0209	0.1343	0.0208	0.1449
Delta/Omicron BA.1 (2021-12)	0.0314	0.4344	0.0287	0.2856
Omicron BA.1 (2022-01)	0.0251	0.1462	0.0255	0.1462

Although a single model adequately balanced the covariates, to test the sensitivity of using a single propensity model across all variant epochs in modeling treatment effectiveness, within each variant epoch a propensity model was fit and tested for covariate balance, and nMAb treatment effectiveness was estimated using MSMs. Propensity models built within the Delta and Delta/Omicron BA.1 epochs balanced the covariates better than in the pre-Delta and Omicron BA.1 epochs (eTable 21); however, the balance within the Omicron BA.1 epoch using a propensity model fit with only patients in the Omicron BA.1 epoch did not appreciably improve the balance compared to using the single propensity model in the main study.

© 2023 Ambrose N et al. *JAMA Network Open*.

eTable 21. Mean and Maximum Absolute SMD and KS Test Distance Comparing the Covariate Balance by Variant Epoch Using Variant Epoch–Specific Propensity Models

Variant Epoch	Mean SMD	Max SMD	Mean KS distance	Max KS distance
Pre-Delta (2020-11 to 2021-06)	0.0259	0.3863	0.0215	0.1710
Delta (2021-07 to 2021-11)	0.0046	0.0757	0.0048	0.0511
Delta/Omicron BA.1 (2021-12)	0.0060	0.0803	0.0056	0.0803
Omicron BA.1 (2022-01)	0.0206	0.1318	0.0211	0.1361

Further, when estimating treatment effectiveness using weights derived by propensity models fit within each variant epoch, the results coincide with the main study results (eTable 22). In particular, the odds of 30-day hospitalizations and composite outcomes were still significantly higher in the treated than non-treated patients in the Omicron BA.1 epoch. In the Omicron BA.1 epoch, this analysis was repeated using just patients treated with sotrivimab and the results were similar. For example, the odds of 30-day hospitalization were 1.82 (95% CI = 1.13 – 2.92) times higher in sotrovimab patients than the non-treated patients.

eTable 22. Effectiveness Results Using Propensity Models Developed Within Each Variant Epoch

Variant epoch	Outcome	Endpoint	Odds Ratio	Odds Ratio 95% CI
Pre-Delta (2020-11 to 2021-06)	Emergency department visit	14-day	0.85	0.69 – 1.04
		30-day	0.93	0.77 – 1.11
	Hospitalization	14-day	0.70	0.57 – 0.86
		30-day	0.75	0.62 – 0.90
	Mortality (all-cause)	14-day	0.35	0.10 – 1.25
		30-day	0.27	0.11 – 0.66
	Composite: Hospitalization or mortality	14-day	0.69	0.56 – 0.85
		30-day	0.73	0.61 – 0.88
Delta (2021-07 to 2021-11)	Emergency department visit	14-day	0.79	0.71 – 0.89
		30-day	0.87	0.79 – 0.97
	Hospitalization	14-day	0.49	0.42 – 0.56
		30-day	0.57	0.50 – 0.65
	Mortality (all-cause)	14-day	0.19	0.09 – 0.38
		30-day	0.19	0.12 – 0.30
	Composite: Hospitalization or mortality	14-day	0.46	0.40 – 0.53
		30-day	0.54	0.48 – 0.61
Delta/Omicron BA.1 (2021-12)	Emergency department visit	14-day	0.76	0.56 – 1.02
		30-day	0.93	0.73 – 1.20
	Hospitalization	14-day	0.80	0.59 – 1.09
		30-day	1.02	0.77 – 1.34
	Mortality (all-cause)	14-day	0.09	0.01 – 0.65
		30-day	0.14	0.04 – 0.45
		14-day	0.77	0.57 – 1.04

© 2023 Ambrose N et al. *JAMA Network Open*.

Variant epoch	Outcome	Endpoint	Odds Ratio	Odds Ratio 95% CI
	Composite: Hospitalization or mortality	30-day	0.96	0.73 – 1.26
Omicron BA.1 (2022-01)	Emergency department visit	14-day	0.35	0.19 – 0.64
		30-day	0.43	0.27 – 0.70
	Hospitalization	14-day	0.99	0.51 – 1.94
		30-day	1.70	1.07 – 2.69
	Mortality (all-cause)	14-day	0.00	0.00 – 0.00
		30-day	0.06	0.01 – 0.42
	Composite: Hospitalization or mortality	14-day	0.94	0.48 – 1.85
		30-day	1.63	1.03 – 2.57
Omicron BA.1 (2022-01) and only Sotrovimab treated patients	Emergency department visit	14-day	0.32	0.17 – 0.61
		30-day	0.38	0.23 – 0.63
	Hospitalization	14-day	1.06	0.53 – 2.13
		30-day	1.82	1.13 – 2.92
	Mortality (all-cause)	14-day	0.00	0.00 – 0.00
		30-day	0.05	0.01 – 0.39
	Composite: Hospitalization or mortality	14-day	1.01	0.50 – 2.02
		30-day	1.74	1.08 – 2.79

Exact Matching for Treatment Effectiveness

To compare with the nMAb treatment effectiveness results using MSMs, exact matching was performed using the *MatchIt* package with sandwich standard errors to calculate significance and confidence intervals. Two different sets of variables were used to match treated with non-treated: Set 1 were variables found be strongly associated with treatment and strong effect modifiers. Set 2 included set 1 variables but also included additional variables that were among the top variables associated with treatment in the propensity model.

- **Set 1:** Health system, age groups (12-20, 20-30, 30-40, etc.), COVID-19 immunization status, DRS category (0-20, 20-40, 40-60, 60-80, and 80+), and immunocompromised status (binary yes/no), and diagnosis epoch
- **Set 2**: Variables in set 1 plus pregnancy status, birth sex, arthropathies, diabetes without complication, insurance, out of state, and smoking status.

Using set 1 matching variables confirmed the treatment effectiveness in the pre-Delta and Delta epoch (eTable 23). For 30-day hospitalizations in the Omicron BA.1 epoch, the direction of association was similar to those reported in the main study, but the results were not statistically significant when using set 1 variables with exact matching. Interestingly, when using set 2 variables, the exact matching results show a protective association of treatment in the Omicron BA.1 epoch, albeit not significant (eTable 24). For example, the odds of 30-day hospitalizations were 0.91 (95% CI: 0.50 – 1.64) times lower in the treated versus non-treated patients. Of note, the sample sizes are reduced given some treated and non-treated could not be matched in this procedure, which influences the power to detect a statistical association.

© 2023 Ambrose N et al. *JAMA Network Open*.

eTable 23. Effectiveness Results Using Exact Matching Procedure With Set 1 Matching Variables

Variant epoch	Outcome	Endpoint	Odds Ratio	Odds Ratio 95% CI
Pre-Delta (2020-11 to 2021-06)<br><br>7,034 treated/ 34,672 non-treated	Emergency department visit	14-day	0.72	0.56 – 0.93
		30-day	0.76	0.61 – 0.95
	Hospitalization	14-day	0.51	0.39 – 0.66
		30-day	0.56	0.44 – 0.70
	Mortality (all-cause)	14-day	0.10	0.04 – 0.27
		30-day	0.12	0.06 – 0.22
	Composite: Hospitalization or mortality	14-day	0.49	0.38 – 0.64
		30-day	0.53	0.42 – 0.67
Delta (2021-07 to 2021-11)<br><br>13,632 treated/ 27,767 non-treated	Emergency department visit	14-day	0.74	0.65 – 0.84
		30-day	0.80	0.72 – 0.89
	Hospitalization	14-day	0.49	0.40 – 0.59
		30-day	0.55	0.47 – 0.65
	Mortality (all-cause)	14-day	0.16	0.08 – 0.30
		30-day	0.20	0.13 – 0.31
	Composite: Hospitalization or mortality	14-day	0.46	0.38 – 0.56
		30-day	0.53	0.45 – 0.62
Delta/Omicron BA.1 (2021-12)<br><br>2,638 treated/ 14,145 non-treated	Emergency department visit	14-day	0.76	0.56 – 1.02
		30-day	0.91	0.71 – 1.16
	Hospitalization	14-day	0.70	0.49 – 1.00
		30-day	0.90	0.67 – 1.23
	Mortality (all-cause)	14-day	0.09	0.01 – 0.66
		30-day	0.16	0.05 – 0.55
	Composite: Hospitalization or mortality	14-day	0.67	0.47 – 0.94
		30-day	0.85	0.63 – 1.15
Omicron BA.1 (2022-01)<br><br>1,038 treated 32,037 non-treated	Emergency department visit	14-day	0.57	0.19 – 1.68
		30-day	0.55	0.25 – 1.23
	Hospitalization	14-day	1.09	0.57 – 2.11
		30-day	1.59	0.95 – 2.67
	Mortality (all-cause)	14-day	0.00	0.00 – 0.00
		30-day	0.06	0.01 – 0.41
	Composite: Hospitalization or mortality	14-day	1.03	0.54 – 1.99
		30-day	1.52	0.90 – 2.54

© 2023 Ambrose N et al. *JAMA Network Open*.

eTable 24. Effectiveness Results Using Exact Matching Procedure With Set 2 Matching Variables

Variant epoch	Outcome	Endpoint	Odds Ratio	Odds Ratio 95% CI
Pre-Delta (2020-11 to 2021-06)<br><br>4,739 treated/ 19,448 non-treated	Emergency department visit	14-day	0.75	0.59 – 0.97
		30-day	0.83	0.66 – 1.04
	Hospitalization	14-day	0.49	0.39 – 0.63
		30-day	0.54	0.44 – 0.68
	Mortality (all-cause)	14-day	0.12	0.04 – 0.42
		30-day	0.16	0.07 – 0.35
	Composite: Hospitalization or mortality	14-day	0.48	0.38 – 0.61
		30-day	0.53	0.43 – 0.66
Delta (2021-07 to 2021-11)<br><br>9,097 treated/ 18,129 non-treated	Emergency department visit	14-day	0.81	0.69 – 0.95
		30-day	0.86	0.75 – 0.98
	Hospitalization	14-day	0.46	0.37 – 0.58
		30-day	0.54	0.44 – 0.66
	Mortality (all-cause)	14-day	0.11	0.04 – 0.30
		30-day	0.18	0.09 – 0.34
	Composite: Hospitalization or mortality	14-day	0.44	0.35 – 0.56
		30-day	0.52	0.43 – 0.64
Delta/Omicron BA.1 (2021-12)<br><br>1,939 treated/ 8,532 non-treated	Emergency department visit	14-day	0.86	0.60 – 1.25
		30-day	1.04	0.75 – 1.45
	Hospitalization	14-day	0.65	0.44 – 0.98
		30-day	1.12	0.57 – 2.20
	Mortality (all-cause)	14-day	0.13	0.02 – 1.04
		30-day	0.06	0.01 – 0.42
	Composite: Hospitalization or mortality	14-day	0.64	0.43 – 0.95
		30-day	1.06	0.54 – 2.08
Omicron BA.1 (2022-01)<br><br>653 treated/ 10,705 non-treated	Emergency department visit	14-day	0.46	0.11 – 1.94
		30-day	0.47	0.16 – 1.38
	Hospitalization	14-day	0.68	0.27 – 1.70
		30-day	0.91	0.50 – 1.64
	Mortality (all-cause)	14-day	0.00	0.00 – 0.00
		30-day	0.00	0.00 – 0.00
	Composite: Hospitalization or mortality	14-day	0.66	0.26 – 1.66
		30-day	0.89	0.49 – 1.60

© 2023 Ambrose N et al. *JAMA Network Open*.

Use of Immunocompromised Subclasses

Participating health systems reported that the mAb supply shortages in January 2022 required them to only treat patients most at risk for COVID-19 disease progression. Each health system reported specifically targeting immunocompromised patients and tiered the immunocompromised patients into risk groups. The immunocompromised subclass definitions were gathered from all four health systems and reconciled into a single tiered system. The health systems were provided the subclassification definitions and asked to label each patient according to the scheme represented in eAppendix Table 7.

eTable 25. Subclassification of Immunocompromised States

**Subclass 1**	• Lung transplant recipient (any time frame)<br>Receipt of the following immunosuppressive medication within the past 12 months (including for solid organ transplant)<br>o Anti-thymocyte globulin (ATG)<br>o Alemtuzumab<br>o Anti-B-cell therapy (e.g., rituximab)<br>• B-cell malignancies, on active treatment (e.g., B-cell lymphomas, chronic lymphocytic leukemia, acute B-cell lymphoblastic leukemia, etc.)<br>• Multiple myeloma, on *active treatment with two or more agents<br>• Allogeneic stem cell transplant, within 12 months of transplant<br>• Autologous stem cell transplant, within 6 months of transplant<br>• Receipt of anti-CD19 or anti-BCMA (CAR)-T-cell immunotherapy, within 6 months of treatment<br>• Primary or secondary T-cell or B-cell immunodeficiency, including severe combined variable immunodeficiency (CVID)<br>• Recipient of more than one active transplant, different organs (any time frame)<br>o Example: kidney-pancreas, heart-kidney<br>• Acute myeloid leukemia under *active treatment
**Subclass 2**	• Any solid organ transplant <u>within the past 12 months</u> from date of transplant, not otherwise eligible in Category 1<br>• Allogeneic stem cell transplant, 12 – 24 months since transplant<br>• Autologous stem cell transplant, 6 – 24 months since transplant<br>• Allogeneic or autologous stem cell transplant >24 months and still *actively on immunosuppressive therapy<br>• Any solid tumor, on active myelosuppressive chemotherapy<br>• Multiple myeloma, on **maintenance therapy
**Subclass 3**	• *Active treatment with high-dose corticosteroids (i.e., ≥20 mg prednisone or equivalent per day when administered for ≥2 weeks)<br>• *Active treatment with other biologic agents that are immunosuppressive or immunomodulatory, not otherwise listed in Categories 1-3<br>• Any solid organ transplant recipient <u>more than 12 months</u> since transplant not otherwise eligible in Subclass 1-2<br>• Advanced or untreated HIV infection<br>o HIV with CD4<200/mm3 (if <14 years, CD4% <15%)<br>o AIDS-defining illness (within 12 months)

© 2023 Ambrose N et al. *JAMA Network Open*.

* “Active treatment” includes any patient with a documented drug exposure to one of the defined agents within the 90 days leading up to day 0 (the index date).
**“Maintenance therapy”: “*Treatment that is given to help keep cancer from coming back after it has disappeared following the initial therapy. It may include treatment with drugs, vaccines, or antibodies that kill cancer cells, and it may be given for a long time.*” https://www.cancer.gov/publications/dictionaries/cancer-terms/def/maintenance-therapy . Note that maintenance therapy can include agents that were also used in the initial therapy with the difference being the relative dose or frequency that is given to the patient.

The use of these refined immunocompromised subclasses instead of the binary (yes/no) immunocompromised status variable was tested to confirm the main study effectiveness results. To do this, the immunocompromised subclass variable was used as 1) a variable within the propensity model developed within each variant epoch used to make stabilized weights within the MSMs and 2) a matching variable used in the exact matching procedure. The use of the immunocompromised subclasses in propensity scores developed within each variant epoch generally coincide with the results of the main study. In particular, the odds of 30-day hospitalizations in the Omicron BA.1 epoch are still statistically higher in the treated compared to the non-treated patients (eTable 26).

eTable 26. Effectiveness Results Using Propensity Models Developed Within Each Variant Epoch and Immunocompromised Subclasses

Variant epoch	Outcome	Endpoint	Odds Ratio	Odds Ratio 95% CI
Pre-Delta (2020-11 to 2021-06)	Emergency department visit	14-day	0.85	0.70 – 1.04
		30-day	0.93	0.78 – 1.12
	Hospitalization	14-day	0.68	0.55 – 0.84
		30-day	0.74	0.61 – 0.90
	Mortality (all-cause)	14-day	0.33	0.09 – 1.19
		30-day	0.26	0.10 – 0.64
	Composite: Hospitalization or mortality	14-day	0.67	0.54 – 0.83
		30-day	0.73	0.60 – 0.88
Delta (2021-07 to 2021-11)	Emergency department visit	14-day	0.79	0.70 – 0.89
		30-day	0.88	0.79 – 0.97
	Hospitalization	14-day	0.49	0.42 – 0.56
		30-day	0.57	0.50 – 0.65
	Mortality (all-cause)	14-day	0.17	0.09 – 0.35
		30-day	0.18	0.11 – 0.29
	Composite: Hospitalization or mortality	14-day	0.46	0.40 – 0.54
		30-day	0.54	0.47 – 0.61
Delta/Omicron BA.1 (2021-12)	Emergency department visit	14-day	0.76	0.56 – 1.02
		30-day	0.93	0.73 – 1.20
	Hospitalization	14-day	0.80	0.59 – 1.09
		30-day	1.02	0.77 – 1.34
	Mortality (all-cause)	14-day	0.07	0.01 – 0.55
		30-day	0.13	0.04 – 0.43

© 2023 Ambrose N et al. *JAMA Network Open*.

Variant epoch	Outcome	Endpoint	Odds Ratio	Odds Ratio 95% CI
	Composite: Hospitalization or mortality	14-day	0.76	0.56 – 1.03
		30-day	0.96	0.73 – 1.26
Omicron BA.1 (2022-01)	Emergency department visit	14-day	0.34	0.18 – 0.63
		30-day	0.43	0.27 – 0.71
	Hospitalization	14-day	0.96	0.49 – 1.90
		30-day	1.64	1.02 – 2.62
	Mortality (all-cause)	14-day	0.00	0.00 – 0.00
		30-day	0.07	0.01 – 0.47
	Composite: Hospitalization or mortality	14-day	0.92	0.46 – 1.81
		30-day	1.57	0.98 – 2.51

The matching procedure found approximately the same number of appropriate treated and non-treated matching using the immunocompromised subclasses (eTables 27 and eTable 28) compared to matching using the binary immunocompromised status variable. Focusing on the Omicron BA.1 epoch, treatment was only significantly associated with reduced mortality at 14 days using set 1 and set 2 matching variables. Similar to using set 2 variables with the binary immunocompromised status variable, matching with set 2 variables and the immunocompromised subclass variable changed the direction of association for 30-day hospitalizations in the Omicron BA.1 epoch, albeit not significantly.

eTable 27. Effectiveness Results Using Exact Matching Procedure With Set 1 Matching Variables, Substituting Immunocompromised Status With Immunocompromised Subclass

Variant epoch	Outcome	Endpoint	Odds Ratio	Odds Ratio 95% CI
Pre-Delta (2020-11 to 2021-06)<br><br>6,934 treated/ 34,389 non-treated	Emergency department visit	14-day	0.68	0.53 – 0.87
		30-day	0.70	0.56 – 0.86
	Hospitalization	14-day	0.51	0.39 – 0.67
		30-day	0.53	0.42 – 0.66
	Mortality (all-cause)	14-day	0.10	0.04 – 0.26
		30-day	0.12	0.07 – 0.21
	Composite: Hospitalization or mortality	14-day	0.49	0.38 – 0.64
		30-day	0.51	0.40 – 0.63
Delta (2021-07 to 2021-11)<br><br>13,534 treated/ 27,356 non-treated	Emergency department visit	14-day	0.76	0.67 – 0.87
		30-day	0.82	0.73 – 0.91
	Hospitalization	14-day	0.47	0.39 – 0.58
		30-day	0.54	0.46 – 0.64
	Mortality (all-cause)	14-day	0.15	0.08 – 0.30
		30-day	0.20	0.13 – 0.31
	Composite: Hospitalization or mortality	14-day	0.45	0.37 – 0.55
		30-day	0.52	0.44 – 0.61
	Emergency department visit	14-day	0.75	0.55 – 1.02

© 2023 Ambrose N et al. *JAMA Network Open*.

Variant epoch	Outcome	Endpoint	Odds Ratio	Odds Ratio 95% CI
Delta/Omicron BA.1 (2021-12)<br><br>2,577 treated/ 14,037 non-treated		30-day	0.91	0.71 – 1.17
	Hospitalization	14-day	0.72	0.49 – 1.05
		30-day	0.93	0.68 – 1.27
	Mortality (all-cause)	14-day	0.00	0.00 – 0.00
		30-day	0.10	0.02 – 0.46
	Composite: Hospitalization or mortality	14-day	0.67	0.46 – 0.99
		30-day	0.87	0.63 – 1.18
Omicron BA.1 (2022-01)<br><br>965 treated 31,407 non-treated	Emergency department visit	14-day	0.57	0.18 – 1.78
		30-day	0.62	0.27 – 1.39
	Hospitalization	14-day	0.89	0.49 – 1.61
		30-day	1.38	0.77 – 2.46
	Mortality (all-cause)	14-day	0.00	0.00 – 0.00
		30-day	0.32	0.06 – 1.80
	Composite: Hospitalization or mortality	14-day	0.87	0.43 – 1.74
		30-day	1.28	0.72 – 2.29

© 2023 Ambrose N et al. *JAMA Network Open*.

eTable 28. Effectiveness Results Using Exact Matching Procedure With Set 2 Matching Variables, Substituting Immunocompromised Status With Immunocompromised Subclass

Variant epoch	Outcome	Endpoint	Odds Ratio	Odds Ratio 95% CI
Pre-Delta (2020-11 to 2021-06)<br><br>4,895 treated/ 18,897 non-treated	Emergency department visit	14-day	0.77	0.60 – 0.99
		30-day	0.82	0.66 – 1.02
	Hospitalization	14-day	0.48	0.37 – 0.61
		30-day	0.51	0.41 – 0.64
	Mortality (all-cause)	14-day	0.07	0.02 – 0.31
		30-day	0.12	0.05 – 0.30
	Composite: Hospitalization or mortality	14-day	0.46	0.36 – 0.59
		30-day	0.50	0.40 – 0.62
Delta (2021-07 to 2021-11)<br><br>9,274 treated/ 17,812 non-treated	Emergency department visit	14-day	0.84	0.71 – 0.99
		30-day	0.89	0.77 – 1.02
	Hospitalization	14-day	0.46	0.37 – 0.59
		30-day	0.54	0.44 – 0.67
	Mortality (all-cause)	14-day	0.09	0.03 – 0.27
		30-day	0.18	0.10 – 0.33
	Composite: Hospitalization or mortality	14-day	0.44	0.35 – 0.56
		30-day	0.53	0.43 – 0.64
Delta/Omicron BA.1 (2021-12)<br><br>1,953 treated/ 8,550 non-treated	Emergency department visit	14-day	0.87	0.60 – 1.28
		30-day	1.07	0.77 – 1.50
	Hospitalization	14-day	0.62	0.41 – 0.93
		30-day	1.11	0.57 – 2.17
	Mortality (all-cause)	14-day	0.00	0.00 – 0.00
		30-day	0.13	0.03 – 0.55
	Composite: Hospitalization or mortality	14-day	0.59	0.40 – 0.89
		30-day	1.05	0.54 – 2.05
Omicron BA.1 (2022-01)<br><br>631 treated/ 10,420 non-treated	Emergency department visit	14-day	0.54	0.13 – 2.19
		30-day	0.53	0.18 – 1.51
	Hospitalization	14-day	0.50	0.23 – 1.07
		30-day	0.66	0.38 – 1.15
	Mortality (all-cause)	14-day	0.00	0.00 – 0.00
		30-day	0.18	0.02 – 1.45
	Composite: Hospitalization or mortality	14-day	0.48	0.23 – 1.04
		30-day	0.64	0.37 – 1.11

© 2023 Ambrose N et al. *JAMA Network Open*.

Test for Immortal Bias

The influence of immortal time bias, or the scenario when the outcome cannot occur between the diagnosis and treatment and thus biases the results in favor of the treated**,** was tested in a sensitivity analysis by shifting the index date of the treated group to the day of nMAb treatment.[28] Results from our sensitivity analysis exploring the influence of immortal time bias did not change our main findings that nMAb treatment significantly reduced the odds of experiencing the study outcomes.

eTable 29. Overall nMAb Treatment Effectiveness Testing the Influence of Immortal Time Bias. Odds Ratios, Probabilities, 95% Confidence Intervals (CI), and Number Needed to Treat (NNT) from Marginal Structural Models Adjusted for Confounders. Index Date of Treated Shifted to the Day of nMAb Treatment.

Outcome	Endpoint	Odds Ratio (95% CI)	Non-treated Probability, % (95% CI)	Treated Probability, % (95% CI)	NNT
**Emergency department visit**	14-day	0.83 (0.74 - 0.93)	4.6 (4.3 - 5.0)	3.9 (3.6 - 4.2)	128.5
	30-day	0.85 (0.77 - 0.93)	5.9 (5.6 - 6.3)	5.1 (4.8 - 5.5)	116.9
**Hospitalization**	14-day	0.56 (0.50 - 0.64)	4.9 (4.6 - 5.2)	2.8 (2.5 - 3.1)	47.7
	30-day	0.65 (0.58 - 0.73)	5.9 (5.5 - 6.2)	3.9 (3.5 - 4.3)	51.0
**Mortality (all-cause)**	14-day	0.16 (0.10 - 0.25)	0.5 (0.4 - 0.6)	0.1 (0.0 - 0.1)	256.0
	30-day	0.17 (0.11 - 0.26)	0.9 (0.7 - 1.1)	0.2 (0.1 - 0.2)	135.7
**Composite: hospitalization or mortality**	14-day	0.53 (0.47 - 0.60)	5.3 (4.9 - 5.6)	2.9 (2.6 - 3.2)	41.8
	30-day	0.61 (0.55 - 0.69)	6.3 (6.0 - 6.7)	4.0 (3.6 - 4.4)	42.7

© 2023 Ambrose N et al. *JAMA Network Open*.

eReferences

1. AHRQ. Elixhauser Comorbidity Software Refined for ICD-10-CM diagnosis, v2022.1. 2021;Available from: https://www.hcup-us.ahrq.gov/toolssoftware/comorbidityicd10/CMR-User-Guide-v2022-1.pdf

2. United States Census Bureau. 2019 American Community Survey. [cited 2022 Feb 18];Available from: https://www.census.gov/acs/www/data/data-tables-and-tools/data-profiles/

3. Kind AJH, Buckingham WR. Making Neighborhood-Disadvantage Metrics Accessible — The Neighborhood Atlas. New England Journal of Medicine [Internet] 2018 [cited 2022 Mar 4];378(26):2456–8. Available from: https://doi.org/10.1056/NEJMp1802313

4. University of Wisconsin School of Medicine and Public Health. 2019. Area Deprivation Index, Version 3.1 [Internet]. [cited 2022 Feb 28];Available from: https://www.neighborhoodatlas.medicine.wisc.edu

5. Artic Network. Real-Time Molecular Epidemiology for Outbreak Response [Internet]. REAL-TIME MOLECULAR EPIDEMIOLOGY FOR OUTBREAK RESPONSE. 2022 [cited 2022 Mar 28];Available from: https://artic.network/

6. NCBI. ncbi/sra-human-scrubber [Internet]. 2021 [cited 2022 Mar 29];Available from: https://github.com/ncbi/sra-human-scrubber

7. Chen S, Zhou Y, Chen Y, Gu J. fastp: an ultra-fast all-in-one FASTQ preprocessor. Bioinformatics [Internet] 2018;34(17):i884–90. Available from: https://pubmed.ncbi.nlm.nih.gov/30423086/

8. NCBI. Severe acute respiratory syndrome coronavirus 2 (ID 86693) - Genome - NCBI [Internet]. 2022 [cited 2022 Mar 29];Available from: https://www.ncbi.nlm.nih.gov/genome/?term=sars-cov-2

9. Li H. Minimap2: pairwise alignment for nucleotide sequences. Bioinformatics 2018;34(18):3094–100.

10. Danecek P, Bonfield JK, Liddle J, et al. Twelve years of SAMtools and BCFtools. Gigascience 2021;10(2):giab008.

11. Artic Network. artic-ncov2019/primer_schemes/nCoV-2019/V3 at master · artic-network/artic-ncov2019 [Internet]. GitHub. 2020 [cited 2022 Mar 29];Available from: https://github.com/artic-network/artic-ncov2019

12. Grubaugh ND, Gangavarapu K, Quick J, et al. An amplicon-based sequencing framework for accurately measuring intrahost virus diversity using PrimalSeq and iVar. Genome Biol 2019;20(1):8.

© 2023 Ambrose N et al. *JAMA Network Open*.

13. Okonechnikov K, Conesa A, García-Alcalde F. Qualimap 2: advanced multi-sample quality control for high-throughput sequencing data. Bioinformatics 2016;32(2):292–4.

14. Anderson Lab. iVar: Manual [Internet]. [cited 2022 Mar 29];Available from: https://andersen-lab.github.io/ivar/html/manualpage.html

15. Naval Medical Research Center. Viral Amplicon Illumina Workflow [Internet]. 2022 [cited 2022 Mar 29];Available from: https://github.com/BDRD-Genomics/VAIW

16. Cingolani P, Platts A, Wang LL, et al. A program for annotating and predicting the effects of single nucleotide polymorphisms, SnpEff: SNPs in the genome of Drosophila melanogaster strain w1118; iso-2; iso-3. Fly (Austin) 2012;6(2):80–92.

17. Rambaut A, Holmes EC, O'Toole Á, et al. A dynamic nomenclature proposal for SARS-CoV-2 lineages to assist genomic epidemiology. Nat Microbiol [Internet] 2020 [cited 2022 Mar 29];5(11):1403–7. Available from: https://www.nature.com/articles/s41564-020-0770-5

18. Hodcroft E. CoVariants: 21A (Delta) [Internet]. 2022 [cited 2022 Mar 29];Available from: https://covariants.org/variants/21A.Delta

19. WHO. Tracking SARS-CoV-2 variants [Internet]. 2022 [cited 2022 Mar 28];Available from: https://www.who.int/health-topics/typhoid/tracking-SARS-CoV-2-variants

20. CDC. SARS-CoV-2 Variant Classifications and Definitions [Internet]. Centers for Disease Control and Prevention. 2020 [cited 2022 Mar 29];Available from: https://www.cdc.gov/coronavirus/2019-ncov/variants/variant-classifications.html

21. Hodcroft E. CoVariants: 21J (Delta) [Internet]. [cited 2022 May 9];Available from: https://covariants.org/variants/21J.Delta

22. Hahn G, Lutz S, Lange C. locStra: Fast Implementation of (Local) Population Stratification Methods version 1.8 from CRAN [Internet]. 2020 [cited 2022 Mar 29];Available from: https://rdrr.io/cran/locStra/?msclkid=3e63d13eaf7c11eca786ec489869400b

23. van Buuren S, Groothuis-Oudshoorn K. mice: Multivariate Imputation by Chained Equations in R. J Stat Soft [Internet] 2011 [cited 2021 Dec 10];45(3):1–67. Available from: https://www.jstatsoft.org/index.php/jss/article/view/v045i03

24. Moons KGM, Donders RART, Stijnen T, Harrell FE. Using the outcome for imputation of missing predictor values was preferred. Journal of Clinical Epidemiology [Internet] 2006 [cited 2022 Mar 28];59(10):1092–101. Available from: https://linkinghub.elsevier.com/retrieve/pii/S0895435606000606

25. Pedregosa F, Varoquaux G, Gramfort A, et al. Scikit-learn: Machine Learning in Python. Journal of Machine Learning Research [Internet] 2011 [cited 2022 Mar 15];12(85):2825–30. Available from: http://jmlr.org/papers/v12/pedregosa11a.html

© 2023 Ambrose N et al. *JAMA Network Open*.

26. Belitser SV, Martens EP, Pestman WR, Groenwold RHH, de Boer A, Klungel OH. Measuring balance and model selection in propensity score methods. Pharmacoepidemiology and Drug Safety [Internet] 2011 [cited 2021 Nov 9];20(11):1115–29. Available from: https://onlinelibrary.wiley.com/doi/abs/10.1002/pds.2188

27. Greifer N. Covariate Balance Tables and Plots: A Guide to the cobalt Package [Internet]. 2021 [cited 2021 Nov 9];Available from: https://cran.r-project.org/web/packages/cobalt/vignettes/cobalt.html

28. Zhou Z, Rahme E, Abrahamowicz M, Pilote L. Survival Bias Associated with Time-to-Treatment Initiation in Drug Effectiveness Evaluation: A Comparison of Methods. American Journal of Epidemiology [Internet] 2005 [cited 2023 Jan 29];162(10):1016–23. Available from: http://academic.oup.com/aje/article/162/10/1016/65057/Survival-Bias-Associated-with-TimetoTreatment

© 2023 Ambrose N et al. *JAMA Network Open*.

NOTICE

This (software/technical data) was produced for the U. S. Government under Contract Number 75FCMC18D0047, and is subject to Federal Acquisition Regulation Clause 52.227-14, Rights in Data-General.

No other use other than that granted to the U. S. Government, or to those acting on behalf of the U. S. Government under that Clause is authorized without the express written permission of The MITRE Corporation.

For further information, please contact The MITRE Corporation, Contracts Management Office, 7515 Colshire Drive, McLean, VA 22102-7539, (703) 983-6000.

© 2022 The MITRE Corporation
