# Supplementary material for: Neutralizing Monoclonal Antibody Use and COVID-19 Infection Outcomes
Source: JAMA Netw Open. 2023 Apr 24;6(4):e239694. doi: 10.1001/jamanetworkopen.2023.9694 (PMC10126875; doi:10.1001/jamanetworkopen.2023.9694)
Supplement: Supplement 2. — Data Sharing Statement [file jamanetwopen-e239694-s002.pdf]

# Data Sharing Statement

Ambrose. Neutralizing Monoclonal Antibody Use and COVID-19 Infection Outcomes. *JAMA Netw Open*. Published April 24, 2023. doi:10.1001/jamanetworkopen.2023.9694

**Data**
**Data available:** Yes
**Data types:** Deidentified participant data
**How to access data:** Deidentified data has been submitted to National COVID Cohort Collaborative (N3C). Available at https://ncats.nih.gov/n3c.
**When available:** With publication

**Supporting Documents**
**Document types:** Statistical/analytic code
**How to access documents:** Our analytical code has been published to GitHub and available at https://github.com/mitre/covid19-mabs-rwe.
**When available:** beginning date: 07-27-2022

**Additional Information**
**Who can access the data:** Data is available to researchers whose proposed use of the data has been approved by N3C.
**Types of analyses:** Data is available for purposes believed to have scientific merit as judged by N3C.
**Mechanisms of data availability:** Data is available to researchers after the approval of a proposal.
